# Supplementary material for: Potential Toxicity Risk Assessment and Priority Control Strategy for PAHs Metabolism and Transformation Behaviors in the Environment
Source: Int J Environ Res Public Health. 2022 Sep 2;19(17):10972. doi: 10.3390/ijerph191710972 (PMC9517862; doi:10.3390/ijerph191710972)
Supplement: Supplementary file 1 [file ijerph-19-10972-s001.zip › ijerph-1829876-supplementary.pdf]

Supplementary Materials

Table S1 Summary of PAH derivatives in environmental media or organisms

Parent PAHs	Metabolic type	Source, subject or condition	PAH derivatives	References
ACE	Microbial degradation	*Sphingobacterium sp.* strain RTSB	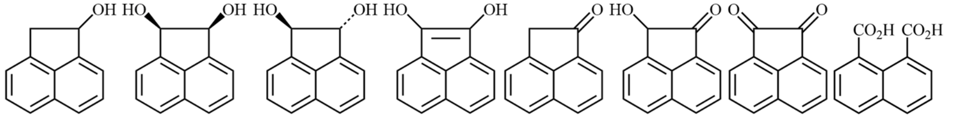 ACE-1 ACE-2 ACE-3 ACE-4 ACE-5 ACE-6 ACE-7 ACE-8 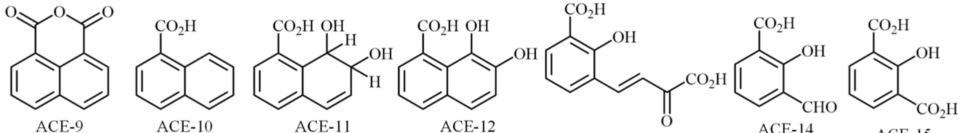 ACE-9 ACE-10 ACE-11 ACE-12 ACE-13 ACE-14 ACE-15 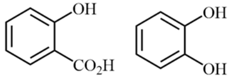 ACE-16 ACE-17	[1]
	Photolysis	temperature and daylight were 24 °C and 24 W/m^2	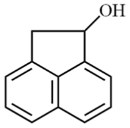 ACE-1	[2]
ACY	Microbial degradation	*Rhizobium sp.* Strain CU-A1	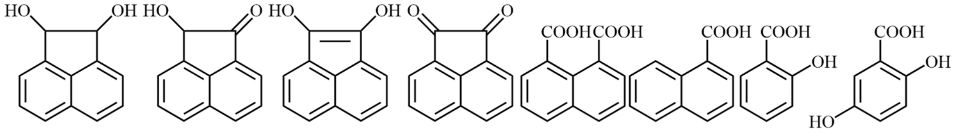 ACY-1 ACY-2 ACY-3 ACY-4 ACY-5 ACY-6 ACY-7 ACY-8	[3]

	Photolysis	ACY in a arenosol soil; temperature and daylight were 24 °C and 24 W/m^2	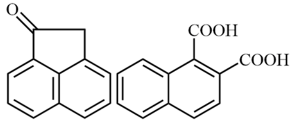 ACY-9 ACY-10	[2]
ANT	Photocatalytic degradation	photocatalyst Pt-tantalum oxynitride; under visible light irradiation	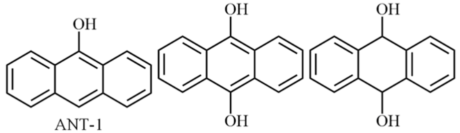 ANT-1 ANT-2 ANT-3	[4]
	Microbial degradation	filamentous fungus *Cyclothyrium sp.* CBS 109850 from estuarine sediment	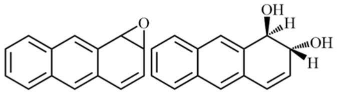 ANT-4 ANT-5	[5]
	Microbial degradation	ligninolytic fungus *Polyporus sp.* S133; ANT in a liquid medium	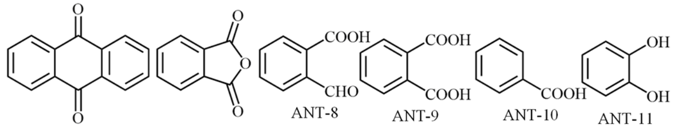 ANT-6 ANT-7 ANT-8 ANT-9 ANT-10 ANT-11	[6]
	Microbial degradation	*Bacillus thuringiensis* AT.ISM.1 isolated from a fly ash deposition site	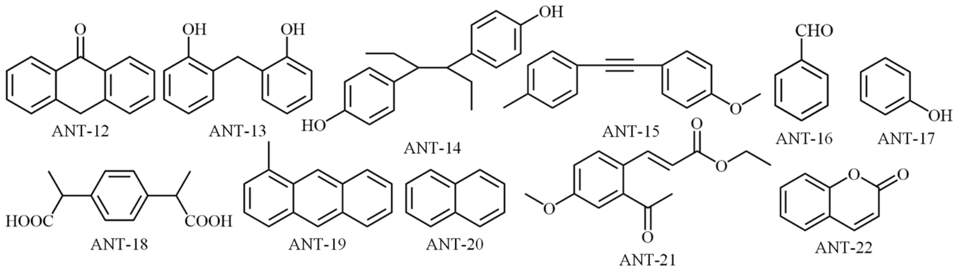 ANT-12 ANT-13 ANT-14 ANT-15 ANT-16 ANT-17 ANT-18 ANT-19 ANT-20 ANT-21 ANT-22	[7]

BaAN	Human metabolism	liver microsomes	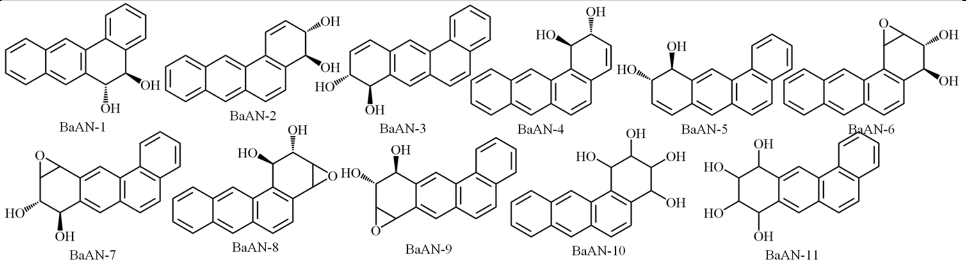	[8]
	Biological metabolism	chick embryos	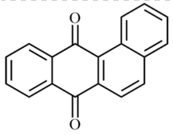 BaAN-12	[9]
	Photolysis	BaAN on Fe^{3+}-montmorillonite surface under visible light	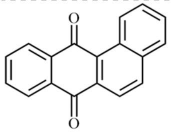 BaAN-12	[10]
	Microbial degradation	green alga *Chlamydomonas reinhardtii* CC-503	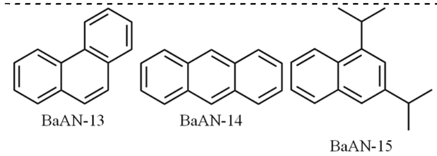	[11]

	Microbial degradation	ligninolytic fungus *I. lacteus*	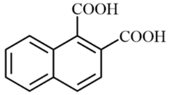 BaAN-16 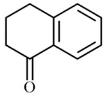 BaAN-17 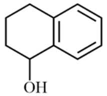 BaAN-18 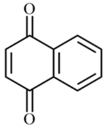 BaAN-19 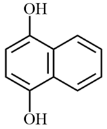 BaAN-20 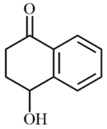 BaAN-21 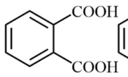 BaAN-22 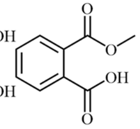 BaAN-23 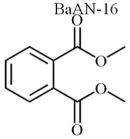 BaAN-24 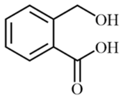 BaAN-25	[12]
	Photocatalytic degradation	photocatalyst zinc oxide encapsulated hexacyanoferrate nanocomposite; BaAN in water; sunlight exposure	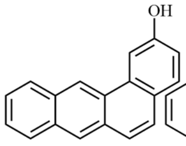 BaAN-26 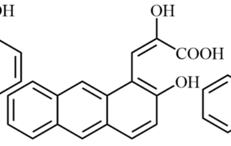 BaAN-27 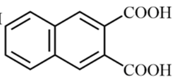 BaAN-28 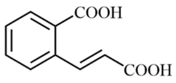 BaAN-29 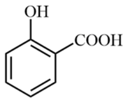 BaAN-30 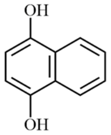 BaAN-20 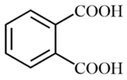 BaAN-22	[13]
BaP	Human metabolism	urine	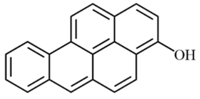 BaP-1	[14][15]
	Biological metabolism	fish bile	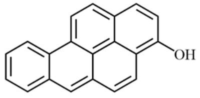 BaP-1	[16][17]

Photolysis	BaP on Fe^{3+}-montmorillonite surface under visible light	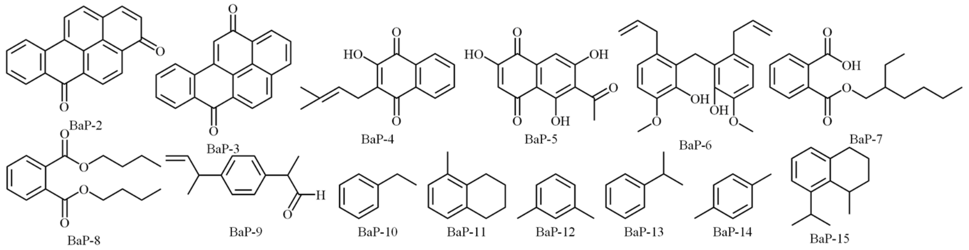	[10]
Photocatalytic degradation	iron oxide (goethite); UV radiation	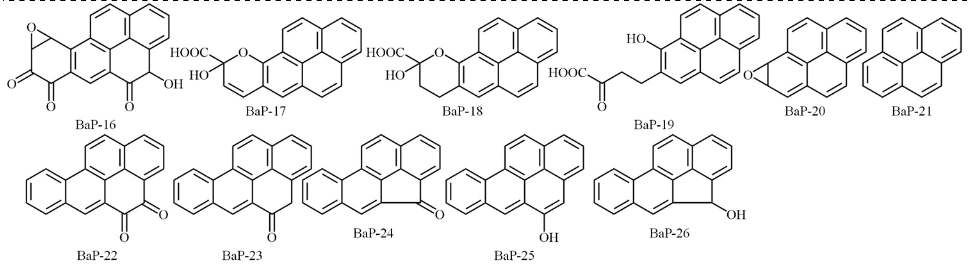	[18]
Resistance of plants	rooted poplar cuttings	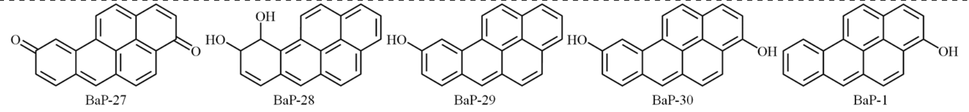	[19]
Microbial degradation	fungi (*Penicillium chrysogenum*)	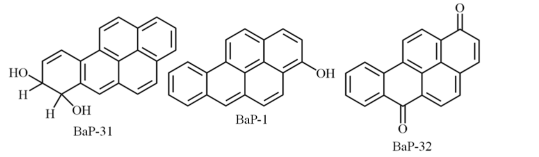	[20]

Anaerobic biodegradation	*Cellulosimicrobium cellulans* CWS2 isolated from polycyclic aromatic hydrocarbon-contaminated soil	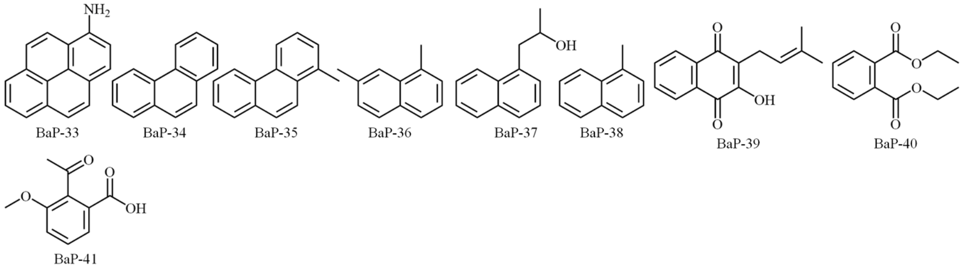	[21]
Microbial degradation	*Stenotrophomonas maltophilia* (copper and BaP combined contamination system)	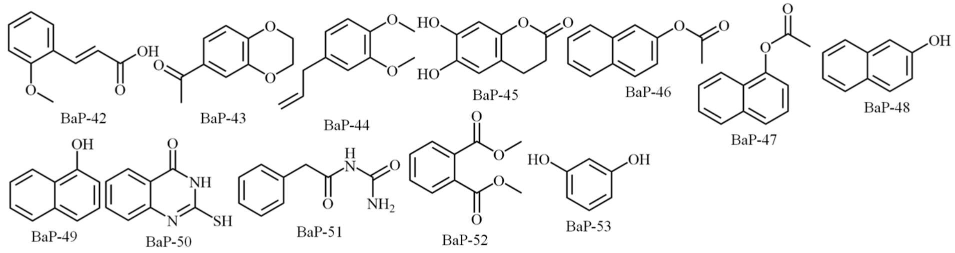	[22]
Microbial degradation	bacteria	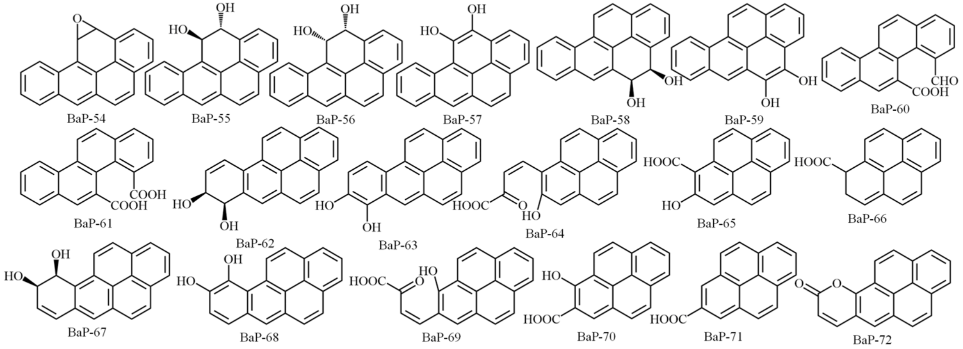	[23]
Microbial degradation	microalgae (*Selanastum capricornutum*)	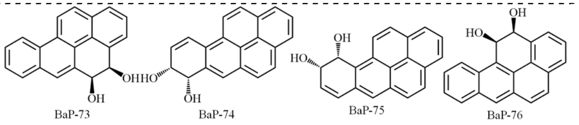	[24]

BbF	Biological metabolism	mouse epidermis	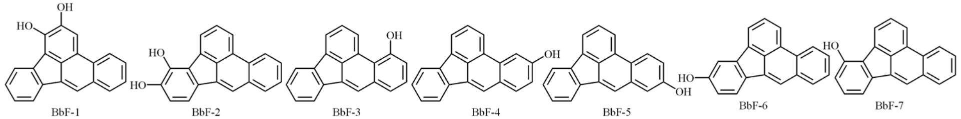 BbF-1 BbF-2 BbF-3 BbF-4 BbF-5 BbF-6 BbF-7	[25]
	Human metabolism	hair	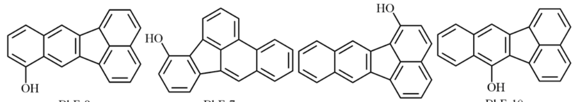 BbF-8 BbF-7 BbF-9 BbF-10	[26]
	Microbial degradation	*Sphingomonas paucimobilis* strain EPA505 isolated from a creosote waste site in Gulf Breeze	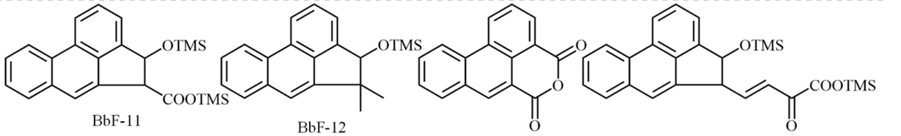 BbF-11 BbF-12 BbF-13 BbF-14	[27]
	Microbial degradation	freshwater microalgae *Selenastrum capricornutum*	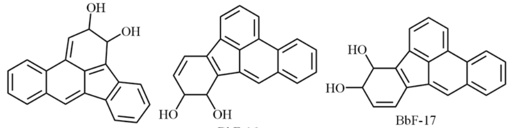 BbF-15 BbF-16 BbF-17	[28]
BghiP	Microbial degradation	yeast consortium isolated from PAHs contaminated site	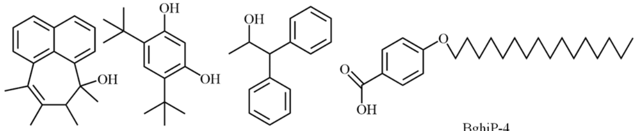 BghiP-1 BghiP-2 BghiP-3 BghiP-4	[29]

	Biological metabolism	rat liver microsomes	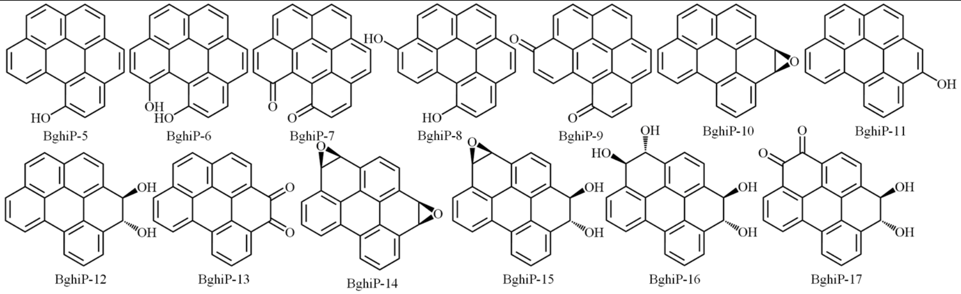	[30]
BkF	Biological metabolism	rat urine	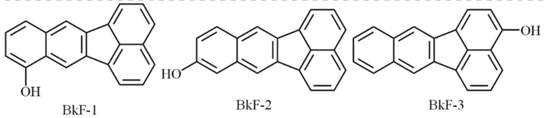	[31]
	Human metabolism	hair	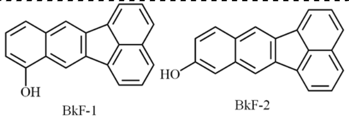	[26]
	Microbial degradation	freshwater microalgae *Selenastrum capricornutum*	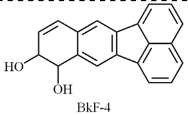	[28]

	Microbial degradation	*Sphingobium sp.* strain KK22 isolated from a soil bacterial consortium that grew on diesel fuel and biodegraded PAHs	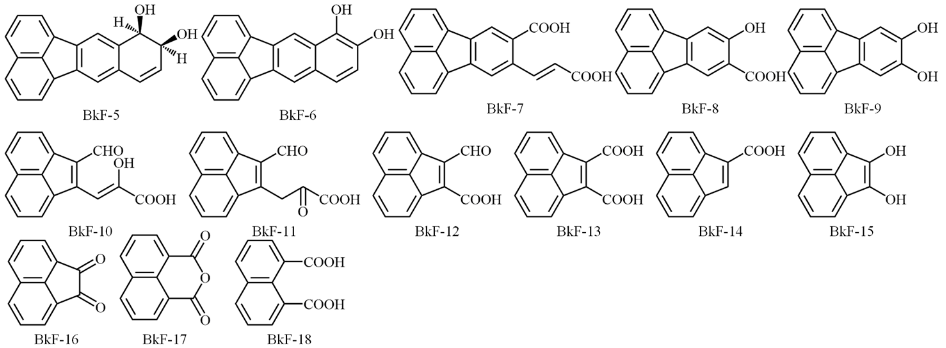	[32]
CHR	Biological metabolism	fish (Atlantic cod)	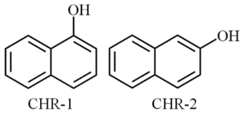	[33]
	Human metabolism	urine	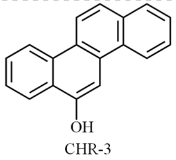	[15]
	Microbial degradation	bacteria	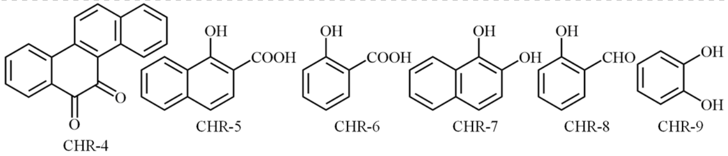	[34][35]
	Microbial degradation	fungus *Fusarium sp.* F092; under saline conditions	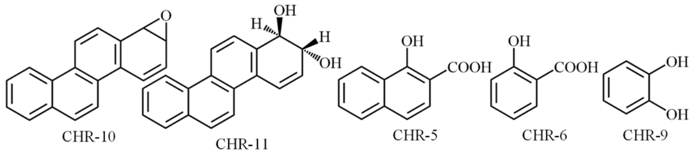	[36]

DahA	Biological metabolism	rat urine	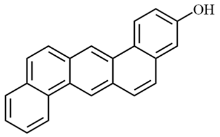 DahA-1	[31]
	Microbial degradation	*Aspergillus terricola*	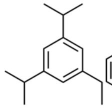 DahA-2 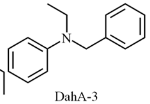 DahA-3 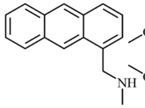 DahA-4 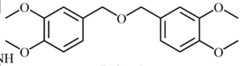 DahA-5 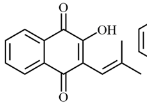 DahA-6 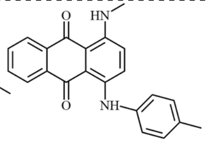 DahA-7	[37]
FLR	Biological metabolism	bovine urine	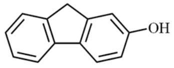 FLR-1 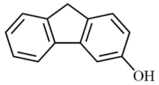 FLR-2 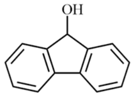 FLR-3	[38]
	Human metabolism	urine	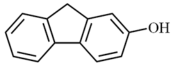 FLR-1 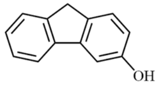 FLR-2 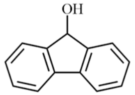 FLR-3	[15]
	Biological metabolism	fish bile	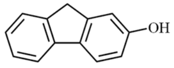 FLR-1 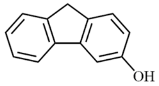 FLR-2 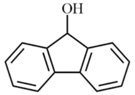 FLR-3	[39]

Microbial degradation	bacteria	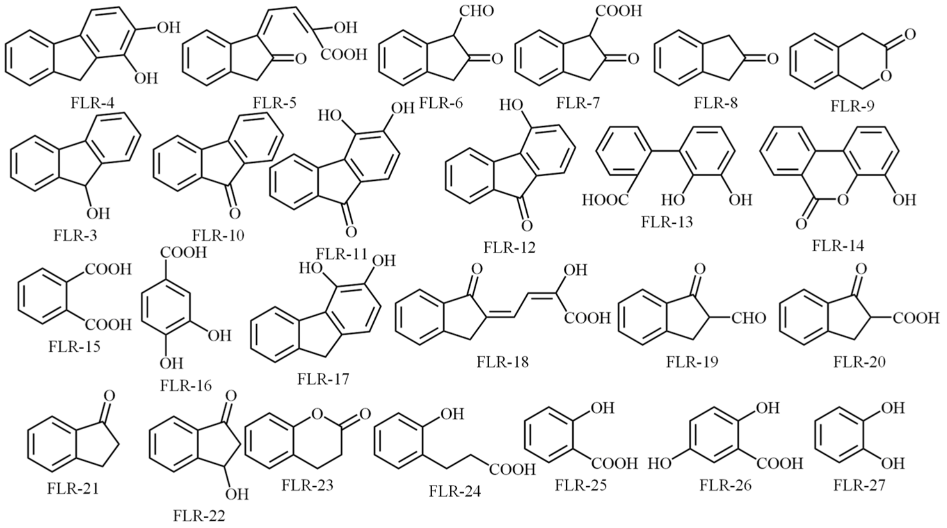	[23]
Anaerobic biodegradation	sulfate-reducing bacteria	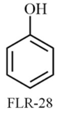	[40]
Microbial degradation	fungi	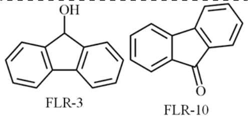	[41]
Microbial degradation	freshwater green alga *Selenastrum capricornutum*	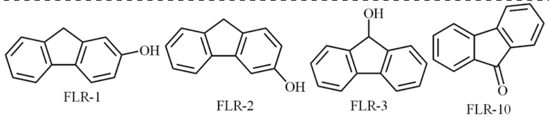	[42]

FRT	Biological metabolism	Common sole (Solea solea)	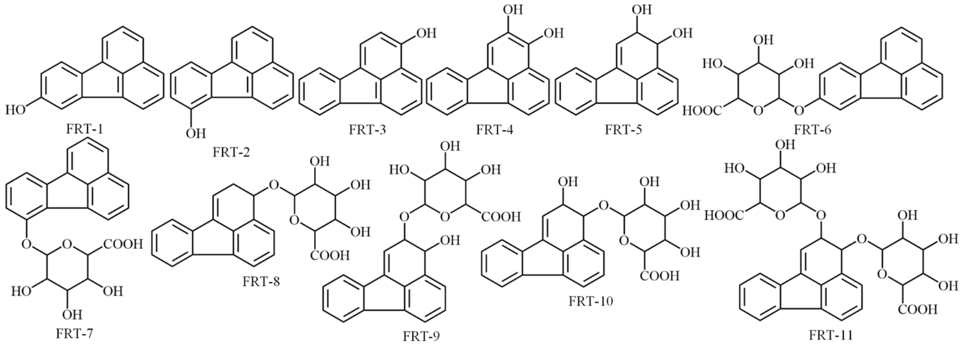	[43]
	Human metabolism	hair	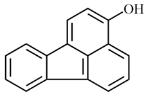 FRT-3	[26]

Microbial degradation	bacteria	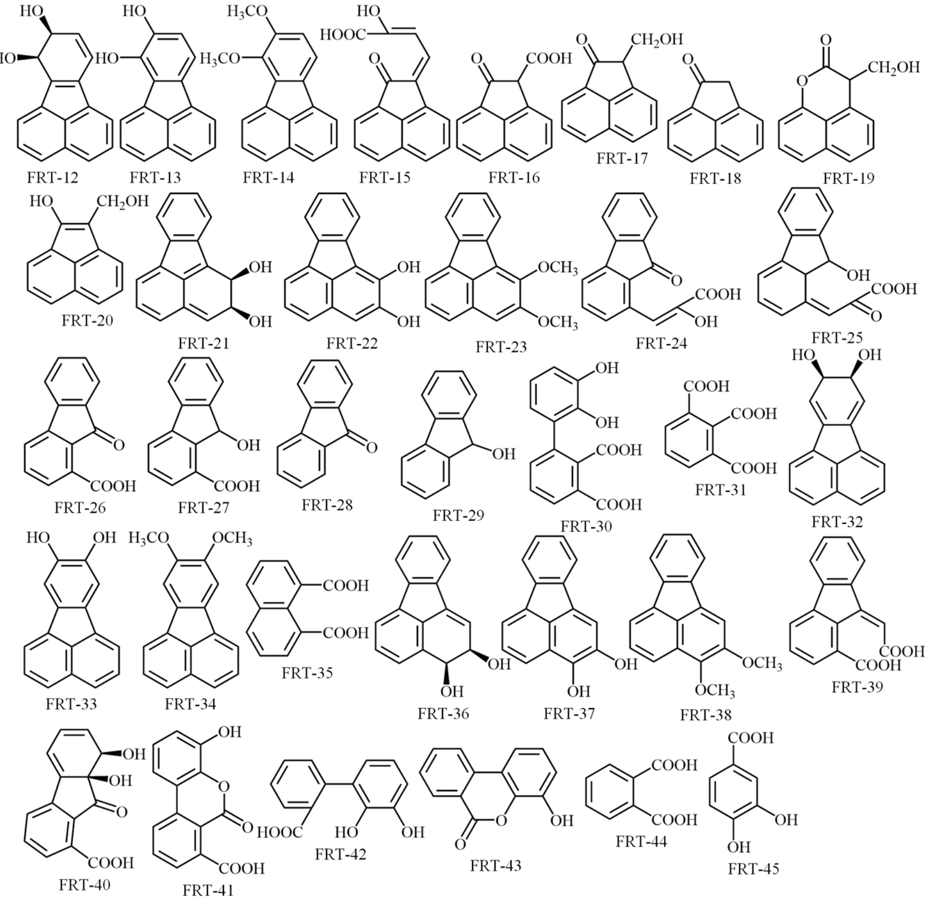	[23][44]

	Microbial degradation	fungi strains from a Brazilian tropical peat	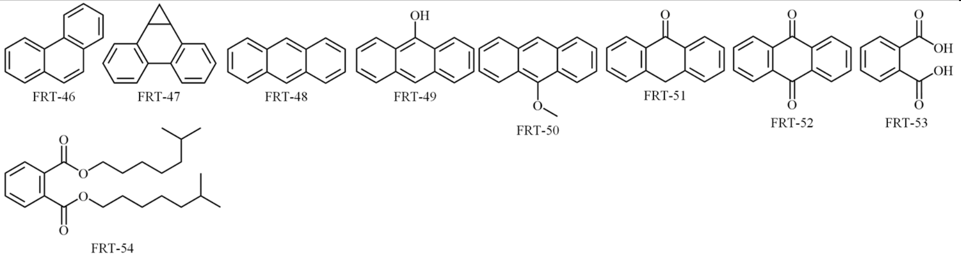	[45]
	Microbial degradation	freshwater microalgae *Selenastrum capricornutum*	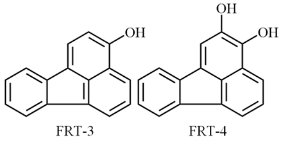	[28][46]
IcdP	Biological metabolism	rat urine	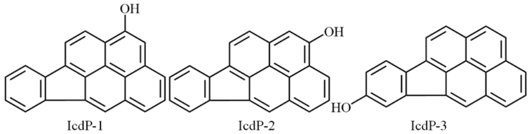	[31]
	Biological metabolism	mouse skin	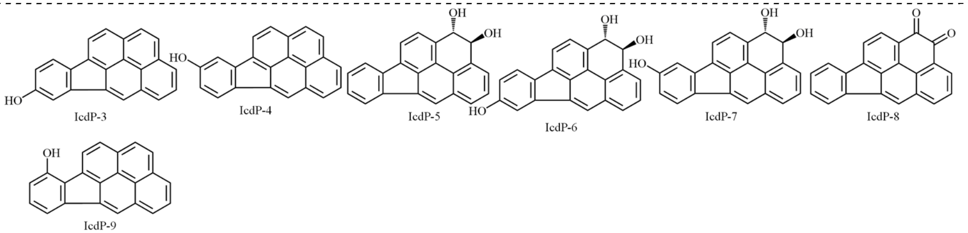	[47]
	Human metabolism	hair	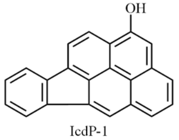	[26]

	Microbial degradation	fungus *Aspergillus terricola*	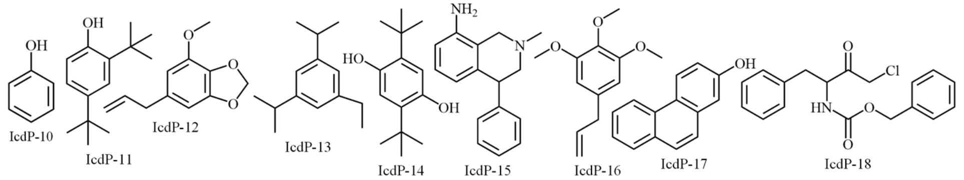	[37]
	Microbial degradation	*Rhodococcus aetherivorans* IcdP1	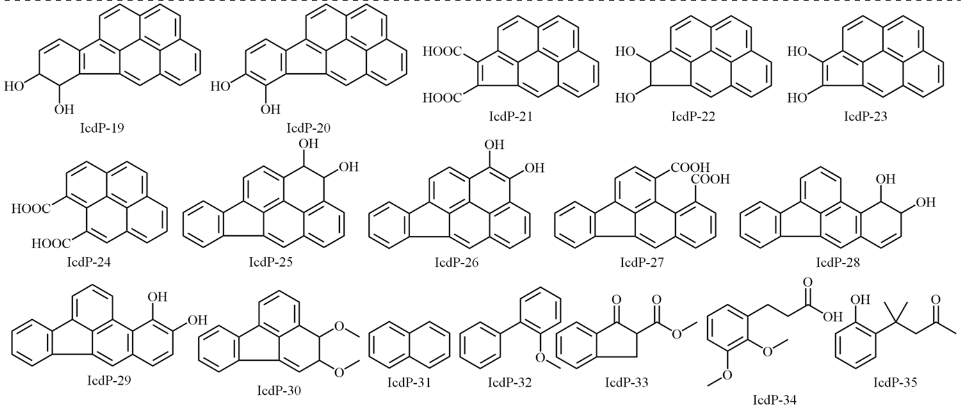	[48]
	Biological metabolism	metabolites from biological materials	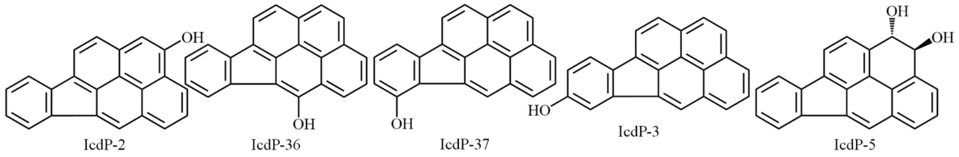	[49]
NAP	Human metabolism	urine	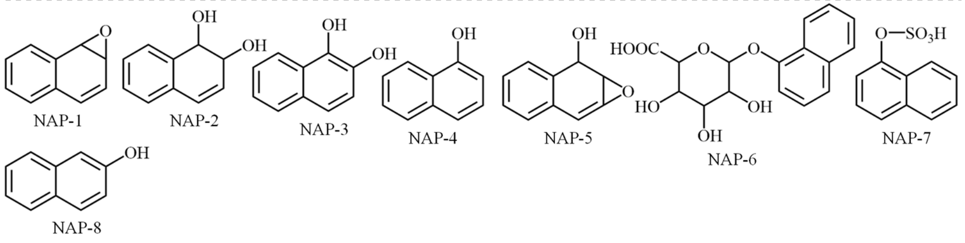	[14][15][50][51]

Human metabolism	liver microsomes	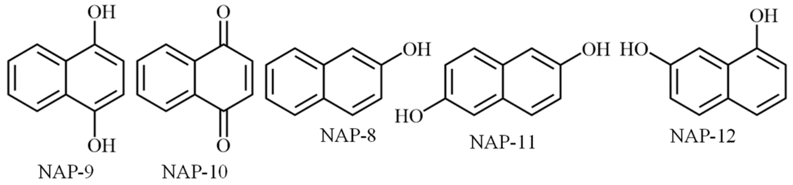 NAP-9, NAP-10, NAP-8, NAP-11, NAP-12	[52]
Biological metabolism	mice (urine)	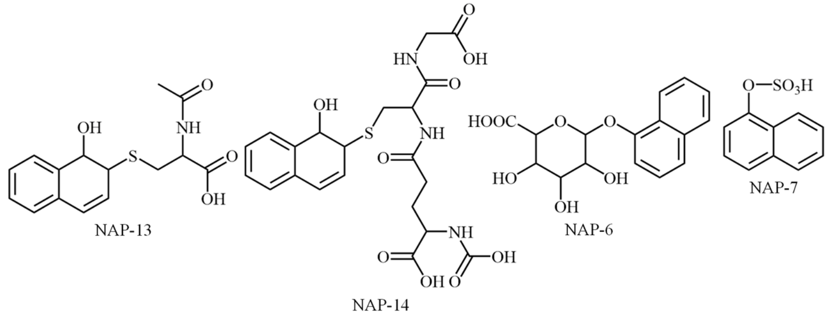 NAP-13, NAP-14, NAP-6, NAP-7	[53]
Biological metabolism	fish (Atlantic cod)	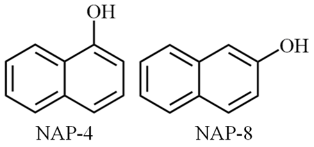 NAP-4, NAP-8	[33]
Biological metabolism	bovine urine	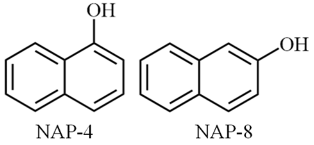 NAP-4, NAP-8	[38]

	Anaerobic biodegradation	bacteria	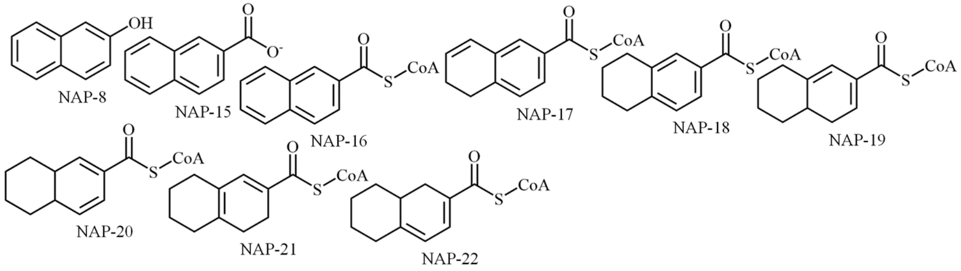	[54][55]
	Microbial degradation	bacteria	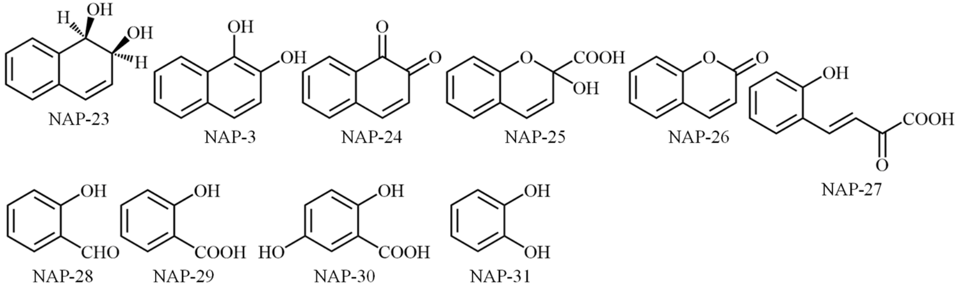	[23]
	Microbial degradation	cyanobacterium *Oscillatoria sp.* JCM	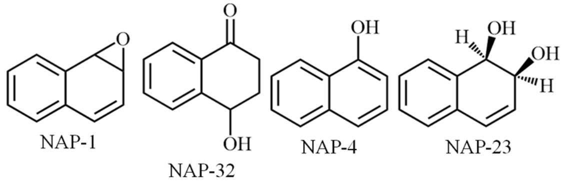	[56]
PHE	Biological metabolism	marine teleosts	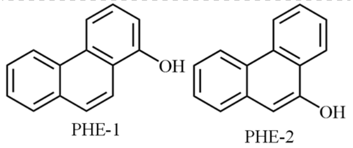	[57]

Biological metabolism	bovine urine	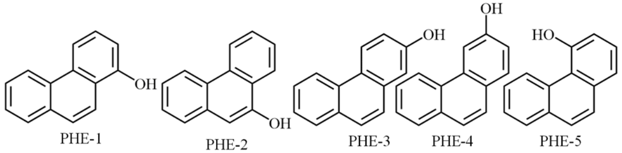	[38]
Human metabolism	urine	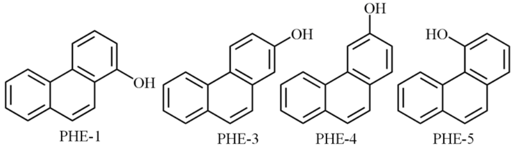	[15][50]
Photolysis	PHE on Fe^{3+}-montmorillonite surface under visible light	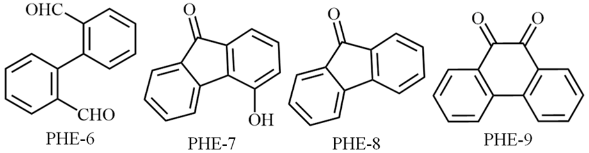	[10]

Microbial degradation	bacteria	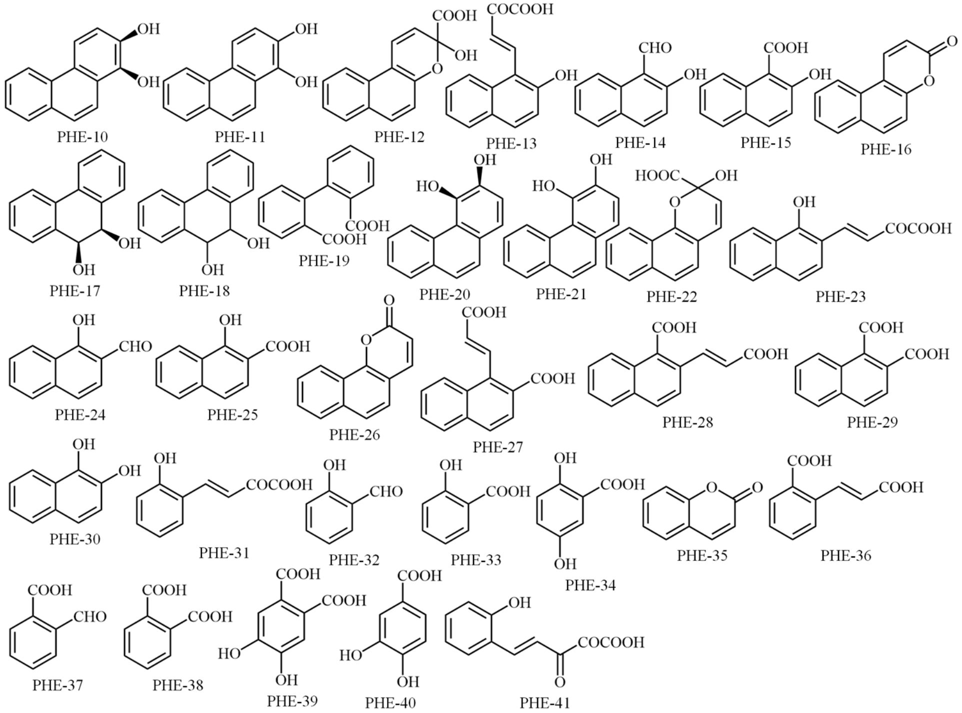	[23]
Coupled photocatalysis and biodegradation	photocatalyst (Mn_3O_4/MnO_2-Ag_3PO_4) and bacteria under visible light irradiation; PHE in water	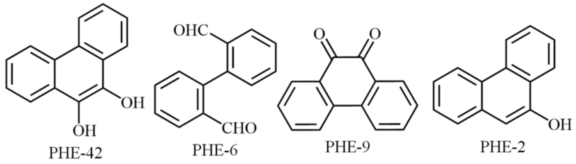	[58]

	Microbial degradation	green alga *Scenedesmus obliquus* ES-55	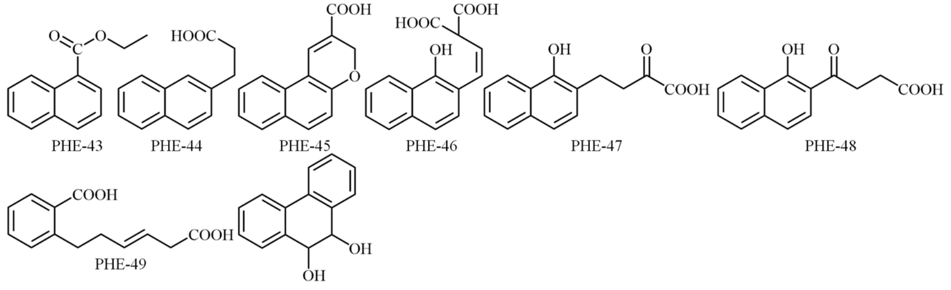 PHE-43, PHE-44, PHE-45, PHE-46, PHE-47, PHE-48, PHE-49, PHE-18	[59]
	Coupled plant and microbial catabolisms	PHE in the rhizosphere of *Medicago sativa*	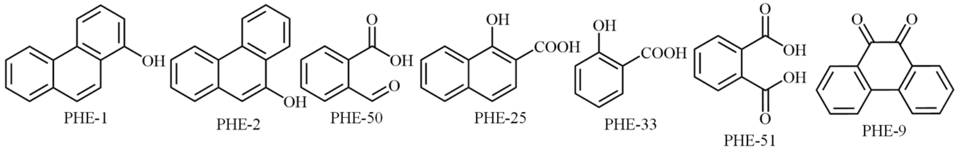 PHE-1, PHE-2, PHE-50, PHE-25, PHE-33, PHE-51, PHE-9	[60]
	Microbial degradation	filamentous fungus *Cyclothyrium sp.* CBS 109850 from estuarine sediment	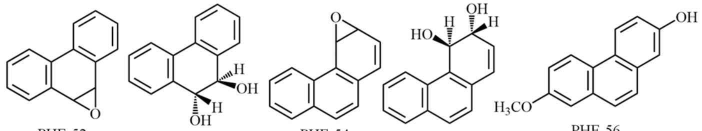 PHE-52, PHE-53, PHE-54, PHE-55, PHE-56	[5]
PYR	Biological metabolism	bovine urine	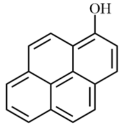 PYR-1	[38]
	Biological metabolism	bile of flounder	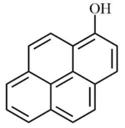 PYR-1	[16][61]

Human metabolism	urine	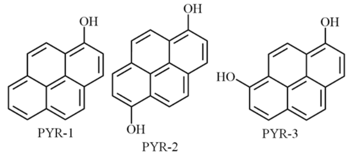	[14][15][50]
Resistance of plants	rooted poplar cuttings	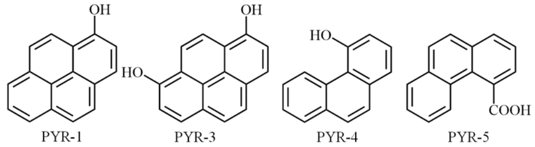	[19]
Microbial degradation	filamentous fungus *Cyclothyrium sp.* CBS 109850 from estuarine sediment	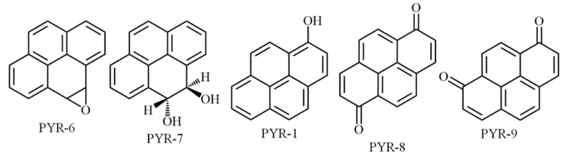	[5]

Microbial degradation	bacteria	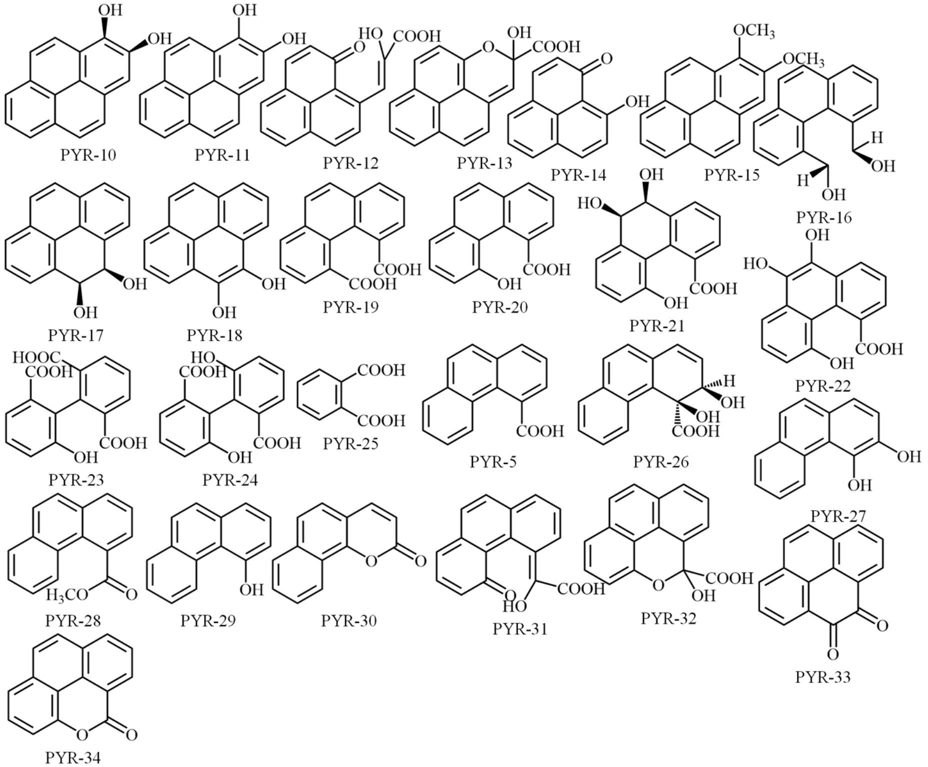	[23]

Photocatalytic degradation	in presence of goethite under UV irradiation	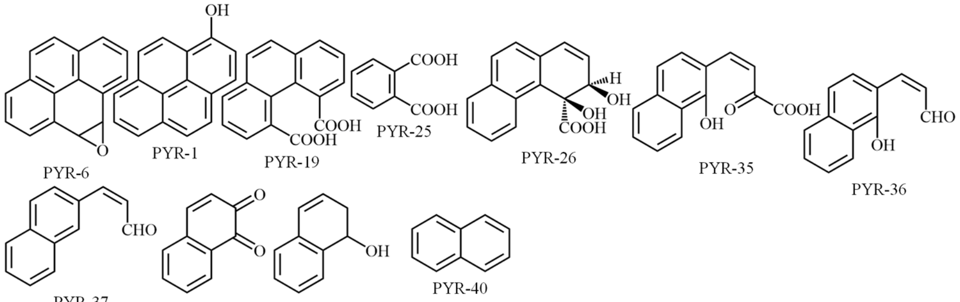 PYR-6 PYR-1 PYR-19 PYR-25 PYR-26 PYR-35 PYR-36 PYR-37 PYR-38 PYR-39 PYR-40	[62]
Microbial degradation	freshwater green alga *Selenastrum capricornutum*	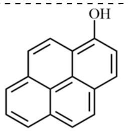 PYR-1	[42]

Table S2 Binding free energy for PAHs' neurotoxicity, immunotoxicity and phytotoxicity

PAHs	Neurotoxicity (binding free energy, kJ/mol)	Immunotoxicity (binding free energy, kJ/mol)	Phytotoxicity (binding free energy, kJ/mol)
1MNAP	-43.422	-61.808	-37.273
2MNAP	-46.392	-47.673	-42.014
ACE	-32.347	-68.893	-54.961
ACY	-46.548	-53.244	-53.263
ANT	-43.915	-69.617	-64.852
BaAN	-55.501	-91.938	-106.834
BANN	-60.793	-88.463	-97.418
BaP	-70.481	-97.864	-103.536
BbF	-44.365	-103.742	-100.331
BeP	-67.026	-92.637	-74.155
BghiP	-64.460	-121.288	-98.467
BkF	-50.842	-112.373	-81.132
CHR	-66.290	-83.232	-84.175
CPPHN	-62.240	-78.146	-81.077
CRN	-83.141	-113.449	-86.995
DahA	-66.480	-105.255	-81.625
FLR	-41.383	-67.423	-83.469
FRT	-49.042	-104.230	-76.780
IcdP	-65.071	-82.398	-108.142
NAP	-26.336	-33.803	-51.637
PHE	-34.993	-54.440	-64.850
PRL	-52.750	-101.852	-93.434
PYR	-48.667	-92.176	-89.548

Table S3 Environmental and human health risk assessment of PAH derivatives

PAH and its derivatives	Neurotoxicity		Immunotoxicity		Phytotoxicity		Developmental toxicity				Genotoxicity				Carcinogenicity		Endocrine disrupting effect	
	Binding free energy (kJ/mol)	Toxicity reduction intensity	Binding free energy (kJ/mol)	Toxicity reduction intensity	Binding free energy (kJ/mol)	Toxicity reduction intensity	Developmental toxicity potential	Toxicity reduction range	Within OPS	Within OPS limit	Ames mutagenicity	Toxicity reduction range	Within OPS	Within OPS limit	Carcinogenicity model (CAESAR)	Assessment reliability	Estrogen receptor relative binding affinity (IRFMN)	Assessment reliability
**ACE**	-32.347	0.00	-68.893	0.00	-54.961	0.00	1.000	0.00	True	True	0.120	0.00	True	True	non-carcinogen	low reliability	inactive	good reliability
ACE-1	-30.863	4.59	-72.418	-5.12	-65.769	-19.66	0.702	0.30	True	True	0.999	-0.88	True	True	non-carcinogen	moderate reliability	inactive	moderate reliability
ACE-2	-32.084	0.81	-65.606	4.77	-75.162	-36.76	0.607	0.39	True	True	0.978	-0.86	True	True	non-carcinogen	moderate reliability	inactive	low reliability
ACE-3	-30.240	6.51	-55.126	19.98	-72.417	-31.76	0.607	0.39	True	True	0.978	-0.86	True	True	non-carcinogen	moderate reliability	inactive	low reliability
ACE-4	-29.815	7.83	-55.532	19.39	-71.965	-30.94	0.607	0.39	True	True	0.978	-0.86	True	True	non-carcinogen	moderate reliability	inactive	low reliability
ACE-5	-28.381	12.26	-59.478	13.67	-66.710	-21.38	0.996	0.00	True	True	1.000	-0.88	True	True	non-carcinogen	moderate reliability	inactive	moderate reliability
ACE-6	-29.573	8.58	-54.318	21.16	-75.818	-37.95	0.182	0.82	True	True	1.000	-0.88	True	True	non-carcinogen	moderate reliability	inactive	moderate reliability
ACE-7	-28.417	12.15	-48.226	30.00	-93.110	-69.41	0.089	0.91	True	True	1.000	-0.88	True	True	non-carcinogen	moderate reliability	inactive	moderate reliability
ACE-8	-34.624	-7.04	-53.438	22.43	-88.114	-60.32	0.067	0.93	True	True	0.000	0.12	False	False	non-carcinogen	good reliability	inactive	good reliability
ACE-9	-35.297	-9.12	-69.253	-0.52	-94.274	-71.53	0.077	0.92	True	True	1.000	-0.88	True	True	carcinogen	low reliability	inactive	moderate reliability

ACE-10	-32.985	-1.97	-63.356	8.04	-59.313	-7.92	0.005	1.00	True	True	0.204	-0.08	True	True	non-carcinogen	low reliability	inactive	good reliability
ACE-11	-35.505	-9.76	-33.795	50.95	-85.644	-55.83	0.016	0.98	True	True	0.000	0.12	False	False	carcinogen	moderate reliability	inactive	low reliability
ACE-12	-38.279	-18.34	-14.795	78.52	-95.571	-73.89	0.016	0.98	True	True	0.000	0.12	False	False	carcinogen	low reliability	inactive	low reliability
ACE-13	-36.994	-14.37	-41.833	39.28	-97.225	-76.90	0.012	0.99	True	True	1.000	-0.88	False	False	non-carcinogen	moderate reliability	active	moderate reliability
ACE-14	-49.724	-53.72	-24.815	63.98	-123.595	-124.88	0.008	0.99	True	True	0.000	0.12	True	True	non-carcinogen	good reliability	inactive	good reliability
ACE-15	-43.768	-35.31	-19.993	70.98	-119.667	-117.73	0.017	0.98	True	True	0.000	0.12	True	True	carcinogen	low reliability	inactive	good reliability
ACE-16	-26.776	17.22	-39.617	42.49	-99.386	-80.83	0.001	1.00	True	True	0.000	0.12	True	True	non-carcinogen	low reliability	inactive	good reliability
ACE-17	-29.119	9.98	-29.651	56.96	-80.222	-45.96	0.013	0.99	True	True	0.000	0.12	True	True	carcinogen	experimental value	inactive	experimental value
**ACY**	-46.548	0.00	-53.244	0.00	-53.263	0.00	0.998	0.00	False	False	0.515	0.00	True	True	non-carcinogen	low reliability	inactive	good reliability
ACY-1	-32.176	30.88	-68.571	-28.79	-75.517	-41.78	0.607	0.39	True	True	0.978	-0.46	True	True	non-carcinogen	moderate reliability	inactive	low reliability
ACY-2	-29.574	36.47	-54.325	-2.03	-75.821	-42.35	0.182	0.82	True	True	1.000	-0.49	True	True	non-carcinogen	moderate reliability	inactive	moderate reliability
ACY-3	-23.973	48.50	-34.771	34.69	-55.831	-4.82	0.089	0.91	True	True	0.995	-0.48	True	True	non-carcinogen	moderate reliability	inactive	low reliability
ACY-4	-28.417	38.95	-48.226	9.42	-93.110	-74.81	0.089	0.91	True	True	1.000	-0.49	True	True	non-carcinogen	moderate reliability	inactive	moderate reliability

ACY-5	-31.333	32.69	-58.978	-10.77	-98.245	-84.45	0.067	0.93	True	True	0.000	0.52	False	False	non-carcinogen	good reliability	inactive	good reliability
ACY-6	-32.986	29.14	-63.365	-19.01	-59.312	-11.36	0.005	0.99	True	True	0.204	0.31	True	True	non-carcinogen	low reliability	inactive	good reliability
ACY-7	-36.556	21.47	-31.346	41.13	-66.768	-25.36	0.001	1.00	True	True	0.000	0.52	True	True	non-carcinogen	low reliability	inactive	good reliability
ACY-8	-35.979	22.71	-32.258	39.41	-90.187	-69.32	0.006	0.99	True	True	0.000	0.52	True	True	non-carcinogen	good reliability	inactive	good reliability
ACY-9	-28.381	39.03	-59.479	-11.71	-66.710	-25.25	0.996	0.00	True	True	1.000	-0.49	True	True	non-carcinogen	moderate reliability	inactive	moderate reliability
ACY-10	-14.249	69.39	-72.988	-37.08	-122.813	-130.58	0.024	0.97	True	True	0.000	0.52	False	False	non-carcinogen	good reliability	inactive	good reliability
**ANT**	-43.915	0.00	-69.617	0.00	-64.852	0.00	0.535	0.00	True	True	0.000	0.00	True	True	non-carcinogen	low reliability	inactive	low reliability
ANT-1	-38.285	12.82	-87.040	-25.03	-87.551	-35.00	0.359	0.18	True	True	0.926	-0.93	True	True	non-carcinogen	low reliability	inactive	moderate reliability
ANT-2	-42.833	2.46	-93.540	-34.36	-101.756	-56.90	0.966	-0.43	True	True	0.000	0.00	True	True	non-carcinogen	low reliability	inactive	moderate reliability
ANT-3	-34.555	21.31	-80.937	-16.26	-95.789	-47.70	0.383	0.15	True	True	0.000	0.00	True	True	carcinogen	low reliability	inactive	low reliability
ANT-4	-41.731	4.97	-61.164	12.14	-69.193	-6.69	0.008	0.53	True	True	1.000	-1.00	True	True	carcinogen	moderate reliability	inactive	moderate reliability
ANT-5	-36.587	16.69	-49.685	28.63	-27.961	56.88	0.373	0.16	True	True	1.000	-1.00	True	True	carcinogen	low reliability	inactive	low reliability
ANT-6	-51.064	-16.28	-96.738	-38.96	-113.856	-75.56	0.383	0.15	True	True	0.001	0.00	True	True	carcinogen	good reliability	inactive	moderate reliability

ANT-7	-28.192	35.80	-59.602	14.39	-107.636	-65.97	0.004	0.53	True	True	0.000	0.00	True	True	non-carcinogen	experimental value	inactive	good reliability
ANT-8	-41.449	5.62	-58.364	16.16	-93.131	-43.61	0.002	0.53	True	True	0.000	0.00	True	True	non-carcinogen	moderate reliability	inactive	good reliability
ANT-9	-43.261	1.49	-56.635	18.65	-108.081	-66.66	0.003	0.53	True	True	0.000	0.00	True	True	non-carcinogen	good reliability	inactive	good reliability
ANT-10	-35.540	19.07	-40.818	41.37	-76.543	-18.03	0.001	0.53	True	True	0.000	0.00	True	True	non-carcinogen	experimental value	inactive	good reliability
ANT-11	-29.119	33.69	-29.651	57.41	-80.222	-23.70	0.013	0.52	True	True	0.000	0.00	True	True	carcinogen	experimental value	inactive	experimental value
ANT-12	-48.540	-10.53	-94.515	-35.76	-102.802	-58.52	0.998	-0.46	True	True	0.000	0.00	True	True	carcinogen	good reliability	inactive	moderate reliability
ANT-13	-33.553	23.60	-43.133	38.04	-70.380	-8.52	0.994	-0.46	True	True	0.001	0.00	True	True	non-carcinogen	low reliability	inactive	moderate reliability
ANT-14	-44.724	-1.84	-71.714	-3.01	-55.623	14.23	1.000	-0.47	True	True	1.000	-1.00	False	False	carcinogen	moderate reliability	active	experimental value
ANT-15	-40.467	7.85	-59.038	15.20	-95.017	-46.51	0.277	0.26	True	True	1.000	-1.00	False	False	non-carcinogen	low reliability	inactive	low reliability
ANT-16	-25.755	41.35	-54.026	22.40	-92.852	-43.18	0.012	0.52	True	True	0.000	0.00	True	True	non-carcinogen	experimental value	inactive	good reliability
ANT-17	-25.304	42.38	-39.537	43.21	-83.491	-28.74	0.004	0.53	True	True	0.000	0.00	True	True	non-carcinogen	experimental value	inactive	experimental value
ANT-18	-11.525	73.76	-56.284	19.15	-61.639	4.95	1.000	-0.47	True	True	0.000	0.00	False	False	non-carcinogen	good reliability	inactive	low reliability
ANT-19	-49.860	-13.54	-75.335	-8.21	-62.018	4.37	0.150	0.39	True	True	1.000	-1.00	True	True	carcinogen	low reliability	inactive	low reliability

ANT-20	-26.578	39.48	-33.558	51.80	-51.347	20.82	0.154	0.38	True	True	0.000	0.00	True	True	non-carcinogen	low reliability	inactive	low reliability
ANT-21	-55.954	-27.41	-40.603	41.68	-96.134	-48.24	0.375	0.16	True	True	1.000	-1.00	False	False	non-carcinogen	low reliability	inactive	low reliability
ANT-22	-19.000	56.73	-53.296	23.44	-120.143	-85.26	0.000	0.54	True	True	0.912	-0.91	True	True	carcinogen	moderate reliability	inactive	moderate reliability
**BaAN**	-55.501	0.00	-91.938	0.00	-106.834	0.00	0.497	0.00	True	True	1.000	0.000	True	True	carcinogen	low reliability	active	experimental value
BaAN-1	-40.439	27.14	-73.763	19.77	-74.866	29.92	0.953	-0.46	True	True	1.000	0.000	True	True	carcinogen	low reliability	inactive	moderate reliability
BaAN-2	-40.450	27.12	-88.804	3.41	-81.766	23.46	0.961	-0.46	True	True	1.000	0.000	True	True	carcinogen	low reliability	inactive	low reliability
BaAN-3	-31.672	42.93	-50.786	44.76	-60.959	42.94	0.959	-0.46	True	True	1.000	0.000	True	True	carcinogen	low reliability	inactive	low reliability
BaAN-4	-34.954	37.02	-83.514	9.16	-80.376	24.77	0.962	-0.47	True	True	1.000	0.000	True	True	carcinogen	low reliability	inactive	low reliability
BaAN-5	-51.011	8.09	-84.634	7.94	-59.942	43.89	0.960	-0.46	True	True	1.000	0.000	True	True	carcinogen	low reliability	inactive	low reliability
BaAN-6	-39.325	29.15	-88.986	3.21	-88.281	17.37	0.970	-0.47	True	True	1.000	0.000	True	True	non-carcinogen	low reliability	inactive	low reliability
BaAN-7	-29.036	47.68	-48.838	46.88	-58.054	45.66	0.969	-0.47	True	True	1.000	0.000	True	True	non-carcinogen	low reliability	inactive	low reliability
BaAN-8	-35.145	36.68	-60.729	33.95	-59.944	43.89	0.970	-0.47	True	True	1.000	0.000	True	True	non-carcinogen	low reliability	inactive	low reliability
BaAN-9	-30.055	45.85	-42.767	53.48	-55.540	48.01	0.969	-0.47	True	True	1.000	0.000	True	True	non-carcinogen	low reliability	inactive	low reliability

BaAN-10	-41.716	24.84	-84.647	7.93	-78.958	26.09	1.000	-0.50	True	True	1.000	0.000	True	True	non-carcinogen	low reliability	inactive	low reliability
BaAN-11	-30.722	44.65	-49.536	46.12	-59.444	44.36	1.000	-0.50	True	True	1.000	0.000	True	True	non-carcinogen	low reliability	inactive	low reliability
BaAN-12	-55.764	-0.47	-115.276	-25.38	-117.772	-10.24	0.473	0.02	True	True	1.000	0.000	True	True	carcinogen	good reliability	inactive	moderate reliability
BaAN-13	-34.754	37.38	-52.225	43.20	-64.024	40.07	0.089	0.41	True	True	0.900	0.100	True	True	carcinogen	low reliability	inactive	low reliability
BaAN-14	-43.949	20.81	-69.376	24.54	-64.851	39.30	0.535	-0.04	True	True	0.000	1.000	True	True	non-carcinogen	low reliability	inactive	low reliability
BaAN-15	-48.064	13.40	-77.284	15.94	-40.669	61.93	0.000	0.50	False	False	1.000	0.000	False	False	carcinogen	low reliability	active	low reliability
BaAN-16	-14.788	73.36	-77.176	16.06	-123.041	-15.17	0.024	0.47	True	True	0.000	1.000	False	False	non-carcinogen	good reliability	inactive	good reliability
BaAN-17	-41.313	25.56	-57.289	37.69	-75.832	29.02	1.000	-0.50	True	True	0.986	0.014	True	True	non-carcinogen	moderate reliability	inactive	moderate reliability
BaAN-18	-28.017	49.52	-57.232	37.75	-68.650	35.74	0.973	-0.48	True	True	0.004	0.996	True	True	carcinogen	moderate reliability	inactive	moderate reliability
BaAN-19	-45.092	18.75	-60.944	33.71	-91.279	14.56	0.003	0.49	True	True	1.000	0.000	True	True	non-carcinogen	low reliability	inactive	moderate reliability
BaAN-20	-50.880	8.33	-50.418	45.16	-97.540	8.70	0.129	0.37	True	True	0.284	0.716	True	True	carcinogen	low reliability	inactive	moderate reliability
BaAN-21	-41.984	24.35	-61.800	32.78	-86.123	19.39	0.228	0.27	True	True	0.732	0.268	True	True	non-carcinogen	moderate reliability	inactive	good reliability
BaAN-22	-49.463	10.88	-43.963	52.18	-81.706	23.52	0.003	0.49	True	True	0.000	1.000	True	True	non-carcinogen	good reliability	inactive	good reliability

BaAN-23	-48.281	13.01	-36.989	59.77	-69.292	35.14	0.001	0.50	True	True	0.000	1.000	True	True	non-carcinogen	low reliability	inactive	experimental value
BaAN-24	-67.321	-21.30	-62.711	31.79	-84.318	21.08	0.002	0.50	True	True	0.000	1.000	True	True	non-carcinogen	low reliability	inactive	experimental value
BaAN-25	-27.605	50.26	-25.966	71.76	-60.790	43.10	0.950	-0.45	True	True	0.000	1.000	True	True	non-carcinogen	moderate reliability	inactive	good reliability
BaAN-26	-44.830	19.23	-98.335	-6.96	-82.113	23.14	0.864	-0.37	True	True	1.000	0.000	True	True	carcinogen	good reliability	active	moderate reliability
BaAN-27	-47.442	14.52	-88.478	3.76	-68.091	36.26	0.800	-0.30	True	True	1.000	0.000	True	True	non-carcinogen	low reliability	active	moderate reliability
BaAN-28	-25.556	53.95	-67.314	26.78	-143.955	-34.75	0.067	0.43	True	True	0.000	1.000	False	False	non-carcinogen	low reliability	inactive	good reliability
BaAN-29	-48.481	12.65	-44.087	52.05	-110.893	-3.80	0.004	0.49	True	True	0.960	0.040	True	True	non-carcinogen	moderate reliability	inactive	low reliability
BaAN-30	-42.803	22.88	-51.576	43.90	-76.311	28.57	0.001	0.50	True	True	0.000	1.000	True	True	non-carcinogen	low reliability	inactive	good reliability
**BaP**	-58.732	0.00	-84.834	0.00	-78.441	0.00	0.976	0.00	True	True	1.000	0.000	True	True	carcinogen	experimental value	inactive	experimental value
BaP-1	-58.469	0.45	-101.226	-19.32	-48.984	37.55	0.996	-0.02	True	True	1.000	0.000	True	True	carcinogen	low reliability	active	low reliability
BaP-2	-66.438	-13.12	-45.446	46.43	-88.485	-12.80	0.999	-0.02	True	True	1.000	0.000	True	True	carcinogen	moderate reliability	active	low reliability
BaP-3	-34.911	40.56	-80.894	4.64	-118.813	-51.47	0.848	0.13	True	True	1.000	0.000	True	True	carcinogen	moderate reliability	inactive	good reliability
BaP-4	-38.232	34.90	-26.098	69.24	-96.905	-23.54	0.031	0.95	True	True	0.002	0.998	True	True	carcinogen	low reliability	inactive	moderate reliability

BaP-5	-23.614	59.79	-33.312	60.73	-53.228	32.14	0.999	-0.02	False	True	0.000	1.000	False	False	non-carcinogen	low reliability	inactive	low reliability
BaP-6	-65.710	-11.88	-97.378	-14.79	-95.205	-21.37	1.000	-0.02	False	False	0.000	1.000	True	True	non-carcinogen	low reliability	active	moderate reliability
BaP-7	-58.996	-0.45	-76.053	10.35	-107.645	-37.23	0.029	0.95	True	True	0.000	1.000	True	True	non-carcinogen	low reliability	active	experimental value
BaP-8	-69.026	-17.53	-84.638	0.23	-95.548	-21.81	0.086	0.89	True	True	0.000	1.000	True	True	non-carcinogen	low reliability	inactive	low reliability
BaP-9	-43.269	26.33	-59.679	29.65	-82.348	-4.98	0.002	0.97	True	True	0.202	0.798	True	True	non-carcinogen	low reliability	inactive	low reliability
BaP-10	-23.911	59.29	-41.319	51.29	-70.455	10.18	0.502	0.47	True	True	0.000	1.000	True	True	carcinogen	experimental value	inactive	experimental value
BaP-11	-29.818	49.23	-63.633	24.99	-58.147	25.87	0.000	0.98	False	False	0.999	0.001	True	True	non-carcinogen	low reliability	inactive	moderate reliability
BaP-12	-26.231	55.34	-48.729	42.56	-74.161	5.46	0.506	0.47	True	True	0.000	1.000	True	True	carcinogen	good reliability	inactive	good reliability
BaP-13	-26.034	55.67	-40.327	52.46	-68.092	13.19	0.000	0.98	True	True	0.000	1.000	True	True	carcinogen	good reliability	inactive	good reliability
BaP-14	-26.232	55.34	-48.731	42.56	-74.165	5.45	0.506	0.47	True	True	0.000	1.000	True	True	carcinogen	good reliability	inactive	good reliability
BaP-15	-39.672	32.45	-71.885	15.26	-64.167	18.20	0.000	0.98	False	False	0.999	0.001	True	True	carcinogen	moderate reliability	active	low reliability
BaP-16	-44.207	24.73	-56.523	33.37	-80.926	-3.17	1.000	-0.02	True	True	1.000	0.000	False	False	non-carcinogen	low reliability	inactive	low reliability
BaP-17	-76.282	-29.88	-115.530	-36.18	-138.780	-76.92	0.998	-0.02	True	True	1.000	0.000	True	True	non-carcinogen	low reliability	inactive	moderate reliability

BaP-18	-78.005	-32.82	-97.775	-15.25	-96.232	-22.68	1.000	-0.02	True	True	1.000	0.000	True	True	non-carcinogen	low reliability	inactive	low reliability
BaP-19	-84.016	-43.05	-83.057	2.09	-50.890	35.12	1.000	-0.02	True	True	0.004	0.996	True	True	carcinogen	low reliability	active	good reliability
BaP-20	-39.068	33.48	-53.420	37.03	-32.779	58.21	—[b]	—	—	—	—	—	—	—	carcinogen	moderate reliability	active	low reliability
BaP-21	-61.761	-5.16	-76.041	10.36	-89.542	-14.15	0.966	0.01	True	True	0.023	0.977	True	True	carcinogen	good reliability	inactive	low reliability
BaP-22	-38.847	33.86	-58.981	30.47	-98.691	-25.82	0.974	0.00	True	True	1.000	0.000	True	True	carcinogen	low reliability	inactive	good reliability
BaP-23	-74.107	-26.18	-125.188	-47.57	-94.230	-20.13	1.000	-0.02	True	True	1.000	0.000	True	True	carcinogen	moderate reliability	active	moderate reliability
BaP-24	-50.819	13.47	-82.240	3.06	-114.134	-45.50	0.976	0.00	True	True	1.000	0.000	True	True	carcinogen	low reliability	inactive	moderate reliability
BaP-25	-58.142	1.00	-113.749	-34.08	-63.037	19.64	0.996	-0.02	True	True	1.000	0.000	True	True	carcinogen	low reliability	active	low reliability
BaP-26	-41.752	28.91	-80.297	5.35	-82.505	-5.18	0.976	0.00	True	True	1.000	0.000	True	True	carcinogen	low reliability	inactive	low reliability
BaP-27	-69.585	-18.48	-72.242	14.84	-72.477	7.60	—	—	—	—	—	—	—	—	carcinogen	low reliability	active	moderate reliability
BaP-28	-34.162	41.83	-54.486	35.77	-55.854	28.79	0.999	-0.02	True	True	1.000	0.000	True	True	carcinogen	low reliability	active	good reliability
BaP-29	-50.021	14.83	-83.869	1.14	-53.166	32.22	0.996	-0.02	True	True	1.000	0.000	True	True	carcinogen	low reliability	active	low reliability
BaP-30	-53.336	9.19	-105.899	-24.83	-46.346	40.92	0.999	-0.02	True	True	1.000	0.000	True	True	carcinogen	low reliability	active	good reliability

BaP-31	-40.143	31.65	-63.363	25.31	-50.450	35.68	0.999	-0.02	True	True	1.000	0.000	True	True	carcinogen	low reliability	active	good reliability
BaP-32	-69.429	-18.21	-52.613	37.98	-80.492	-2.61	0.999	-0.02	True	True	1.000	0.000	True	True	carcinogen	moderate reliability	active	low reliability
BaP-33	-37.586	36.00	-61.317	27.72	-49.396	37.03	0.091	0.89	True	True	1.000	0.000	True	True	carcinogen	low reliability	inactive	moderate reliability
BaP-34	-34.755	40.82	-52.226	38.44	-64.023	18.38	0.089	0.89	True	True	0.900	0.100	True	True	carcinogen	low reliability	inactive	low reliability
BaP-35	-38.074	35.17	-62.904	25.85	-64.379	17.93	0.150	0.83	True	True	1.000	0.000	True	True	carcinogen	low reliability	inactive	low reliability
BaP-36	-35.196	40.07	-55.812	34.21	-46.446	40.79	0.034	0.94	True	True	0.999	0.001	True	True	non-carcinogen	low reliability	inactive	good reliability
BaP-37	-32.115	45.32	-38.321	54.83	-49.376	37.05	0.011	0.97	True	True	1.000	0.000	True	True	carcinogen	moderate reliability	inactive	low reliability
BaP-38	-32.072	45.39	-38.857	54.20	-47.681	39.21	0.006	0.97	True	True	0.992	0.008	True	True	non-carcinogen	low reliability	inactive	good reliability
BaP-39	-38.214	34.93	-26.064	69.28	-96.907	-23.54	0.031	0.95	True	True	0.002	0.998	True	True	carcinogen	low reliability	inactive	moderate reliability
BaP-40	-68.631	-16.85	-73.949	12.83	-89.310	-13.86	0.007	0.97	True	True	0.000	1.000	True	True	non-carcinogen	good reliability	inactive	experimental value
BaP-41	-65.836	-12.10	-67.899	19.96	-95.431	-21.66	0.025	0.95	True	True	0.064	0.936	True	True	non-carcinogen	good reliability	inactive	good reliability
BaP-42	-36.489	37.87	-73.558	13.29	-70.976	9.52	0.051	0.93	True	True	0.986	0.014	True	True	carcinogen	moderate reliability	inactive	low reliability
BaP-43	-49.087	16.42	-78.391	7.59	-102.340	-30.47	0.098	0.88	True	True	0.000	1.000	False	True	non-carcinogen	low reliability	inactive	good reliability

BaP-44	-50.138	14.63	-74.334	12.38	-91.234	-16.31	0.974	0.00	True	True	0.330	0.670	True	True	non-carcinogen	good reliability	inactive	good reliability
BaP-45	-27.294	53.53	-14.879	82.46	-71.301	9.10	0.882	0.09	True	True	0.660	0.340	False	False	non-carcinogen	low reliability	inactive	low reliability
BaP-46	-52.706	10.26	-52.083	38.61	-79.359	-1.17	0.025	0.95	True	True	0.994	0.006	True	True	non-carcinogen	low reliability	inactive	moderate reliability
BaP-47	-33.014	43.79	-54.494	35.76	-63.761	18.71	0.025	0.95	True	True	0.999	0.001	True	True	carcinogen	moderate reliability	inactive	moderate reliability
BaP-48	-42.028	28.44	-73.513	13.34	-95.596	-21.87	0.006	0.97	True	True	0.979	0.021	True	True	non-carcinogen	moderate reliability	active	experimental value
BaP-49	-30.391	48.25	-41.394	51.21	-57.966	26.10	0.006	0.97	True	True	0.998	0.002	True	True	carcinogen	low reliability	inactive	experimental value
BaP-50	-29.376	49.98	-72.928	14.03	-85.163	-8.57	0.811	0.17	True	True	1.000	0.000	True	True	non-carcinogen	low reliability	inactive	low reliability
BaP-51	-20.605	64.92	-39.522	53.41	-70.398	10.25	1.000	-0.02	False	True	0.000	1.000	True	True	non-carcinogen	good reliability	inactive	moderate reliability
BaP-52	-30.563	47.96	-38.615	54.48	-94.613	-20.62	0.002	0.97	True	True	0.000	1.000	True	True	non-carcinogen	low reliability	inactive	experimental value
BaP-53	-26.901	54.20	-20.931	75.33	-74.981	4.41	0.013	0.96	True	True	0.000	1.000	True	True	non-carcinogen	experimental value	inactive	good reliability
BaP-54	-32.255	45.08	-81.025	4.49	-93.135	-18.73	0.940	0.04	True	True	1.000	0.000	True	True	carcinogen	moderate reliability	inactive	low reliability
BaP-55	-49.801	15.21	-81.273	4.20	-73.915	5.77	0.999	-0.02	True	True	1.000	0.000	True	True	carcinogen	low reliability	inactive	low reliability
BaP-56	-43.986	25.11	-110.472	-30.22	-80.497	-2.62	0.999	-0.02	True	True	1.000	0.000	True	True	carcinogen	low reliability	inactive	low reliability

BaP-57	-69.406	-18.17	-67.997	19.85	-97.536	-24.34	0.999	-0.02	True	True	0.999	0.001	True	True	carcinogen	low reliability	active	good reliability
BaP-58	-35.831	38.99	-77.125	9.09	-77.687	0.96	0.999	-0.02	True	True	1.000	0.000	True	True	carcinogen	low reliability	inactive	low reliability
BaP-59	-62.374	-6.20	-103.716	-22.26	-68.835	12.25	0.999	-0.02	True	True	0.999	0.001	True	True	carcinogen	low reliability	active	good reliability
BaP-60	-29.200	50.28	-88.611	-4.45	-141.796	-80.77	0.994	-0.02	True	True	0.881	0.119	True	True	carcinogen	low reliability	inactive	good reliability
BaP-61	-41.279	29.72	-89.250	-5.21	-120.243	-53.29	0.973	0.00	True	True	0.000	1.000	False	False	carcinogen	moderate reliability	inactive	good reliability
BaP-62	-45.934	21.79	-70.065	17.41	-44.850	42.82	0.999	-0.02	True	True	1.000	0.000	True	True	carcinogen	low reliability	active	good reliability
BaP-63	-47.097	19.81	-72.645	14.37	-48.304	38.42	0.999	-0.02	True	True	1.000	0.000	True	True	carcinogen	low reliability	active	good reliability
BaP-64	-63.109	-7.45	-89.511	-5.51	-118.750	-51.39	0.996	-0.02	True	True	0.999	0.001	True	True	carcinogen	low reliability	active	moderate reliability
BaP-65	-40.324	31.34	-55.791	34.24	-44.558	43.20	0.976	0.00	True	True	0.205	0.795	True	True	carcinogen	moderate reliability	active	good reliability
BaP-66	-38.238	34.89	-49.127	42.09	-34.612	55.88	—	—	—	—	—	—	—	—	carcinogen	low reliability	inactive	good reliability
BaP-67	-44.691	23.91	-74.939	11.66	-50.871	35.15	0.999	-0.02	True	True	1.000	0.000	True	True	carcinogen	low reliability	active	good reliability
BaP-68	-45.988	21.70	-76.976	9.26	-55.672	29.03	0.999	-0.02	True	True	1.000	0.000	True	True	carcinogen	low reliability	active	good reliability
BaP-69	-57.840	1.52	-65.947	22.26	-80.498	-2.62	0.996	-0.02	True	True	0.999	0.001	True	True	carcinogen	low reliability	active	moderate reliability

BaP-70	-68.186	-16.10	-125.057	-47.41	-129.878	-65.57	0.976	0.00	True	True	0.169	0.831	True	True	carcinogen	moderate reliability	active	good reliability
BaP-71	-74.787	-27.34	-97.150	-14.52	-118.113	-50.58	0.940	0.04	True	True	0.020	0.980	True	True	carcinogen	moderate reliability	inactive	moderate reliability
BaP-72	-74.153	-26.26	-100.728	-18.74	-104.728	-33.51	0.000	0.98	True	True	1.000	0.000	True	True	carcinogen	moderate reliability	active	low reliability
BaP-73	-54.468	7.26	-100.420	-18.37	-66.790	14.85	0.999	-0.02	True	True	1.000	0.000	True	True	carcinogen	low reliability	inactive	low reliability
BaP-74	-40.547	30.96	-68.693	19.03	-49.357	37.08	0.999	-0.02	True	True	1.000	0.000	True	True	carcinogen	low reliability	active	good reliability
BaP-75	-34.878	40.61	-58.329	31.24	-52.326	33.29	0.999	-0.02	True	True	1.000	0.000	True	True	carcinogen	low reliability	active	good reliability
BaP-76	-53.285	9.27	-86.694	-2.19	-72.028	8.18	0.999	-0.02	True	True	1.000	0.000	True	True	carcinogen	low reliability	inactive	low reliability
**BbF**	-44.365	0.00	-103.742	0.00	-100.331	0.00	0.976	0.00	True	True	1.000	0.000	True	True	carcinogen	good reliability	active	low reliability
BbF-1	-50.581	-14.01	-108.224	-4.32	-90.713	9.59	0.999	-0.02	True	True	1.000	0.000	True	True	carcinogen	low reliability	active	good reliability
BbF-2	-47.302	-6.62	-90.475	12.79	-78.683	21.58	0.999	-0.02	True	True	1.000	0.000	True	True	carcinogen	low reliability	active	good reliability
BbF-3	-43.551	1.83	-89.495	13.73	-47.563	52.59	0.996	-0.02	True	True	1.000	0.000	True	True	carcinogen	moderate reliability	active	moderate reliability
BbF-4	-45.657	-2.91	-85.228	17.85	-102.139	-1.80	0.996	-0.02	True	True	1.000	0.000	True	True	non-carcinogen	moderate reliability	active	moderate reliability
BbF-5	-49.091	-10.65	-111.757	-7.73	-112.345	-11.97	0.996	-0.02	True	True	1.000	0.000	True	True	carcinogen	moderate reliability	active	moderate reliability

BbF-6	-46.277	-4.31	-98.012	5.52	-94.133	6.18	0.996	-0.02	True	True	1.000	0.000	True	True	non-carcinogen	moderate reliability	active	moderate reliability
BbF-7	-47.020	-5.98	-91.181	12.11	-87.610	12.68	0.996	-0.02	True	True	1.000	0.000	True	True	carcinogen	moderate reliability	active	low reliability
BbF-8	-51.588	-16.28	-94.296	9.11	-43.364	56.78	0.996	-0.02	True	True	1.000	0.000	True	True	carcinogen	moderate reliability	active	good reliability
BbF-9	-48.164	-8.56	-116.554	-12.35	-54.220	45.96	0.996	-0.02	True	True	1.000	0.000	True	True	carcinogen	moderate reliability	active	moderate reliability
BbF-10	-59.001	-32.99	-107.599	-3.72	-62.798	37.41	0.996	-0.02	True	True	1.000	0.000	True	True	carcinogen	moderate reliability	active	low reliability
BbF-11	-40.462	8.80	-86.208	16.90	-57.059	43.13	0.941	0.04	True	True	0.000	1.000	False	False	carcinogen	low reliability	inactive	low reliability
BbF-12	-54.891	-23.73	-106.386	-2.55	-104.155	-3.81	0.074	0.90	True	True	0.999	0.001	True	True	carcinogen	low reliability	inactive	low reliability
BbF-13	-43.535	1.87	-81.551	21.39	-101.473	-1.14	0.484	0.49	True	True	1.000	0.000	True	True	carcinogen	moderate reliability	inactive	low reliability
BbF-14	-38.117	14.08	-73.867	28.80	-47.865	52.29	0.144	0.83	True	True	1.000	0.000	True	True	non-carcinogen	low reliability	inactive	low reliability
BbF-15	-41.990	5.35	-68.560	33.91	-38.920	61.21	—	—	—	—	—	—	—	—	carcinogen	moderate reliability	inactive	low reliability
BbF-16	-43.779	1.32	-84.632	18.42	-123.242	-22.84	0.158	0.82	True	True	1.000	0.000	True	True	carcinogen	moderate reliability	active	good reliability
BbF-17	-48.037	-8.28	-74.223	28.45	-77.758	22.50	0.156	0.82	True	True	1.000	0.000	True	True	carcinogen	moderate reliability	active	good reliability
**BghiP**	-64.460	0.00	-121.288	0.00	-98.467	0.00	1.000	0.00	True	True	1.000	0.000	True	True	carcinogen	good reliability	active	low reliability

BghiP-1	-39.270	39.08	-69.481	42.71	-54.909	44.24	0.000	1.00	True	True	1.000	0.000	True	True	carcinogen	moderate reliability	active	moderate reliability
BghiP-2	-46.556	27.78	-59.034	51.33	-55.482	43.65	0.003	1.00	True	True	0.000	1.000	True	True	non-carcinogen	good reliability	inactive	low reliability
BghiP-3	-26.335	59.15	-56.385	53.51	-86.731	11.92	0.997	0.00	True	True	0.926	0.074	False	False	non-carcinogen	good reliability	inactive	low reliability
BghiP-4	-47.646	26.08	-76.430	36.98	-127.607	-29.59	1.000	0.00	False	False	0.000	1.000	True	True	non-carcinogen	moderate reliability	inactive	low reliability
BghiP-5	-64.769	-0.48	-128.447	-5.90	-89.615	8.99	1.000	0.00	True	True	1.000	0.000	True	True	carcinogen	good reliability	active	low reliability
BghiP-6	-447.297	-593.91	-939.422	-674.54	-618.118	-527.74	1.000	0.00	True	True	0.999	0.001	True	True	carcinogen	moderate reliability	active	good reliability
BghiP-7	-42.948	33.37	-43.473	64.16	-47.826	51.43	1.000	0.00	False	False	1.000	0.000	True	True	carcinogen	moderate reliability	active	low reliability
BghiP-8	-147.521	-128.86	-504.084	-315.61	-195.837	-98.89	1.000	0.00	True	True	1.000	0.000	True	True	carcinogen	moderate reliability	active	good reliability
BghiP-9	-65.646	-1.84	-108.141	10.84	-79.161	19.61	1.000	0.00	False	False	1.000	0.000	True	True	carcinogen	moderate reliability	active	low reliability
BghiP-10	-67.609	-4.89	-115.703	4.60	-82.089	16.63	0.998	0.00	True	True	1.000	0.000	True	True	carcinogen	moderate reliability	active	good reliability
BghiP-11	-60.879	5.56	-115.568	4.72	-84.501	14.18	1.000	0.00	True	True	1.000	0.000	True	True	carcinogen	good reliability	active	low reliability
BghiP-12	-42.194	34.54	-86.534	28.65	-73.764	25.09	1.000	0.00	True	True	1.000	0.000	True	True	carcinogen	low reliability	active	good reliability
BghiP-13	-67.309	-4.42	-82.378	32.08	-90.944	7.64	0.999	0.00	True	True	1.000	0.000	True	True	carcinogen	low reliability	active	low reliability
BghiP-14	-57.478	10.83	-98.392	18.88	-74.366	24.48	0.998	0.00	True	True	1.000	0.000	True	True	carcinogen	low reliability	active	good reliability

BghiP-15	-45.099	30.04	-85.635	29.40	-78.260	20.52	1.000	0.00	True	True	1.000	0.000	True	True	carcinogen	moderate reliability	inactive	low reliability
BghiP-16	-286.686	-344.75	-635.185	-423.70	-247.598	-151.45	1.000	0.00	False	True	0.002	0.998	False	True	non-carcinogen	moderate reliability	inactive	low reliability
BghiP-17	-295.834	-358.94	-640.026	-427.69	-279.579	-183.93	1.000	0.00	False	True	0.000	1.000	True	True	non-carcinogen	moderate reliability	inactive	good reliability
**BkF**	-50.842	0.00	-112.373	0.00	-81.132	0.00	0.990	0.00	True	True	1.000	0.000	True	True	carcinogen	good reliability	active	low reliability
BkF-1	-48.859	3.90	-92.277	17.88	-69.994	13.73	0.996	-0.01	True	True	1.000	0.000	True	True	carcinogen	moderate reliability	active	good reliability
BkF-2	-60.108	-18.23	-113.340	-0.86	-63.227	22.07	0.996	-0.01	True	True	1.000	0.000	True	True	non-carcinogen	moderate reliability	active	moderate reliability
BkF-3	-48.353	4.90	-105.613	6.02	-52.012	35.89	0.996	-0.01	True	True	1.000	0.000	True	True	non-carcinogen	moderate reliability	active	good reliability
BkF-4	-35.219	30.73	-66.048	41.22	-46.886	42.21	0.999	-0.01	True	True	1.000	0.000	True	True	carcinogen	moderate reliability	active	moderate reliability
BkF-5	-61.254	-20.48	-105.522	6.10	-72.269	10.92	0.999	-0.01	True	True	1.000	0.000	True	True	carcinogen	moderate reliability	active	moderate reliability
BkF-6	-61.577	-21.11	-98.522	12.33	-53.231	34.39	0.999	-0.01	True	True	1.000	0.000	True	True	carcinogen	low reliability	active	good reliability
BkF-7	-34.445	32.25	-67.817	39.65	-50.362	37.93	0.996	-0.01	True	True	0.565	0.435	True	True	carcinogen	moderate reliability	inactive	moderate reliability
BkF-8	-66.749	-31.29	-94.001	16.35	-56.487	30.38	0.976	0.01	True	True	0.197	0.803	True	True	carcinogen	moderate reliability	active	moderate reliability
BkF-9	-52.965	-4.18	-85.169	24.21	-68.029	16.15	0.992	0.00	True	True	0.948	0.052	True	True	carcinogen	low reliability	inactive	low reliability

BkF-10	-26.637	47.61	-71.223	36.62	-53.063	34.60	0.019	0.97	True	True	0.999	0.001	True	True	non-carcinogen	low reliability	inactive	low reliability
BkF-11	-393.264	-673.50	-728.263	-548.08	33.417	141.19	0.905	0.09	True	True	0.001	0.999	False	False	non-carcinogen	low reliability	inactive	low reliability
BkF-12	-33.676	33.76	-90.277	19.66	-71.204	12.24	0.022	0.97	True	True	1.000	0.000	True	True	non-carcinogen	good reliability	inactive	low reliability
BkF-13	-35.104	30.95	-97.770	13.00	-77.121	4.94	0.053	0.94	True	True	0.997	0.003	True	True	non-carcinogen	good reliability	inactive	moderate reliability
BkF-14	-33.846	33.43	-63.135	43.82	-62.404	23.08	0.368	0.62	True	True	1.000	0.000	True	True	non-carcinogen	good reliability	inactive	moderate reliability
BkF-15	-28.046	44.84	-50.170	55.35	-59.393	26.79	0.089	0.90	True	True	0.995	0.005	True	True	non-carcinogen	moderate reliability	inactive	low reliability
BkF-16	-28.417	44.11	-48.227	57.08	-93.111	-14.76	0.089	0.90	True	True	1.000	0.000	True	True	non-carcinogen	moderate reliability	inactive	moderate reliability
BkF-17	-35.297	30.58	-69.253	38.37	-94.274	-16.20	0.077	0.91	True	True	1.000	0.000	True	True	carcinogen	low reliability	inactive	moderate reliability
BkF-18	-30.448	40.11	-54.212	51.76	-86.344	-6.42	0.067	0.92	True	True	0.000	1.000	False	False	non-carcinogen	good reliability	inactive	good reliability
**CHR**	-66.290	0.00	-83.232	0.00	-84.175	0.00	0.720	0.00	True	True	0.999	0.000	True	True	carcinogen	good reliability	inactive	experimental value
CHR-1	-30.392	54.15	-41.395	50.27	-57.965	31.14	0.006	0.71	True	True	0.998	0.001	True	True	carcinogen	low reliability	inactive	experimental value
CHR-2	-42.028	36.60	-73.514	11.68	-95.598	-13.57	0.006	0.71	True	True	0.979	0.020	True	True	non-carcinogen	moderate reliability	active	experimental value
CHR-3	-39.502	40.41	-86.488	-3.91	-85.910	-2.06	0.864	-0.14	True	True	1.000	-0.001	True	True	carcinogen	good reliability	active	moderate reliability

CHR-4	-33.665	49.22	-72.113	13.36	-71.947	14.53	0.473	0.25	True	True	1.000	-0.001	True	True	carcinogen	low reliability	inactive	moderate reliability
CHR-5	-13.018	80.36	-63.360	23.88	-119.656	-42.15	0.028	0.69	True	True	0.372	0.627	True	True	non-carcinogen	low reliability	active	moderate reliability
CHR-6	-26.776	59.61	-39.618	52.40	-99.385	-18.07	0.001	0.72	True	True	0.000	0.999	True	True	non-carcinogen	low reliability	inactive	good reliability
CHR-7	-29.201	55.95	-47.557	42.86	-63.309	24.79	0.034	0.69	True	True	0.999	0.000	True	True	non-carcinogen	low reliability	active	moderate reliability
CHR-8	-39.408	40.55	-40.093	51.83	-94.745	-12.56	0.004	0.72	True	True	0.000	0.999	True	True	carcinogen	low reliability	inactive	good reliability
CHR-9	-26.187	60.50	-15.457	81.43	-64.113	23.83	0.013	0.71	True	True	0.000	0.999	True	True	carcinogen	experimental value	inactive	experimental value
CHR-10	-31.267	52.83	-64.740	22.22	-79.435	5.63	0.253	0.47	True	True	1.000	-0.001	True	True	carcinogen	low reliability	active	good reliability
CHR-11	-36.788	44.50	-74.395	10.62	-79.389	5.69	0.961	-0.24	True	True	1.000	-0.001	True	True	carcinogen	low reliability	inactive	low reliability
**DahA**	-66.480	0.00	-105.255	0.00	-81.625	0.00	0.988	0.00	True	True	1.000	0.000	True	True	carcinogen	good reliability	active	low reliability
DahA-1	-57.132	14.06	-98.078	6.82	-62.555	23.36	0.996	-0.01	True	True	1.000	0.000	True	True	carcinogen	moderate reliability	active	low reliability
DahA-2	-46.248	30.43	-86.364	17.95	-82.789	-1.43	0.238	0.75	False	True	0.000	1.000	True	True	non-carcinogen	low reliability	inactive	good reliability
DahA-3	-41.824	37.09	-72.114	31.49	-73.879	9.49	0.776	0.21	True	True	0.923	0.077	True	True	non-carcinogen	low reliability	inactive	low reliability
DahA-4	-39.852	40.05	-86.600	17.72	-74.129	9.18	0.999	-0.01	True	True	0.383	0.617	True	True	non-carcinogen	low reliability	inactive	low reliability

DahA-5	-56.130	15.57	-42.755	59.38	-74.465	8.77	1.000	-0.01	False	False	0.148	0.852	True	True	carcinogen	good reliability	inactive	good reliability
DahA-6	-42.028	36.78	-96.642	8.18	-136.040	-66.66	0.001	0.99	True	True	0.001	0.999	True	True	carcinogen	moderate reliability	inactive	moderate reliability
DahA-7	-43.381	34.75	-56.436	46.38	-66.963	17.96	0.000	0.99	False	False	1.000	0.000	True	True	non-carcinogen	low reliability	inactive	low reliability
**FLR**	-41.383	0.00	-67.423	0.00	-83.469	0.00	0.999	0.00	True	True	0.493	0.000	True	True	non-carcinogen	low reliability	inactive	moderate reliability
FLR-1	-31.152	24.72	-82.001	-21.62	-54.768	34.39	0.999	0.00	True	True	0.997	-0.504	True	True	carcinogen	good reliability	active	experimental value
FLR-2	-42.987	-3.88	-75.723	-12.31	-68.285	18.19	0.999	0.00	True	True	0.992	-0.499	True	True	non-carcinogen	low reliability	inactive	low reliability
FLR-3	-34.156	17.46	-87.784	-30.20	-62.063	25.65	0.089	0.91	True	True	0.930	-0.437	True	True	non-carcinogen	low reliability	inactive	low reliability
FLR-4	-51.128	-23.55	-90.006	-33.49	-71.741	14.05	0.794	0.21	True	True	0.991	-0.498	True	True	carcinogen	good reliability	active	low reliability
FLR-5	-21.245	48.66	-49.655	26.35	-72.946	12.61	0.089	0.91	True	True	1.000	-0.507	True	True	non-carcinogen	moderate reliability	inactive	low reliability
FLR-6	-27.212	34.24	-64.820	3.86	-88.365	-5.87	0.243	0.76	True	True	0.004	0.489	True	True	non-carcinogen	low reliability	inactive	moderate reliability
FLR-7	-26.918	34.95	-73.338	-8.77	-90.844	-8.84	0.653	0.35	True	True	0.004	0.489	True	True	non-carcinogen	low reliability	inactive	low reliability
FLR-8	-24.840	39.98	-57.754	14.34	-79.425	4.84	1.000	0.00	True	True	0.004	0.489	True	True	non-carcinogen	low reliability	inactive	good reliability
FLR-9	-20.103	51.42	-41.432	38.55	-83.822	-0.42	0.954	0.05	True	True	0.198	0.295	True	True	carcinogen	moderate reliability	inactive	low reliability

FLR-10	-33.267	19.61	-91.657	-35.94	-92.100	-10.34	0.089	0.91	True	True	1.000	-0.507	True	True	non-carcinogen	low reliability	inactive	low reliability
FLR-11	-30.554	26.17	-92.835	-37.69	-90.725	-8.69	0.527	0.47	True	True	1.000	-0.507	True	True	carcinogen	good reliability	inactive	low reliability
FLR-12	-36.988	10.62	-96.459	-43.07	-92.214	-10.48	0.150	0.85	True	True	1.000	-0.507	True	True	carcinogen	good reliability	inactive	low reliability
FLR-13	-43.499	-5.11	-62.901	6.71	-92.485	-10.80	0.134	0.87	True	True	0.000	0.493	True	True	carcinogen	moderate reliability	inactive	moderate reliability
FLR-14	-35.069	15.26	-44.586	33.87	-89.798	-7.58	1.000	0.00	True	True	0.991	-0.498	True	True	carcinogen	low reliability	inactive	low reliability
FLR-15	-52.982	-28.03	-64.361	4.54	-103.909	-24.49	0.003	1.00	True	True	0.000	0.493	True	True	non-carcinogen	good reliability	inactive	good reliability
FLR-16	-41.677	-0.71	-55.564	17.59	-121.413	-45.46	0.006	0.99	True	True	0.000	0.493	True	True	non-carcinogen	experimental value	inactive	moderate reliability
FLR-17	-46.326	-11.94	-79.730	-18.25	-57.079	31.62	1.000	0.00	True	True	0.998	-0.505	True	True	carcinogen	moderate reliability	inactive	low reliability
FLR-18	-28.472	31.20	-80.257	-19.04	-92.418	-10.72	0.857	0.14	True	True	1.000	-0.507	True	True	non-carcinogen	moderate reliability	inactive	low reliability
FLR-19	-20.423	50.65	-75.753	-12.35	-103.312	-23.77	0.017	0.98	True	True	0.458	0.035	True	True	non-carcinogen	moderate reliability	inactive	moderate reliability
FLR-20	-27.628	33.24	-91.034	-35.02	-104.719	-25.46	0.486	0.51	True	True	0.039	0.454	True	True	non-carcinogen	low reliability	inactive	low reliability
FLR-21	-27.204	34.26	-89.834	-33.24	-94.250	-12.92	0.998	0.00	True	True	0.984	-0.491	True	True	non-carcinogen	low reliability	inactive	good reliability
FLR-22	-28.056	32.20	-87.860	-30.31	-99.034	-18.65	0.015	0.98	True	True	0.536	-0.043	True	True	non-carcinogen	moderate reliability	inactive	good reliability

FLR-23	-17.263	58.28	-56.446	16.28	-123.091	-47.47	0.851	0.15	True	True	0.000	0.493	True	True	carcinogen	experimental value	inactive	low reliability
FLR-24	-29.812	27.96	-41.269	38.79	-72.615	13.00	0.819	0.18	True	True	0.000	0.493	True	True	non-carcinogen	moderate reliability	inactive	low reliability
FLR-25	-32.344	21.84	-24.994	62.93	-82.219	1.50	0.001	1.00	True	True	0.000	0.493	True	True	non-carcinogen	low reliability	inactive	good reliability
FLR-26	-36.566	11.64	-19.218	71.50	-88.052	-5.49	0.006	0.99	True	True	0.000	0.493	True	True	non-carcinogen	good reliability	inactive	good reliability
FLR-27	-29.119	29.64	-29.651	56.02	-80.222	3.89	0.013	0.99	True	True	0.000	0.493	True	True	carcinogen	experimental value	inactive	experimental value
FLR-28	-25.304	38.85	-39.536	41.36	-83.491	-0.03	0.004	1.00	True	True	0.000	0.493	True	True	non-carcinogen	experimental value	inactive	experimental value
**FRT**	-49.042	0.00	-104.230	0.00	-76.780	0.00	0.766	0.00	True	True	0.998	0.000	True	True	carcinogen	low reliability	inactive	moderate reliability
FRT-1	-41.684	15.00	-86.992	16.54	-53.495	30.33	0.876	-0.11	True	True	1.000	-0.002	True	True	carcinogen	low reliability	inactive	moderate reliability
FRT-2	-45.789	6.63	-86.716	16.80	-59.850	22.05	0.876	-0.11	True	True	1.000	-0.002	True	True	carcinogen	low reliability	inactive	good reliability
FRT-3	-35.795	27.01	-80.005	23.24	-46.241	39.77	0.876	-0.11	True	True	1.000	-0.002	True	True	carcinogen	low reliability	inactive	moderate reliability
FRT-4	-34.371	29.92	-69.047	33.76	-46.341	39.64	0.978	-0.21	True	True	0.998	0.000	True	True	carcinogen	low reliability	inactive	low reliability
FRT-5	-36.251	26.08	-66.938	35.78	-48.686	36.59	—	—	—	—	—	—	—	—	carcinogen	good reliability	inactive	low reliability
FRT-6	-59.133	-20.58	-83.853	19.55	-72.433	5.66	1.000	-0.23	False	True	0.000	0.998	False	False	non-carcinogen	low reliability	inactive	moderate reliability

FRT-7	-43.805	10.68	-93.251	10.53	-52.021	32.25	1.000	-0.23	False	True	0.000	0.998	False	False	non-carcinogen	low reliability	inactive	moderate reliability
FRT-8	-40.507	17.40	-65.524	37.14	-55.174	28.14	1.000	-0.23	True	True	0.000	0.998	False	False	non-carcinogen	low reliability	inactive	low reliability
FRT-9	-65.143	-32.83	-122.943	-17.95	26.481	134.49	1.000	-0.23	True	True	0.000	0.998	False	False	non-carcinogen	low reliability	inactive	moderate reliability
FRT-10	-31.478	35.81	-67.110	35.61	-51.182	33.34	1.000	-0.23	False	False	0.000	0.998	False	False	non-carcinogen	low reliability	inactive	low reliability
FRT-11	-82.714	-68.66	-106.703	-2.37	41.159	153.61	1.000	-0.23	False	False	0.000	0.998	False	False	carcinogen	low reliability	inactive	low reliability
FRT-12	-37.569	23.39	-58.762	43.62	-54.079	29.57	0.004	0.76	True	True	0.999	-0.001	True	True	carcinogen	low reliability	inactive	low reliability
FRT-13	-49.139	-0.20	-87.386	16.16	-56.502	26.41	0.978	-0.21	True	True	0.999	-0.001	True	True	carcinogen	low reliability	inactive	moderate reliability
FRT-14	-66.416	-35.43	-109.085	-4.66	-77.335	-0.72	0.999	-0.23	True	True	1.000	-0.002	True	True	carcinogen	low reliability	active	good reliability
FRT-15	-30.366	38.08	-49.051	52.94	-59.841	22.06	0.019	0.75	True	True	1.000	-0.002	True	True	non-carcinogen	low reliability	inactive	moderate reliability
FRT-16	-27.094	44.75	-68.529	34.25	-80.832	-5.28	0.649	0.12	True	True	0.582	0.416	False	False	carcinogen	low reliability	inactive	low reliability
FRT-17	-27.796	43.32	-47.094	54.82	-67.487	12.10	0.993	-0.23	True	True	1.000	-0.002	True	True	non-carcinogen	low reliability	inactive	moderate reliability
FRT-18	-28.382	42.13	-59.480	42.93	-66.710	13.12	0.996	-0.23	True	True	1.000	-0.002	True	True	non-carcinogen	moderate reliability	inactive	moderate reliability
FRT-19	-34.574	29.50	-79.344	23.88	-126.774	-65.11	0.997	-0.23	True	True	1.000	-0.002	True	True	carcinogen	moderate reliability	inactive	moderate reliability

FRT-20	-31.365	36.04	-57.490	44.84	-57.495	25.12	0.999	-0.23	True	True	1.000	-0.002	True	True	carcinogen	moderate reliability	inactive	low reliability
FRT-21	-31.006	36.78	-61.454	41.04	-53.645	30.13	—	—	—	—	—	—	—	—	carcinogen	good reliability	inactive	low reliability
FRT-22	-34.941	28.75	-90.072	13.58	-48.057	37.41	0.978	-0.21	True	True	0.998	0.000	True	True	carcinogen	low reliability	inactive	moderate reliability
FRT-23	-36.543	25.49	-96.774	7.15	-49.526	35.50	0.999	-0.23	True	True	1.000	-0.002	True	True	carcinogen	low reliability	inactive	low reliability
FRT-24	-30.602	37.60	-72.344	30.59	-87.770	-14.31	0.001	0.77	True	True	1.000	-0.002	True	True	carcinogen	low reliability	inactive	moderate reliability
FRT-25	-30.126	38.57	-81.745	21.57	-86.529	-12.70	0.000	0.77	True	True	0.526	0.472	False	False	non-carcinogen	low reliability	inactive	low reliability
FRT-26	-32.501	33.73	-106.609	-2.28	-95.461	-24.33	0.007	0.76	True	True	1.000	-0.002	True	True	carcinogen	good reliability	inactive	low reliability
FRT-27	-30.947	36.90	-86.188	17.31	-62.191	19.00	0.001	0.77	True	True	0.329	0.669	True	True	carcinogen	low reliability	inactive	low reliability
FRT-28	-33.267	32.17	-91.657	12.06	-92.100	-19.95	0.089	0.68	True	True	1.000	-0.002	True	True	non-carcinogen	low reliability	inactive	low reliability
FRT-29	-36.121	26.35	-82.033	21.30	-59.812	22.10	0.089	0.68	True	True	0.930	0.068	True	True	non-carcinogen	low reliability	inactive	low reliability
FRT-30	-66.260	-35.11	-87.999	15.57	-90.495	-17.86	0.473	0.29	True	True	0.000	0.998	True	True	carcinogen	low reliability	inactive	low reliability
FRT-31	-55.152	-12.46	-60.016	42.42	-118.953	-54.93	0.012	0.75	True	True	0.000	0.998	True	True	non-carcinogen	low reliability	inactive	good reliability
FRT-32	-29.404	40.04	-44.018	57.77	-41.565	45.86	1.000	-0.23	False	False	0.760	0.238	True	True	carcinogen	low reliability	inactive	low reliability

FRT-33	-42.937	12.45	-90.581	13.10	-48.995	36.19	0.992	-0.23	True	True	0.948	0.050	True	True	carcinogen	low reliability	inactive	low reliability
FRT-34	-57.738	-17.73	-93.182	10.60	-52.667	31.41	1.000	-0.23	True	True	0.998	0.000	True	True	carcinogen	low reliability	inactive	low reliability
FRT-35	-34.625	29.40	-53.440	48.73	-88.116	-14.76	0.067	0.70	True	True	0.000	0.998	False	False	non-carcinogen	good reliability	inactive	good reliability
FRT-36	-35.494	27.63	-67.593	35.15	-51.477	32.96	0.105	0.66	True	True	1.000	-0.002	True	True	carcinogen	low reliability	inactive	low reliability
FRT-37	-34.371	29.92	-69.047	33.76	-46.341	39.64	0.978	-0.21	True	True	0.998	0.000	True	True	carcinogen	low reliability	inactive	low reliability
FRT-38	-38.844	20.79	-72.505	30.44	-48.901	36.31	0.999	-0.23	True	True	1.000	-0.002	True	True	carcinogen	low reliability	inactive	low reliability
FRT-39	-28.689	41.50	-66.906	35.81	-56.522	26.38	0.001	0.77	True	True	1.000	-0.002	True	True	carcinogen	good reliability	inactive	good reliability
FRT-40	-42.355	13.64	-78.942	24.26	-126.953	-65.35	0.462	0.30	True	True	0.000	0.998	True	True	carcinogen	low reliability	inactive	low reliability
FRT-41	-76.605	-56.20	-91.533	12.18	-128.579	-67.46	1.000	-0.23	True	True	0.426	0.572	True	True	non-carcinogen	low reliability	inactive	low reliability
FRT-42	-40.334	17.76	-35.199	66.23	-86.628	-12.83	0.134	0.63	True	True	0.000	0.998	True	True	carcinogen	moderate reliability	inactive	moderate reliability
FRT-43	-63.922	-30.34	-92.298	11.45	-97.989	-27.62	1.000	-0.23	True	True	0.991	0.007	True	True	carcinogen	low reliability	inactive	low reliability
FRT-44	-45.740	6.73	-68.062	34.70	-100.328	-30.67	0.003	0.76	True	True	0.000	0.998	True	True	non-carcinogen	good reliability	inactive	good reliability
FRT-45	-32.628	33.47	-38.344	63.21	-100.974	-31.51	0.006	0.76	True	True	0.000	0.998	True	True	non-carcinogen	experimental value	inactive	moderate reliability

FRT-46	-34.755	29.13	-52.226	49.89	-64.022	16.62	0.089	0.68	True	True	0.900	0.098	True	True	carcinogen	low reliability	inactive	low reliability
FRT-47	-32.528	33.67	-56.817	45.49	-69.686	9.24	0.017	0.75	True	True	0.607	0.391	True	True	carcinogen	low reliability	active	low reliability
FRT-48	-43.949	10.38	-69.376	33.44	-64.852	15.54	0.535	0.23	True	True	0.000	0.998	True	True	non-carcinogen	low reliability	inactive	low reliability
FRT-49	-38.363	21.78	-87.227	16.31	-87.066	-13.40	0.359	0.41	True	True	0.926	0.072	True	True	non-carcinogen	low reliability	inactive	moderate reliability
FRT-50	-41.087	16.22	-91.609	12.11	-86.665	-12.87	0.746	0.02	True	True	0.675	0.323	True	True	carcinogen	low reliability	inactive	moderate reliability
FRT-51	-48.541	1.02	-94.517	9.32	-102.801	-33.89	0.998	-0.23	True	True	0.000	0.998	True	True	carcinogen	good reliability	inactive	moderate reliability
FRT-52	-15.194	69.02	-100.551	3.53	262.389	441.74	0.383	0.38	True	True	0.001	0.997	True	True	carcinogen	good reliability	inactive	moderate reliability
FRT-53	-49.460	-0.85	-43.963	57.82	-81.707	-6.42	0.003	0.76	True	True	0.000	0.998	True	True	non-carcinogen	good reliability	inactive	good reliability
FRT-54	-68.056	-38.77	-72.328	30.61	-68.203	11.17	0.000	0.77	False	False	0.000	0.998	True	True	carcinogen	good reliability	active	experimental value
**IcdP**	-65.071	0.00	-82.398	0.00	-108.142	0.00	0.999	0.00	True	True	1.000	0.000	True	True	carcinogen	good reliability	active	low reliability
IcdP-1	-40.223	38.19	-94.200	-14.32	-32.525	69.92	1.000	0.00	True	True	1.000	0.000	True	True	carcinogen	moderate reliability	active	good reliability
IcdP-2	-41.146	36.77	-96.162	-16.70	-47.692	55.90	1.000	0.00	True	True	1.000	0.000	True	True	carcinogen	moderate reliability	active	good reliability
IcdP-3	-50.829	21.89	-96.125	-16.66	-64.579	40.28	1.000	0.00	True	True	1.000	0.000	True	True	carcinogen	moderate reliability	active	good reliability

IcdP-4	-56.307	13.47	-93.791	-13.83	-62.285	42.40	1.000	0.00	True	True	1.000	0.000	True	True	carcinogen	moderate reliability	active	good reliability
IcdP-5	-39.161	39.82	-84.860	-2.99	-47.806	55.79	1.000	0.00	True	True	1.000	0.000	True	True	carcinogen	low reliability	active	good reliability
IcdP-6	-49.676	23.66	-89.534	-8.66	-51.776	52.12	1.000	0.00	True	True	1.000	0.000	True	True	carcinogen	moderate reliability	active	good reliability
IcdP-7	-56.887	12.58	-83.399	-1.21	-60.861	43.72	1.000	0.00	True	True	1.000	0.000	True	True	carcinogen	moderate reliability	active	good reliability
IcdP-8	-34.753	46.59	-76.796	6.80	-50.016	53.75	0.999	0.00	True	True	1.000	0.000	True	True	carcinogen	low reliability	active	low reliability
IcdP-9	-47.796	26.55	-100.539	-22.02	-61.135	43.47	1.000	0.00	True	True	1.000	0.000	True	True	carcinogen	moderate reliability	active	good reliability
IcdP-10	-25.304	61.11	-39.537	52.02	-83.491	22.80	0.004	1.00	True	True	0.000	1.000	True	True	non-carcinogen	experimental value	inactive	experimental value
IcdP-11	-43.098	33.77	-59.584	27.69	-56.341	47.90	0.000	1.00	True	True	0.000	1.000	True	True	non-carcinogen	low reliability	active	experimental value
IcdP-12	-55.962	14.00	-86.225	-4.64	-104.575	3.30	0.001	1.00	False	True	0.000	1.000	True	True	carcinogen	low reliability	inactive	moderate reliability
IcdP-13	-42.467	34.74	-92.749	-12.56	-76.265	29.48	0.238	0.76	False	True	0.000	1.000	True	True	non-carcinogen	low reliability	inactive	good reliability
IcdP-14	-70.988	-9.09	-80.635	2.14	-114.025	-5.44	0.003	1.00	True	True	0.000	1.000	True	True	non-carcinogen	low reliability	inactive	moderate reliability
IcdP-15	-28.516	56.18	-63.569	22.85	-85.652	20.80	0.000	1.00	False	False	0.000	1.000	True	True	carcinogen	low reliability	inactive	low reliability
IcdP-16	-61.922	4.84	-83.808	-1.71	-101.061	6.55	1.000	0.00	False	True	0.995	0.005	True	True	non-carcinogen	low reliability	inactive	good reliability

IcdP-17	-45.068	30.74	-62.495	24.15	-52.358	51.58	0.150	0.85	True	True	0.998	0.002	True	True	carcinogen	moderate reliability	active	moderate reliability
IcdP-18	-32.203	50.51	-74.676	9.37	-76.518	29.24	0.093	0.91	False	True	0.000	1.000	True	True	carcinogen	low reliability	inactive	low reliability
IcdP-19	-43.505	33.14	-89.833	-9.02	-70.234	35.05	0.889	0.11	True	True	1.000	0.000	True	True	carcinogen	moderate reliability	active	good reliability
IcdP-20	-53.863	17.22	-99.835	-21.16	-64.375	40.47	1.000	0.00	True	True	1.000	0.000	True	True	carcinogen	low reliability	active	good reliability
IcdP-21	-47.525	26.96	-81.808	0.72	-60.094	44.43	0.973	0.03	True	True	1.000	0.000	True	True	non-carcinogen	moderate reliability	active	low reliability
IcdP-22	-41.730	35.87	-79.942	2.98	-46.496	57.00	0.999	0.00	True	True	1.000	0.000	True	True	non-carcinogen	moderate reliability	inactive	low reliability
IcdP-23	-50.727	22.04	-86.517	-5.00	-45.118	58.28	0.976	0.02	True	True	1.000	0.000	True	True	non-carcinogen	moderate reliability	inactive	low reliability
IcdP-24	-35.339	45.69	-44.025	46.57	-56.969	47.32	0.974	0.03	True	True	0.000	1.000	False	False	carcinogen	moderate reliability	inactive	good reliability
IcdP-25	-36.428	44.02	-87.059	-5.66	-48.000	55.61	1.000	0.00	True	True	1.000	0.000	True	True	carcinogen	low reliability	active	good reliability
IcdP-26	-41.634	36.02	-90.262	-9.54	-46.759	56.76	1.000	0.00	True	True	1.000	0.000	True	True	carcinogen	low reliability	active	good reliability
IcdP-27	-37.054	43.06	-88.646	-7.58	-49.631	54.11	0.999	0.00	True	True	0.000	1.000	False	False	carcinogen	moderate reliability	inactive	moderate reliability
IcdP-28	-38.857	40.29	-81.300	1.33	-45.885	57.57	0.999	0.00	True	True	1.000	0.000	True	True	carcinogen	moderate reliability	active	moderate reliability
IcdP-29	-41.080	36.87	-88.604	-7.53	-45.631	57.80	0.999	0.00	True	True	1.000	0.000	True	True	carcinogen	low reliability	active	good reliability

IcdP-30	-39.264	39.66	-70.458	14.49	-51.984	51.93	0.003	1.00	True	True	1.000	0.000	True	True	carcinogen	low reliability	inactive	low reliability
IcdP-31	-26.578	59.16	-33.558	59.27	-51.347	52.52	0.154	0.85	True	True	0.000	1.000	True	True	non-carcinogen	low reliability	inactive	low reliability
IcdP-32	-42.257	35.06	-66.147	19.72	-51.861	52.04	0.081	0.92	True	True	0.001	0.999	True	True	non-carcinogen	low reliability	inactive	good reliability
IcdP-33	-25.438	60.91	-86.552	-5.04	-105.571	2.38	0.148	0.85	True	True	0.204	0.796	True	True	non-carcinogen	low reliability	inactive	low reliability
IcdP-34	-30.665	52.87	-61.369	25.52	-70.926	34.41	0.999	0.00	True	True	0.000	1.000	True	True	non-carcinogen	low reliability	inactive	low reliability
IcdP-35	-42.928	34.03	-48.728	40.86	-55.103	49.05	0.994	0.01	True	True	1.000	0.000	False	True	carcinogen	low reliability	inactive	moderate reliability
IcdP-36	-37.259	42.74	-104.145	-26.39	-47.323	56.24	1.000	0.00	True	True	1.000	0.000	True	True	carcinogen	moderate reliability	active	good reliability
IcdP-37	-49.693	23.63	-99.093	-20.26	-60.852	43.73	1.000	0.00	True	True	1.000	0.000	True	True	carcinogen	moderate reliability	active	good reliability
**NAP**	-26.336	0.00	-33.803	0.00	-51.637	0.00	0.154	0.00	True	True	0.000	0.000	True	True	non-carcinogen	low reliability	inactive	low reliability
NAP-1	-28.937	-9.88	-30.973	8.37	-52.474	-1.62	0.000	0.15	True	True	1.000	-1.000	True	True	non-carcinogen	moderate reliability	inactive	good reliability
NAP-2	-21.267	19.25	-57.998	-71.58	-80.869	-56.61	0.016	0.14	True	True	0.000	0.000	False	False	non-carcinogen	moderate reliability	inactive	moderate reliability
NAP-3	-29.201	-10.88	-47.557	-40.69	-63.309	-22.60	0.034	0.12	True	True	0.999	-0.999	True	True	non-carcinogen	low reliability	active	moderate reliability
NAP-4	-30.392	-15.40	-41.395	-22.46	-57.965	-12.25	0.006	0.15	True	True	0.998	-0.998	True	True	carcinogen	low reliability	inactive	experimental value

NAP-5	-16.318	38.04	-39.977	-18.26	-71.844	-39.13	0.012	0.14	True	True	0.996	-0.996	True	True	non-carcinogen	low reliability	inactive	low reliability
NAP-6	-43.902	-66.70	-66.943	-98.04	-47.258	8.48	1.000	-0.85	False	True	0.000	0.000	False	False	carcinogen	low reliability	inactive	low reliability
NAP-7	-33.079	-25.60	-52.636	-55.71	-65.589	-27.02	0.999	-0.85	True	True	0.999	-0.999	True	True	non-carcinogen	low reliability	inactive	low reliability
NAP-8	-42.028	-59.58	-73.514	-117.48	-95.597	-85.13	0.006	0.15	True	True	0.979	-0.979	True	True	non-carcinogen	moderate reliability	active	experimental value
NAP-9	-50.880	-93.20	-50.416	-49.15	-97.538	-88.89	0.129	0.03	True	True	0.284	-0.284	True	True	carcinogen	low reliability	inactive	moderate reliability
NAP-10	-45.092	-71.22	-60.944	-80.29	-91.279	-76.77	0.003	0.15	True	True	1.000	-1.000	True	True	non-carcinogen	low reliability	inactive	moderate reliability
NAP-11	-36.087	-37.03	-64.550	-90.96	-90.198	-74.68	0.129	0.03	True	True	0.112	-0.112	True	True	non-carcinogen	low reliability	active	moderate reliability
NAP-12	-33.342	-26.60	-49.568	-46.64	-61.413	-18.93	0.034	0.12	True	True	0.999	-0.999	True	True	non-carcinogen	moderate reliability	active	moderate reliability
NAP-13	-36.226	-37.55	-74.199	-119.50	-88.619	-71.62	0.010	0.14	True	True	0.000	0.000	True	True	non-carcinogen	low reliability	inactive	low reliability
NAP-14	-19.040	27.70	-38.888	-15.04	-70.903	-37.31	0.956	-0.80	False	False	0.000	0.000	False	False	non-carcinogen	moderate reliability	inactive	low reliability
NAP-15	-59.247	-124.97	-122.487	-262.36	-101.810	-97.16	0.005	0.15	True	True	0.030	-0.030	True	True	non-carcinogen	low reliability	inactive	good reliability
NAP-16	-32.001	-21.51	-59.140	-74.95	-56.459	-9.34	0.954	-0.80	False	True	0.000	0.000	False	False	non-carcinogen	low reliability	inactive	low reliability
NAP-17	-47.036	-78.60	-96.341	-185.01	-127.736	-147.37	0.012	0.14	False	False	0.000	0.000	False	False	non-carcinogen	low reliability	inactive	low reliability

NAP-18	-44.566	-69.22	-107.868	-219.11	-139.516	-170.19	0.011	0.14	False	False	0.000	0.000	False	False	non-carcinogen	low reliability	inactive	low reliability
NAP-19	— [a]	—	—	—	—	—	0.000	0.15	False	True	0.000	0.000	False	False	non-carcinogen	low reliability	inactive	low reliability
NAP-20	—	—	—	—	—	—	0.000	0.15	False	True	0.000	0.000	False	False	non-carcinogen	low reliability	inactive	low reliability
NAP-21	—	—	—	—	—	—	0.090	0.06	False	True	0.999	-0.999	False	False	non-carcinogen	low reliability	inactive	low reliability
NAP-22	—	—	—	—	—	—	0.000	0.15	False	True	0.011	-0.011	False	False	non-carcinogen	low reliability	inactive	low reliability
NAP-23	-25.790	2.07	-49.952	-47.77	-61.195	-18.51	0.016	0.14	True	True	0.000	0.000	False	False	non-carcinogen	moderate reliability	inactive	moderate reliability
NAP-24	-57.386	-117.90	-79.740	-135.90	-116.044	-124.73	0.013	0.14	True	True	1.000	-1.000	True	True	non-carcinogen	low reliability	inactive	moderate reliability
NAP-25	-27.988	-6.27	-32.205	4.73	-69.550	-34.69	0.008	0.15	True	True	0.000	0.000	True	True	carcinogen	moderate reliability	inactive	low reliability
NAP-26	-19.000	27.86	-53.297	-57.67	-120.144	-132.67	0.000	0.15	True	True	0.912	-0.912	True	True	carcinogen	moderate reliability	inactive	moderate reliability
NAP-27	-28.410	-7.88	-46.647	-38.00	-77.713	-50.50	0.004	0.15	True	True	1.000	-1.000	False	False	non-carcinogen	low reliability	active	low reliability
NAP-28	-39.416	-49.67	-26.206	22.47	-64.611	-25.13	0.004	0.15	True	True	0.000	0.000	True	True	carcinogen	low reliability	inactive	good reliability
NAP-29	-29.971	-13.80	-19.419	42.55	-75.395	-46.01	0.001	0.15	True	True	0.000	0.000	True	True	non-carcinogen	low reliability	inactive	good reliability
NAP-30	-47.185	-79.17	-44.916	-32.88	-70.743	-37.00	0.006	0.15	True	True	0.000	0.000	True	True	non-carcinogen	good reliability	inactive	good reliability

NAP-31	-29.119	-10.57	-29.651	12.28	-80.221	-55.36	0.013	0.14	True	True	0.000	0.000	True	True	carcinogen	experimental value	inactive	experimental value
NAP-32	-40.491	-53.75	-58.844	-74.08	-82.947	-60.63	0.228	-0.07	True	True	0.732	-0.732	True	True	non-carcinogen	moderate reliability	inactive	good reliability
**PHE**	-34.993	0.00	-54.440	0.00	-64.850	0.00	0.089	0.00	True	True	0.900	0.000	True	True	carcinogen	low reliability	inactive	low reliability
PHE-1	-31.814	9.08	-66.886	-22.86	-92.600	-42.79	0.150	-0.06	True	True	0.999	-0.099	True	True	non-carcinogen	low reliability	inactive	moderate reliability
PHE-2	-35.758	-2.19	-69.663	-27.96	-76.213	-17.52	0.150	-0.06	True	True	0.999	-0.099	True	True	carcinogen	moderate reliability	inactive	moderate reliability
PHE-3	-34.283	2.03	-49.665	8.77	-55.951	13.72	0.150	-0.06	True	True	0.998	-0.098	True	True	carcinogen	moderate reliability	active	moderate reliability
PHE-4	-40.942	-17.00	-60.937	-11.93	-54.703	15.65	0.150	-0.06	True	True	0.999	-0.099	True	True	carcinogen	moderate reliability	inactive	moderate reliability
PHE-5	-32.970	5.78	-57.551	-5.71	-64.954	-0.16	0.150	-0.06	True	True	1.000	-0.100	True	True	carcinogen	moderate reliability	inactive	moderate reliability
PHE-6	-44.090	-26.00	-82.981	-52.43	-86.445	-33.30	0.630	-0.54	True	True	0.001	0.899	True	True	carcinogen	low reliability	inactive	moderate reliability
PHE-7	-36.989	-5.70	-96.460	-77.19	-92.214	-42.20	0.150	-0.06	True	True	1.000	-0.100	True	True	carcinogen	good reliability	inactive	low reliability
PHE-8	-33.267	4.93	-91.658	-68.37	-92.101	-42.02	0.089	0.00	True	True	1.000	-0.100	True	True	non-carcinogen	low reliability	inactive	low reliability
PHE-9	-62.817	-79.51	-89.957	-65.24	-123.326	-90.17	0.067	0.02	True	True	1.000	-0.100	True	True	carcinogen	moderate reliability	inactive	low reliability
PHE-10	-16.413	53.10	37.312	168.54	-47.387	26.93	0.382	-0.29	True	True	1.000	-0.100	True	True	non-carcinogen	low reliability	active	moderate reliability

PHE-11	-31.214	10.80	-64.202	-17.93	-84.308	-30.00	0.527	-0.44	True	True	0.999	-0.099	True	True	non-carcinogen	low reliability	active	moderate reliability
PHE-12	-30.084	14.03	-84.386	-55.01	-106.463	-64.17	0.238	-0.15	True	True	1.000	-0.100	True	True	carcinogen	moderate reliability	inactive	low reliability
PHE-13	-28.532	18.46	-34.650	36.35	-49.248	24.06	0.135	-0.05	True	True	0.999	-0.099	True	True	non-carcinogen	low reliability	active	good reliability
PHE-14	-20.938	40.17	-64.380	-18.26	-94.670	-45.98	0.092	0.00	True	True	1.000	-0.100	True	True	non-carcinogen	low reliability	active	moderate reliability
PHE-15	-22.403	35.98	-72.055	-32.36	-97.329	-50.08	0.028	0.06	True	True	0.393	0.507	True	True	non-carcinogen	low reliability	active	moderate reliability
PHE-16	-29.934	14.46	-73.129	-34.33	-79.434	-22.49	0.000	0.09	True	True	0.993	-0.093	True	True	carcinogen	moderate reliability	inactive	moderate reliability
PHE-17	-49.352	-41.03	-73.414	-34.85	-92.264	-42.27	0.585	-0.50	True	True	0.980	-0.080	True	True	carcinogen	low reliability	inactive	moderate reliability
PHE-18	-49.177	-40.53	-73.250	-34.55	-98.149	-51.35	0.585	-0.50	True	True	0.980	-0.080	True	True	carcinogen	low reliability	inactive	moderate reliability
PHE-19	-38.750	-10.74	-58.407	-7.29	-75.914	-17.06	0.053	0.04	True	True	0.000	0.900	True	True	non-carcinogen	low reliability	inactive	moderate reliability
PHE-20	-9.317	73.37	30.128	155.34	-59.287	8.58	0.383	-0.29	True	True	1.000	-0.100	True	True	carcinogen	low reliability	inactive	moderate reliability
PHE-21	-39.556	-13.04	-69.049	-26.84	-61.327	5.43	0.527	-0.44	True	True	0.999	-0.099	True	True	carcinogen	low reliability	inactive	moderate reliability
PHE-22	-40.756	-16.47	-43.140	20.76	-53.518	17.47	0.227	-0.14	True	True	1.000	-0.100	True	True	carcinogen	moderate reliability	inactive	low reliability
PHE-23	-43.651	-24.74	-72.525	-33.22	-59.748	7.87	0.129	-0.04	True	True	0.999	-0.099	True	True	non-carcinogen	low reliability	active	good reliability

PHE-24	-38.853	-11.03	-62.665	-15.11	-49.058	24.35	0.087	0.00	True	True	1.000	-0.100	True	True	non-carcinogen	low reliability	active	moderate reliability
PHE-25	-30.683	12.32	-68.196	-25.27	-59.819	7.76	0.028	0.06	True	True	0.372	0.528	True	True	non-carcinogen	low reliability	active	moderate reliability
PHE-26	-38.580	-10.25	-59.052	-8.47	-64.679	0.26	0.000	0.09	True	True	0.980	-0.080	True	True	non-carcinogen	low reliability	inactive	moderate reliability
PHE-27	-24.588	29.73	-55.224	-1.44	-57.053	12.02	0.132	-0.04	True	True	0.398	0.502	True	True	non-carcinogen	low reliability	inactive	good reliability
PHE-28	-41.275	-17.95	-61.349	-12.69	-40.193	38.02	0.125	-0.04	True	True	0.417	0.483	True	True	carcinogen	moderate reliability	inactive	good reliability
PHE-29	-14.750	57.85	-77.255	-41.91	-123.002	-89.67	0.024	0.07	True	True	0.000	0.900	False	False	non-carcinogen	good reliability	inactive	good reliability
PHE-30	-29.201	16.55	-47.557	12.64	-63.310	2.37	0.034	0.06	True	True	0.999	-0.099	True	True	non-carcinogen	low reliability	active	moderate reliability
PHE-31	-38.044	-8.72	-63.630	-16.88	-79.768	-23.00	0.004	0.09	True	True	1.000	-0.100	False	False	non-carcinogen	low reliability	active	low reliability
PHE-32	-39.408	-12.62	-40.093	26.35	-94.745	-46.10	0.004	0.09	True	True	0.000	0.900	True	True	carcinogen	low reliability	inactive	good reliability
PHE-33	-41.797	-19.44	-43.145	20.75	-84.402	-30.15	0.001	0.09	True	True	0.000	0.900	True	True	non-carcinogen	low reliability	inactive	good reliability
PHE-34	-46.406	-32.62	-49.417	9.23	-85.981	-32.58	0.006	0.08	True	True	0.000	0.900	True	True	non-carcinogen	good reliability	inactive	good reliability
PHE-35	-19.000	45.70	-53.297	2.10	-120.144	-85.26	0.000	0.09	True	True	0.912	-0.012	True	True	carcinogen	moderate reliability	inactive	moderate reliability
PHE-36	-31.246	10.71	-39.737	27.01	-109.410	-68.71	0.004	0.09	True	True	0.960	-0.060	True	True	non-carcinogen	moderate reliability	inactive	low reliability

PHE-37	-51.397	-46.88	-64.476	-18.43	-88.576	-36.59	0.002	0.09	True	True	0.000	0.900	True	True	non-carcinogen	moderate reliability	inactive	good reliability
PHE-38	-45.738	-30.71	-68.054	-25.01	-100.323	-54.70	0.003	0.09	True	True	0.000	0.900	True	True	non-carcinogen	good reliability	inactive	good reliability
PHE-39	-52.042	-48.72	-55.691	-2.30	-92.942	-43.32	0.089	0.00	True	True	0.000	0.900	True	True	non-carcinogen	good reliability	inactive	low reliability
PHE-40	-35.218	-0.64	-15.446	71.63	-77.532	-19.56	0.006	0.08	True	True	0.000	0.900	True	True	non-carcinogen	experimental value	inactive	moderate reliability
PHE-41	-54.812	-56.64	-78.341	-43.90	-65.286	-0.67	0.003	0.09	True	True	1.000	-0.100	False	False	carcinogen	moderate reliability	inactive	low reliability
PHE-42	-46.255	-32.18	-75.719	-39.09	-76.507	-17.98	0.766	-0.68	True	True	0.306	0.594	True	True	carcinogen	low reliability	inactive	moderate reliability
PHE-43	-35.969	-2.79	-70.455	-29.42	-70.519	-8.74	0.012	0.08	True	True	0.623	0.277	True	True	carcinogen	low reliability	inactive	low reliability
PHE-44	-45.732	-30.69	-65.519	-20.35	-88.538	-36.53	0.996	-0.91	True	True	0.175	0.725	True	True	carcinogen	low reliability	inactive	low reliability
PHE-45	-42.210	-20.62	-57.363	-5.37	-78.756	-21.44	0.837	-0.75	True	True	1.000	-0.100	True	True	carcinogen	low reliability	inactive	moderate reliability
PHE-46	-41.751	-19.31	-56.409	-3.62	-82.603	-27.38	0.994	-0.91	True	True	1.000	-0.100	True	True	non-carcinogen	low reliability	active	moderate reliability
PHE-47	-43.643	-24.72	-62.993	-15.71	-86.656	-33.63	0.958	-0.87	True	True	0.002	0.898	True	True	non-carcinogen	low reliability	active	good reliability
PHE-48	-38.748	-10.73	-55.416	-1.79	-82.667	-27.47	0.995	-0.91	False	True	0.000	0.900	True	True	carcinogen	good reliability	active	good reliability
PHE-49	-40.406	-15.47	-57.622	-5.84	-81.118	-25.09	0.951	-0.86	True	True	0.000	0.900	True	True	non-carcinogen	low reliability	inactive	moderate reliability

PHE-50	-45.597	-30.30	-37.743	30.67	-69.596	-7.32	0.002	0.09	True	True	0.000	0.900	True	True	non-carcinogen	moderate reliability	inactive	good reliability
PHE-51	-44.613	-27.49	-30.543	43.90	-86.217	-32.95	0.003	0.09	True	True	0.000	0.900	True	True	non-carcinogen	good reliability	inactive	good reliability
PHE-52	-32.348	7.56	-59.670	-9.61	-88.119	-35.88	0.026	0.06	True	True	0.984	-0.084	True	True	carcinogen	moderate reliability	inactive	low reliability
PHE-53	-38.443	-9.86	-51.543	5.32	-74.099	-14.26	0.585	-0.50	True	True	0.980	-0.080	True	True	carcinogen	low reliability	inactive	moderate reliability
PHE-54	-30.288	13.45	-45.282	16.82	-46.368	28.50	0.009	0.08	True	True	1.000	-0.100	True	True	carcinogen	moderate reliability	inactive	moderate reliability
PHE-55	-25.874	26.06	-46.270	15.01	-70.818	-9.20	0.383	-0.29	True	True	1.000	-0.100	True	True	carcinogen	low reliability	inactive	low reliability
PHE-56	-47.571	-35.94	-70.941	-30.31	-55.174	14.92	0.863	-0.77	True	True	0.969	-0.069	True	True	non-carcinogen	low reliability	active	good reliability
**PYR**	-48.667	0.00	-92.176	0.00	-89.548	0.00	0.966	0.00	True	True	0.023	0.000	True	True	carcinogen	good reliability	inactive	low reliability
PYR-1	-66.502	-36.65	-84.850	7.95	-99.642	-11.27	0.876	0.09	True	True	1.000	-0.977	True	True	carcinogen	low reliability	inactive	moderate reliability
PYR-2	-54.766	-12.53	-74.898	18.74	-80.454	10.16	0.992	-0.03	True	True	0.941	-0.918	True	True	carcinogen	low reliability	active	good reliability
PYR-3	-63.629	-30.74	-87.547	5.02	-86.141	3.80	0.992	-0.03	True	True	0.941	-0.918	True	True	carcinogen	low reliability	active	good reliability
PYR-4	-32.974	32.25	-57.563	37.55	-64.955	27.46	0.150	0.82	True	True	1.000	-0.977	True	True	carcinogen	moderate reliability	inactive	moderate reliability
PYR-5	-31.184	35.92	-50.340	45.39	-64.499	27.97	0.134	0.83	True	True	0.518	-0.495	True	True	non-carcinogen	low reliability	inactive	moderate reliability

PYR-6	-38.662	20.56	-93.387	-1.31	-69.591	22.29	0.491	0.48	True	True	1.000	-0.977	True	True	carcinogen	moderate reliability	inactive	moderate reliability
PYR-7	-34.002	30.13	-57.512	37.61	-69.213	22.71	0.981	-0.02	True	True	0.999	-0.976	True	True	non-carcinogen	moderate reliability	inactive	moderate reliability
PYR-8	-59.322	-21.89	-35.555	61.43	-79.444	11.28	0.999	-0.03	False	False	0.000	0.023	True	True	carcinogen	good reliability	inactive	moderate reliability
PYR-9	-53.628	-10.19	-69.957	24.10	-103.368	-15.43	0.999	-0.03	False	False	0.000	0.023	True	True	carcinogen	good reliability	inactive	moderate reliability
PYR-10	-1.349	97.23	-3.929	95.74	-40.791	54.45	0.111	0.86	True	True	1.000	-0.977	True	True	non-carcinogen	low reliability	active	good reliability
PYR-11	-58.457	-20.12	-101.239	-9.83	-137.721	-53.80	0.978	-0.01	True	True	0.999	-0.976	True	True	non-carcinogen	low reliability	active	good reliability
PYR-12	-50.602	-3.98	-85.062	7.72	-100.029	-11.70	0.018	0.95	False	True	1.000	-0.977	True	True	non-carcinogen	low reliability	inactive	low reliability
PYR-13	-61.607	-26.59	-60.614	34.24	-84.059	6.13	—	—	—	—	—	—	—	—	carcinogen	good reliability	inactive	low reliability
PYR-14	-76.281	-56.74	-83.559	9.35	-104.092	-16.24	0.078	0.89	True	True	1.000	-0.977	True	True	non-carcinogen	low reliability	active	good reliability
PYR-15	-69.097	-41.98	-76.519	16.99	-102.862	-14.87	0.999	-0.03	True	True	1.000	-0.977	True	True	carcinogen	moderate reliability	active	good reliability
PYR-16	-49.438	-1.58	-68.013	26.21	-107.412	-19.95	1.000	-0.03	False	False	0.999	-0.976	True	True	carcinogen	low reliability	inactive	moderate reliability
PYR-17	-39.399	19.04	-59.086	35.90	-76.575	14.49	0.981	-0.02	True	True	0.999	-0.976	True	True	non-carcinogen	moderate reliability	inactive	moderate reliability
PYR-18	-56.798	-16.71	-59.490	35.46	-88.580	1.08	0.992	-0.03	True	True	0.832	-0.809	True	True	non-carcinogen	moderate reliability	inactive	low reliability

PYR-19	-49.510	-1.73	-73.736	20.01	-106.364	-18.78	0.680	0.29	True	True	0.000	0.023	False	False	carcinogen	moderate reliability	inactive	good reliability
PYR-20	-35.056	27.97	-44.128	52.13	-43.388	51.55	0.497	0.47	True	True	0.275	-0.252	True	True	carcinogen	low reliability	inactive	low reliability
PYR-21	-43.389	10.85	-87.016	5.60	-68.335	23.69	0.986	-0.02	True	True	0.000	0.023	True	True	carcinogen	low reliability	inactive	low reliability
PYR-22	-44.014	9.56	-79.650	13.59	-94.413	-5.43	0.976	-0.01	True	True	0.000	0.023	True	True	carcinogen	moderate reliability	inactive	low reliability
PYR-23	-31.488	35.30	-69.581	24.51	-78.530	12.30	0.973	-0.01	True	True	0.000	0.023	False	False	carcinogen	moderate reliability	inactive	good reliability
PYR-24	-36.163	25.69	-59.975	34.93	-79.842	10.84	0.974	-0.01	True	True	0.000	0.023	False	False	carcinogen	moderate reliability	inactive	low reliability
PYR-25	-43.258	11.11	-56.638	38.55	-108.076	-20.69	0.003	0.96	True	True	0.000	0.023	True	True	non-carcinogen	good reliability	inactive	good reliability
PYR-26	-28.117	42.23	-46.576	49.47	-67.123	25.04	0.363	0.60	True	True	1.000	-0.977	True	True	carcinogen	low reliability	inactive	low reliability
PYR-27	-28.739	40.95	-48.163	47.75	-61.458	31.37	0.527	0.44	True	True	0.999	-0.976	True	True	carcinogen	low reliability	inactive	moderate reliability
PYR-28	-30.195	37.96	-46.694	49.34	-60.176	32.80	0.127	0.84	True	True	0.116	-0.093	False	False	non-carcinogen	low reliability	inactive	low reliability
PYR-29	-33.046	32.10	-57.915	37.17	-65.114	27.29	0.150	0.82	True	True	1.000	-0.977	True	True	carcinogen	moderate reliability	inactive	moderate reliability
PYR-30	-38.579	20.73	-59.051	35.94	-64.679	27.77	0.000	0.97	True	True	0.980	-0.957	True	True	non-carcinogen	low reliability	inactive	moderate reliability
PYR-31	-62.461	-28.34	-104.646	-13.53	-125.473	-40.12	0.996	-0.03	True	True	1.000	-0.977	False	False	non-carcinogen	low reliability	inactive	moderate reliability

PYR-32	-42.680	12.30	-69.229	24.89	-51.141	42.89	0.022	0.94	True	True	1.000	-0.977	True	True	carcinogen	low reliability	inactive	low reliability
PYR-33	-37.317	23.32	-34.736	62.32	-83.727	6.50	0.720	0.25	True	True	1.000	-0.977	True	True	non-carcinogen	moderate reliability	inactive	moderate reliability
PYR-34	-53.347	-9.62	-93.198	-1.11	-101.108	-12.91	1.000	-0.03	True	True	0.997	-0.974	True	True	non-carcinogen	low reliability	inactive	good reliability
PYR-35	-48.660	0.01	-56.695	38.49	-62.765	29.91	0.129	0.84	True	True	0.999	-0.976	True	True	non-carcinogen	low reliability	active	good reliability
PYR-36	-41.335	15.07	-59.027	35.96	-76.073	15.05	0.250	0.72	True	True	1.000	-0.977	True	True	non-carcinogen	low reliability	active	moderate reliability
PYR-37	-35.386	27.29	-32.670	64.56	-56.987	36.36	0.097	0.87	True	True	0.998	-0.975	True	True	non-carcinogen	moderate reliability	inactive	low reliability
PYR-38	-57.385	-17.91	-79.740	13.49	-116.044	-29.59	0.013	0.95	True	True	1.000	-0.977	True	True	non-carcinogen	low reliability	inactive	moderate reliability
PYR-39	-26.501	45.55	-38.487	58.25	-54.964	38.62	0.106	0.86	True	True	0.004	0.019	True	True	carcinogen	moderate reliability	inactive	good reliability
PYR-40	-26.578	45.39	-33.558	63.59	-51.347	42.66	0.154	0.81	True	True	0.000	0.023	True	True	non-carcinogen	low reliability	inactive	low reliability

Note: [a] the molecule without skeleton (benzene ring) structure cannot be predicted by 3D-QSAR model; [b] the molecule failed in the model.

Table S4 Comparison of environmental and human health risks for PAHs metabolism and transformation pathways

Parent PAH	Pathway	PAH derivatives	Neurotoxicity		Immunotoxicity		Phytotoxicity		Developmental toxicity		Genotoxicity		Carcinogenicity	Endocrine disrupting effect
			Binding free energy (kJ/mol)	Toxicity reduction intensity (%)	Binding free energy (kJ/mol)	Toxicity reduction intensity (%)	Binding free energy (kJ/mol)	Toxicity reduction intensity (%)	Developmental toxicity potential	Toxicity reduction range	Ames mutagenicity	Toxicity reduction range		
**ACE**	All	ACE derivatives	-49.724— -26.776	-53.72—17.22	-72.418— -14.795	-5.12— 78.52	-123.595— -59.313	-124.88— -7.92	0.001— 0.996	0.00— 1.00	0.000— 1.000	-0.88— 0.12	carcinogen	active
	Microbial (bacteria) degradation	ACE-1—ACE-17	-49.724— -26.776	-53.72—17.22	-72.418— -14.795	-5.12—78.52	-123.595— -59.313	-124.88— -7.92	0.001— 0.996	0.00— 1.00	0.000— 1.000	-0.88— 0.12	carcinogen	active
	Photolysis	ACE-1	-30.863	4.59	-72.418	-5.12	-65.769	-19.66	0.702	0.30	0.999	-0.88	non-carcinogen	inactive
**ACY**	All	ACY derivatives	-36.556— -14.249	21.47—69.39	-72.988— -31.346	-37.08— 41.13	-122.813— -55.831	-130.58— -4.82	0.001—0.996	0.00—1.00	0.000—1.000	-0.49— 0.52	non-carcinogen	inactive
	Microbial (bacteria) degradation	ACY-1—ACY-8	-36.556— -23.973	21.47—48.50	-68.571— -31.346	-28.79— 41.13	-98.245— -55.831	-84.45— -4.82	0.001—0.607	0.39— 1.00	0.000— 1.000	-0.49— 0.52	non-carcinogen	inactive
	Photolysis	ACY-9—ACY-10	-28.381— -14.249	39.03—69.39	-72.988— -59.479	-37.08— -11.71	-122.813— -66.710	-130.58— -25.25	0.024—0.996	0.00—0.97	0.000—1.000	-0.49— 0.52	non-carcinogen	inactive
**ANT**	All	ANT derivatives	-55.954— -11.525	-27.41— 73.76	-96.738— -29.651	-38.96— 57.41	-120.143— -27.961	-85.26— 56.88	0.000—1.000	-0.47— 0.54	0.000— 1.000	-1.00— 0.00	carcinogen	active
	Photocatalytic degradation	ANT-1—ANT-3	-42.833— -34.555	2.46— 21.31	-93.540— -80.937	-34.36— -16.26	-101.756—-87.551	-56.90— -35.00	0.359—0.966	-0.43—0.18	0.000—0.926	-0.93—0.00	carcinogen	inactive
	Microbial (fungus) degradation	ANT-4—ANT-11	-51.064— -28.192	-16.28— 35.80	-96.738— -29.651	-38.96— 57.41	-113.856— -27.961	-75.56— 56.88	0.001— 0.383	0.15— 0.53	0.000— 1.000	-1.00— 0.00	carcinogen	inactive
	Microbial (bacteria) degradation	ANT-12—ANT-22	-55.954— -11.525	-27.41— 73.76	-94.515— -33.558	-35.76— 51.80	-120.143— -51.347	-85.26— -20.82	0.000—1.000	-0.47— 0.54	0.000— 1.000	-1.00— 0.00	carcinogen	active
**BaAN**	All	BaAN derivatives	-67.321— -14.788	-21.30—73.36	-115.276— -25.966	-25.38— 71.76	-143.955—-40.669	-34.75— 61.93	0.000— 1.000	-0.50— 0.50	0.000— 1.000	0.00— 1.00	carcinogen	active
	Human metabolism	BaAN-1—BaAN-11	-51.011— -29.036	8.09— 47.68	-88.986— -42.767	3.21— 53.48	-88.281— -55.540	17.37— 48.01	0.953— 1.000	-0.50— -0.46	1.000	0.00	carcinogen	inactive
	Biological (chick embryos) metabolism	BaAN-12	-55.764	-0.47	-115.276	-25.38	-117.772	-10.24	0.473	0.02	1.000	0.00	carcinogen	inactive
	Photolysis	BaAN-12	-55.764	-0.47	-115.276	-25.38	-117.772	-10.24	0.473	0.02	1.000	0.00	carcinogen	inactive
	Microbial (microalgae) degradation	BaAN-13—BaAN-15	-48.064— -34.754	13.40—37.38	-77.284— -52.225	15.94— 43.20	-64.851— -40.669	39.30—61.93	0.000— 0.535	-0.04— 0.50	0.000— 1.000	0.00— 1.00	carcinogen	active
	Microbial (fungi)	BaAN-16—BaAN-25	-67.321— -14.788	-21.30— 73.36	-77.176— -25.966	16.06—71.76	-123.041— -60.790	-15.17— 43.10	0.003— 1.000	-0.50— 0.49	0.000— 1.000	0.00— 1.00	carcinogen	inactive

	degradation													
	Photocatalytic degradation	BaAN-20, BaAN-22, BaAN-26—BaAN-30	-50.880— -25.556	8.33— 53.95	-98.335— -43.963	-6.96—52.18	-143.955— -68.091	-34.75— 36.26	0.001— 0.864	-0.37— 0.50	0.000— 1.000	0.00— 1.00	carcinogen	active
**BaP**	All	BaP derivatives	-84.016— -20.605	-43.05— 64.92	-125.188— -14.879	-47.57—82.46	-141.796— -32.779	-80.77— 58.21	0.000— 1.000	-0.02— 0.98	0.000— 1.000	0.00— 1.00	carcinogen	active
	Human metabolism	BaP-1	-58.469	0.45	-101.226	-19.32	-48.984	37.55	0.996	-0.02	1.000	0.00	carcinogen	active
	Biological (fish) metabolism	BaP-1	-58.469	0.45	-101.226	-19.32	-48.984	37.55	0.996	-0.02	1.000	0.00	carcinogen	active
	Photolysis	BaP-2—BaP-15	-69.026— -23.614	-17.53— 59.79	-97.378— -26.098	-14.79— 69.24	-118.813— -53.228	-51.47—32.14	0.000— 1.000	-0.02— 0.98	0.000— 1.000	0.00— 1.00	carcinogen	active
	Photocatalytic degradation	BaP-16—BaP-26	-84.016— -38.847	-43.05— 33.86	-125.188— -53.420	-47.57— 37.03	-138.780— -32.779	-76.92— 58.21	0.966— 1.000	-0.02— 0.01	0.004— 1.000	0.00— 1.00	carcinogen	active
	Resistance of plants	BaP-1, BaP-27—BaP-30	-69.585— -34.162	-18.48—41.83	-105.899— -54.486	-24.83— 35.77	-72.477— -46.346	7.60—40.92	0.996— 0.999	-0.02	1.000	0.00	carcinogen	active
	Microbial (fungi) degradation	BaP-1, BaP-31—BaP-32	-69.429— -40.143	-18.21— 31.65	-101.226— -52.613	-19.32—37.98	-80.492— -48.984	-2.61—37.55	0.996— 0.999	-0.02	1.000	0.00	carcinogen	active
	Anaerobic biodegradation	BaP-33—BaP-41	-68.631— -32.072	-16.85— 45.39	-73.949— -26.064	12.83— 69.28	-96.907— -46.446	-23.54—40.79	0.006— 0.150	0.83— 0.97	0.000— 1.000	0.00— 1.00	carcinogen	inactive
	Microbial (bacteria) degradation	BaP-42—BaP-72	-74.787— -20.605	-27.34— 64.92	-125.057—-14.879	-47.41— 82.46	-141.796— -34.612	-80.77— 55.88	0.000— 1.000	-0.02— 0.98	0.000— 1.000	0.00— 1.00	carcinogen	active
	Microbial (microalgae) degradation	BaP-73—BaP-76	-54.468— -34.878	7.26— 40.61	-100.420— -58.329	-18.37— 31.24	-72.028— -49.357	8.18— 37.08	0.999	-0.02	1.000	0.00	carcinogen	active
**BbF**	All	BbF derivatives	-59.001— -38.117	-32.99— 14.08	-116.554— -68.560	-12.35— 33.91	-123.242— -38.920	-22.84— 61.21	0.074— 0.999	-0.02— 0.90	0.000— 1.000	0.00— 1.00	carcinogen	active
	Biological (mouse) metabolism	BbF-1—BbF-7	-50.581— -43.551	-14.01— 1.83	-111.757— -85.228	-7.73—17.85	-112.345— -47.563	-11.97— 52.29	0.996— 0.999	-0.02	1.000	0.00	carcinogen	active
	Human metabolism	BbF-7—BbF-10	-59.001— -47.020	-32.99— -5.98	-116.554— -91.181	-12.35— 12.11	-87.610— -43.364	12.68— 56.78	0.996	-0.02	1.000	0.00	carcinogen	active
	Microbial (bacteria) degradation	BbF-11—BbF-14	-54.891— -38.117	-23.73— 14.08	-106.386— -73.867	-2.55— 28.80	-104.155— -47.865	-3.81— 52.29	0.074— 0.941	0.04— 0.90	0.000— 1.000	0.00— 1.00	carcinogen	inactive
	Microbial (microalgae) degradation	BbF-15—BbF-17	-48.037— -41.990	-8.28— 5.35	-84.632— -68.560	18.42— 33.91	-123.242— -38.920	-22.84— 61.21	0.156— 0.158	0.82	1.000	0.00	carcinogen	active
**BghiP**	All	BghiP derivatives	-447.297— -26.335	-593.91—59.15	-939.422— -43.473	-674.54— 64.16	-618.118— -47.826	-527.74— 51.43	0.000— 1.000	0.00— 1.00	0.000— 1.000	0.00— 1.00	carcinogen	active
	Microbial (fungi)	BghiP-1—BghiP-4	-47.646— -26.335	26.08— 59.15	-76.430— -56.385	36.98—53.51	-127.607— -54.909	-29.59— 44.24	0.000— 1.000	0.00— 1.00	0.000— 1.000	0.00— 1.00	carcinogen	active

	degradation													
	Biological (rat) metabolism	BghiP-5—BghiP-17	-447.297— -42.194	-593.91— 34.54	-939.422— -43.473	-674.54— 64.16	-618.118— -47.826	-527.74— 51.43	0.998— 1.000	0.00	0.000— 1.000	0.00— 1.00	carcinogen	active
**BkF**	All	BkF derivatives	-393.264— -26.637	-673.50— 47.61	-728.263— -48.227	-548.08— 57.08	-69.994— 33.417	13.73— 141.19	0.019— 0.999	-0.01— 0.97	0.000— 1.000	0.00— 1.00	carcinogen	active
	Biological (rat) metabolism	BkF-1—BkF-3	-60.108— -48.353	-18.23— 4.90	-113.340— -92.277	-0.86—17.88	-69.994— -52.012	13.73— 35.89	0.996	-0.01	1.000	0.00	carcinogen	active
	Human metabolism	BkF-1—BkF-2	-60.108— -48.859	-18.23— 3.90	-113.340— -92.277	-0.86— 17.88	-69.994— -63.227	13.73— 22.07	0.996	-0.01	1.000	0.00	carcinogen	active
	Microbial (microalgae) degradation	BkF-4	-35.219	30.73	-66.048	41.22	-46.886	42.21	0.999	-0.01	1.000	0.00	carcinogen	active
	Microbial (bacteria) degradation	BkF-5—BkF-18	-393.264— -26.637	-673.50— 47.61	-728.263— -48.227	-548.08— 57.08	-50.362— 33.417	37.93— 141.19	0.019— 0.999	-0.01— 0.97	0.000— 1.000	0.00— 1.00	carcinogen	active
**CHR**	All	CHR derivatives	-42.028— -13.018	36.60— 80.36	-86.488— -15.457	-3.91— 81.43	-119.656— -57.965	-42.15— 31.14	0.001— 0.961	-0.24— 0.72	0.000— 1.000	0.00— 1.00	carcinogen	active
	Biological (fish) metabolism	CHR-1—CHR-2	-42.028— -30.392	36.60— 54.15	-73.514— -41.395	11.68— 50.27	-95.598— -57.965	-13.57— 31.14	0.006	0.71	0.979— 0.998	0.00	carcinogen	active
	Human metabolism	CHR-3	-39.502	40.41	-86.488	-3.91	-85.910	-2.06	0.864	-0.14	1.000	0.00	carcinogen	active
	Microbial (bacteria) degradation	CHR-4—CHR-9	-39.408— -13.018	40.55— 80.36	-72.113— -15.457	13.36— 81.43	-119.656— -63.309	-42.15— 24.79	0.001— 0.473	0.25— 0.72	0.000— 1.000	0.00— 1.00	carcinogen	active
	Microbial (fungi) degradation	CHR-5—CHR-6, CHR-9—CHR-11	-36.788— -13.018	44.50— 80.36	-74.395— -15.457	10.62— 81.43	-119.656— -64.113	-42.15— 23.83	0.001— 0.961	-0.24— 0.72	0.000— 1.000	0.00— 1.00	carcinogen	active
**DahA**	All	DahA derivatives	-57.132— -39.852	14.06— 40.05	-98.078— -42.755	6.82— 59.38	-136.040— -62.555	-66.66— 23.36	0.000— 1.000	-0.01—0.99	0.000— 1.000	0.00— 1.00	carcinogen	active
	Biological (rat) metabolism	DahA-1	-57.132	14.06	-98.078	6.82	-62.555	23.36	0.996	-0.01	1.000	0.00	carcinogen	active
	Microbial (fungi) degradation	DahA-2—DahA-7	-56.130— -39.852	15.57— 40.05	-96.642— -42.755	8.18— 59.38	-136.040— -66.963	-66.66— 17.96	0.000— 1.000	-0.01—0.99	0.000— 1.000	0.00— 1.00	carcinogen	inactive
**FLR**	All	FLR derivatives	-52.982— -17.263	-28.03— 58.28	-96.459— -19.218	-43.07— 71.50	-123.091— -54.768	-47.47— 34.39	0.001— 1.000	0.00— 1.00	0.000— 1.000	-0.51—0.49	carcinogen	active
	Biological (bovine) metabolism	FLR-1—FLR-3	-42.987— -31.152	-3.88— 24.72	-87.784— -75.723	-30.20— -12.31	-68.285— -54.768	18.19— 34.39	0.089— 0.999	0.00— 0.91	0.930— 0.997	-0.50— -0.44	carcinogen	active
	Human metabolism	FLR-1—FLR-3	-42.987— -31.152	-3.88— 24.72	-87.784— -75.723	-30.20— -12.31	-68.285— -54.768	18.19— 34.39	0.089— 0.999	0.00— 0.91	0.930— 0.997	-0.50— -0.44	carcinogen	active
	Biological (fish) metabolism	FLR-1—FLR-3	-42.987— -31.152	-3.88— 24.72	-87.784— -75.723	-30.20— -12.31	-68.285— -54.768	18.19— 34.39	0.089— 0.999	0.00— 0.91	0.930— 0.997	-0.50— -0.44	carcinogen	active
	Microbial (bacteria)	FLR-3—FLR-27	-52.982— -17.263	-28.03— 58.28	-96.459— -19.218	-43.07— 71.50	-123.091— -57.079	-47.47— 31.62	0.001— 1.000	0.00— 1.00	0.000— 1.000	-0.51—0.49	carcinogen	active

	degradation													
	Anaerobic biodegradation	FLR-28	-25.304	38.85	-39.536	41.36	-83.491	-0.03	0.004	1.00	0.000	0.49	non-carcinogen	inactive
	Microbial (fungi) degradation	FLR-3, FLR-10	-34.156— -33.267	17.46— 19.61	-91.657— -87.784	-35.94— -30.20	-92.100— -62.063	-10.34— 25.65	0.089	0.91	0.930— 1.000	-0.51— -0.44	non-carcinogen	inactive
	Microbial (microalgae) degradation	FLR-1—FLR-3, FLR-10	-42.987— -31.152	-3.88— 24.72	-91.657— -75.723	-35.94— -12.31	-92.100— -54.768	-10.34— 34.39	0.089— 0.999	0.00— 0.91	0.930— 1.000	-0.51— -0.44	carcinogen	active
**FRT**	All	FRT derivatives	-82.714— -15.194	-68.66— 69.02	-122.943—-35.199	-17.95— 66.23	-128.579—262.389	-67.46—441.74	0.000— 1.000	-0.23— 0.77	0.000— 1.000	-0.00— 1.00	carcinogen	active
	Biological (fish) metabolism	FRT-1—FRT-11	-82.714— -31.478	-68.66—35.81	-122.943— -66.938	-17.95— 35.78	-72.433—41.159	5.66—153.61	0.876— 1.000	-0.23— -0.11	0.000— 1.000	-0.00— 1.00	carcinogen	inactive
	Human metabolism	FRT-3	-35.795	27.01	-80.005	23.24	-46.241	39.77	0.876	-0.11	1.000	-0.00	carcinogen	inactive
	Microbial (bacteria) degradation	FRT-12—FRT-45	-76.605— -27.094	-56.20— 44.75	-109.085— -35.199	-4.66— 66.23	-128.579— -41.565	-67.46— 45.86	0.000— 1.000	-0.23— 0.77	0.000— 1.000	-0.00— 1.00	carcinogen	active
	Microbial (fungi) degradation	FRT-46—FRT-54	-68.056— -15.194	-38.77— 69.02	-100.551— -43.963	3.53— 57.82	-102.801— 262.389	-33.89— 441.74	0.000— 0.998	-0.23— 0.77	0.000— 0.926	0.072— 0.998	carcinogen	active
	Microbial (microalgae) degradation	FRT-3—FRT-4	-35.795— -34.371	27.01— 29.92	-80.005— -69.047	23.24— 33.76	-46.341— -46.241	39.64— 39.77	0.876— 0.978	-0.21— -0.11	0.998— 1.000	-0.00	carcinogen	inactive
**IcdP**	All	IcdP derivatives	-70.988— -25.304	-9.09— 61.11	-104.145—-33.558	-26.39— 59.27	-114.025—-32.525	-5.44— 69.92	0.000— 1.000	0.00— 1.00	0.000— 1.000	0.00— 1.00	carcinogen	active
	Biological (rat) metabolism	IcdP-1—IcdP-3	-50.829— -40.223	21.89— 38.19	-96.162— -94.200	-16.70— -14.32	-64.579— -32.525	40.28— 69.92	1.000	0.00	1.000	0.00	carcinogen	active
	Biological (mouse) metabolism	IcdP-3—IcdP-9	-56.887— -34.753	12.58— 46.59	-100.539— -76.796	-22.02—6.80	-64.579— -47.806	40.28— 55.79	0.999— 1.000	0.00	1.000	0.00	carcinogen	active
	Human metabolism	IcdP-1	-40.223	38.19	-94.200	-14.32	-32.525	69.92	1.000	0.00	1.000	0.00	carcinogen	active
	Microbial (fungi) degradation	IcdP-10—IcdP-18	-70.988— -25.304	-9.09— 61.11	-92.749— -39.537	-12.56— 52.02	-114.025— -52.358	-5.44—51.58	0.000— 1.000	0.00— 1.00	0.000— 0.998	0.00— 1.00	carcinogen	active
	Microbial (bacteria) degradation	IcdP-19—IcdP-35	-53.863— -25.438	17.22— 60.91	-99.835— -33.558	-21.16— 59.27	-105.571— -45.118	2.38— 58.28	0.003— 1.000	0.00— 1.00	0.000— 1.000	0.00— 1.00	carcinogen	active
	Biological (materials) metabolism	IcdP-2—IcdP-3, IcdP-5, IcdP-36—IcdP-37	-50.829— -37.259	21.89— 42.74	-104.145— -84.860	-26.39— -2.99	-64.579— -47.323	40.28— 56.24	1.000	0.00	1.000	0.00	carcinogen	active

NAP	All	NAP derivatives	-59.247— -16.318	-124.97—38.04	-122.487—-19.419	-262.36—42.55	-139.516—-47.258	-170.19—8.48	0.000— 1.000	-0.85— 0.15	0.000— 1.000	-1.00— 0.00	carcinogen	active
	Human metabolism	NAP-1—NAP-12	-50.880— -16.318	-93.20— 38.04	-73.514— -30.973	-117.48— 8.37	-97.538— -47.258	-88.89— 8.48	0.000— 1.000	-0.85— 0.15	0.000— 1.000	-1.00— 0.00	carcinogen	active
	Biological (mouse) metabolism	NAP-6—NAP-7, NAP-13—NAP-14	-43.902— -19.040	-66.70— 27.70	-74.199— -38.888	-119.50— -15.04	-88.619— -47.258	-71.62— 8.48	0.010— 1.000	-0.85— 0.14	0.000— 0.999	-1.00— 0.00	carcinogen	inactive
	Biological (fish) metabolism	NAP-4, NAP-8	-42.028— -30.392	-59. 58— -15.40	-73.514— -41.395	-117.48— -22.46	-95.597— -57.965	-85.13— -12.25	0.006	0.15	0.979— 0.998	-1.00— -0.98	carcinogen	active
	Biological (bovine) metabolism	NAP-4, NAP-8	-42.028— -30.392	-59. 58— -15.40	-73.514— -41.395	-117.48— -22.46	-95.597— -57.965	-85.13— -12.25	0.006	0.15	0.979— 0.998	-1.00— -0.98	carcinogen	active
	Anaerobic biodegradation	NAP-8, NAP-15—NAP-22	-59.247— -32.001	-124.97— -21.51	-122.487— -59.140	-262.36— -74.95	-139.516— -56.459	-170.19— -9.34	0.000— 0.954	-0.80— 0.15	0.000— 0.999	-1.00— 0.00	non-carcinogen	active
	Microbial (bacteria) degradation	NAP-3, NAP-23—NAP-31	-57.386— -19.000	-117.90— 27.86	-79.740— -19.419	-135.90— 42.55	-120.144— -61.195	-132.67— -18.51	0.000— 0.034	0.12— 0.15	0.000— 1.000	-1.00— 0.00	carcinogen	active
	Microbial (microalgae) degradation	NAP-1, NAP-4, NAP-23, NAP-32	-40.491— -25.790	-53.75— 2.07	-58.844— -30.973	-74.08— 8.37	-82.947— -52.474	-60.63— -1.62	0.000— 0.228	-0.07— 0.15	0.000— 1.000	-1.00— 0.00	carcinogen	inactive
PHE	All	PHE derivatives	-62.817— -9.317	-79.51—73.37	-96.460—37.312	-77.19—168.54	-123.326—-40.193	-90.17— 38.02	0.000— 0.996	-0.91— 0.09	0.001— 1.000	-0.10— 0.90	carcinogen	active
	Biological (marine teleosts) metabolism	PHE-1—PHE-2	-35.758— -31.814	-2.19— 9.08	-69.663— -66.886	-27.96— -22.86	-92.600— -76.213	-42.79— -17.52	0.150	-0.06	0.999	-0.10	carcinogen	inactive
	Biological (bovine) metabolism	PHE-1—PHE-5	-40.942— -31.814	-17.00— 9.08	-69.663— -49.665	-27.96— 8.77	-92.600— -54.703	-42.79— 15.65	0.150	-0.06	0.998— 1.000	-0.10	carcinogen	active
	Human metabolism	PHE-1, PHE-3—PHE-5	-40.942— -31.814	-17.00— 9.08	-66.886— -49.665	-22.86— 8.77	-92.600— -54.703	-42.79— 15.65	0.150	-0.06	0.998— 1.000	-0.10	carcinogen	active
	Photolysis	PHE-6—PHE-9	-62.817— -33.267	-79.51— 4.93	-96.460— -82.981	-77.19— -52.43	-123.326— -86.445	-90.17— -33.30	0.067— 0.630	-0.54— 0.02	0.001— 1.000	-0.10— 0.90	carcinogen	inactive
	Microbial (bacteria) degradation	PHE-10—PHE-41	-54.812— -9.317	-56.64— 73.37	-84.386— 37.312	-55.01— 168.54	-123.002— -40.193	-89.67— 38.02	0.000— 0.585	-0.50— 0.09	0.000— 1.000	-0.10— 0.90	carcinogen	active
	Coupled photocatalysis and biodegradation	PHE-2, PHE-6, PHE-9, PHE-42	-62.817— -35.758	-79.51— -2.19	-89.957— -69.663	-65.24— -27.96	-123.326— -76.213	-90.17— -17.52	0.067— 0.766	-0.68— 0.02	0.001— 1.000	-0.10— 0.90	carcinogen	inactive
	Microbial (microalgae) degradation	PHE-18, PHE-43—PHE-49	-49.177— -35.969	-40.53— -2.79	-73.250— -55.416	-34.55— -1.79	-98.149— -70.519	-51.35— -8.74	0.012— 0.996	-0.91— 0.08	0.000— 1.000	-0.10— 0.90	carcinogen	active

	Coupled plant and microbial catabolisms	PHE-1—PHE-2, PHE-9, PHE-25, PHE-33, PHE-50—PHE-51	-62.817— -30.683	-79.51— 12.32	-66.886— -30.543	-22.86— 43.90	-123.326— -59.819	-90.17— 7.76	0.001— 0.150	-0.06— 0.09	0.000— 1.000	-0.10— 0.90	carcinogen	active
	Microbial (fungi) degradation	PHE-52—PHE-56	-47.571— -25.874	-35.94— 26.06	-70.941— -45.282	-30.31— 16.82	-88.119— -46.368	-35.88— 28.50	0.009— 0.863	-0.77— 0.08	0.969— 1.000	-0.10— -0.07	carcinogen	active
**PYR**	All	PYR derivatives	-76.281— -1.349	-56.74— 97.23	-104.646— -3.929	-13.53— 95.74	-137.721— -40.791	-53.80— 54.45	0.000— 1.000	-0.03— 0.97	0.000— 1.000	-0.98— 0.023	carcinogen	active
	Biological (bovine) metabolism	PYR-1	-66.502	-36.65	-84.850	7.95	-99.642	-11.27	0.876	0.09	1.000	-0.98	carcinogen	inactive
	Biological (fish) metabolism	PYR-1	-66.502	-36.65	-84.850	7.95	-99.642	-11.27	0.876	0.09	1.000	-0.98	carcinogen	inactive
	Human metabolism	PYR-1—PYR-3	-66.502— -54.766	-36.65— -12.53	-87.547— -74.898	5.02— 18.74	-99.642— -80.454	-11.27— 10.16	0.876— 0.992	-0.03— 0.09	0.941— 1.000	-0.99— -0.92	carcinogen	active
	Resistance of plants	PYR-1, PYR-3—PYR-5	-66.502— -31.184	-36.65— 35.92	-87.547— -50.340	5.02— 45.39	-99.642— -64.499	-11.27— 27.97	0.134— 0.992	-0.03— 0.83	0.518— 1.000	-0.98— -0.50	carcinogen	active
	Microbial (fungi) degradation	PYR-1, PYR-6—PYR-9	-66.502— -34.002	-36.65— 30.13	-93.387— -35.555	-1.31— 61.43	-103.368— -69.213	-15.43— 22.71	0.491— 0.999	-0.03— 0.48	0.000— 1.000	-0.98— 0.02	carcinogen	inactive
	Microbial (bacteria) degradation	PYR-5, PYR-10—PYR-34	-76.281— -1.349	-56.74— 97.23	-104.646— -3.929	-13.53— 95.74	-137.721— -40.791	-53.80— 54.45	0.000— 1.000	-0.03— 0.97	0.000— 1.000	-0.98— 0.023	carcinogen	active
	Photocatalytic degradation	PYR-1, PYR-6, PYR-19, PYR-25—PYR-26, PYR-35—PYR-40	-66.502— -26.501	-36.65— 45.55	-93.387— -32.670	-1.31— 64.56	-116.044— -51.347	-29.59— 42.66	0.003—0.876	0.09— 0.96	0.000— 1.000	-0.98— 0.02	carcinogen	active
	Microbial (microalgae) degradation	PYR-1	-66.502	-36.65	-84.850	7.95	-99.642	-11.27	0.876	0.09	1.000	-0.98	carcinogen	inactive

Table S5 Prediction and evaluation of bioenrichment and persistence for PAHs derivatives

PAH and its derivatives	bioenrichment		persistence								
	BCF model (CAESAR)		kM/Half-Life model			Persistence (soil) quantitative model			Air Half-Life (IRFMN/CORAL)		
	BCF [log(L/kg)]	Assessment reliability	kM/Half-Life	Assessment reliability	Toxicity reduction rage	Persistence (day)	Assessment reliability	Toxicity reduction rage	Prediction [log(hours)]	Assessment reliability	Toxicity reduction rage
ACE	2.88	experimental value	-0.41	moderate reliability	0.00	229	experimental value	0.00	-1.740	experimental value	0.00
ACE-1	1.10	low reliability	-1.30	moderate reliability	87.12	5	low reliability	97.82	-1.873	moderate reliability	26.38
ACE-2	0.63	low reliability	-2.14	low reliability	98.14	7	low reliability	96.94	-1.993	low reliability	44.15
ACE-3	0.63	low reliability	-2.14	low reliability	98.14	7	low reliability	96.94	-1.993	low reliability	44.15
ACE-4	0.63	low reliability	-2.14	low reliability	98.14	7	low reliability	96.94	-1.993	low reliability	44.15
ACE-5	1.06	low reliability	-0.91	moderate reliability	68.38	41	moderate reliability	82.10	-1.615	moderate reliability	-33.35
ACE-6	0.60	low reliability	-1.87	moderate reliability	96.53	17	low reliability	92.58	-1.736	moderate reliability	-0.93
ACE-7	0.57	low reliability	-1.32	moderate reliability	87.70	34	low reliability	85.15	-1.494	low reliability	-76.20
ACE-8	0.78	good reliability	-0.44	moderate reliability	6.67	5	moderate reliability	97.82	-2.284	moderate reliability	71.42
ACE-9	1.03	good reliability	-2.22	low reliability	98.45	41	moderate reliability	82.10	-1.649	moderate reliability	-23.31
ACE-10	1.10	good reliability	0.15	low reliability	-263.08	7	good reliability	96.94	-1.350	good reliability	-145.47
ACE-11	0.25	low reliability	-1.70	low reliability	94.87	8	low reliability	96.51	-0.943	low reliability	-526.61
ACE-12	0.46	good reliability	-1.82	moderate reliability	96.11	7	moderate reliability	96.94	-1.467	moderate reliability	-87.50
ACE-13	0.09	low reliability	-0.74	low reliability	53.23	16	low reliability	93.01	-3.030	low reliability	94.87
ACE-14	0.16	low reliability	-0.69	moderate reliability	47.52	5	moderate reliability	97.82	-2.680	moderate reliability	88.52
ACE-15	0.14	moderate reliability	-0.63	moderate reliability	39.74	5	good reliability	97.82	-2.664	moderate reliability	88.09
ACE-16	0.34	good reliability	-1.09	moderate reliability	79.11	7	experimental value	96.94	-1.731	experimental value	-2.09
ACE-17	0.23	low reliability	-1.86	low reliability	96.45	7	experimental value	96.94	-1.476	experimental value	-83.65
ACY	2.11	experimental value	0.52	experimental value	0.00	225	moderate reliability	0.00	-1.675	low reliability	0.00
ACY-1	0.63	low reliability	-2.14	low reliability	99.78	7	low reliability	96.89	-1.993	low reliability	-18.99

ACY-2	0.60	low reliability	-1.87	moderate reliability	99.59	17	low reliability	92.44	-1.736	moderate reliability	-3.64
ACY-3	0.63	low reliability	-0.86	moderate reliability	95.83	7	low reliability	96.89	-1.467	low reliability	12.42
ACY-4	0.57	low reliability	-1.32	moderate reliability	98.55	34	low reliability	84.89	-1.494	low reliability	10.81
ACY-5	0.78	good reliability	-0.44	moderate reliability	89.04	5	moderate reliability	97.78	-2.284	moderate reliability	-36.36
ACY-6	1.10	good reliability	0.15	low reliability	57.34	7	good reliability	96.89	-1.350	good reliability	19.40
ACY-7	0.34	good reliability	-1.09	moderate reliability	97.55	7	experimental value	96.89	-1.731	experimental value	-3.34
ACY-8	0.11	moderate reliability	-2.03	low reliability	99.72	5	good reliability	97.78	-1.735	moderate reliability	-3.58
ACY-9	1.06	low reliability	-0.91	moderate reliability	96.28	41	moderate reliability	81.78	-1.615	moderate reliability	3.58
ACY-10	0.96	good reliability	-0.44	moderate reliability	89.04	5	moderate reliability	97.78	-1.917	moderate reliability	-14.45
ANT	2.99	experimental value	0.65	experimental value	0.00	229	experimental value	0.00	-1.740	experimental value	0.00
ANT-1	1.91	moderate reliability	-0.56	moderate reliability	93.83	5	moderate reliability	97.82	-1.686	moderate reliability	-13.24
ANT-2	1.62	moderate reliability	-1.48	low reliability	99.26	5	moderate reliability	97.82	-1.650	moderate reliability	-23.03
ANT-3	1.40	low reliability	-1.49	moderate reliability	99.28	5	low reliability	97.82	-1.368	low reliability	-135.50
ANT-4	1.63	low reliability	0.25	low reliability	60.19	41	low reliability	82.10	-2.421	low reliability	79.16
ANT-5	1.13	low reliability	-0.93	moderate reliability	97.37	17	low reliability	92.58	-2.069	low reliability	53.12
ANT-6	1.13	low reliability	-0.41	moderate reliability	91.29	41	low reliability	82.10	-1.667	low reliability	-18.30
ANT-7	0.52	moderate reliability	-2.48	low reliability	99.93	13	good reliability	94.32	-1.518	moderate reliability	-66.72
ANT-8	0.25	low reliability	-0.61	moderate reliability	94.50	5	moderate reliability	97.82	-2.555	moderate reliability	84.69
ANT-9	0.33	good reliability	-1.22	moderate reliability	98.65	5	good reliability	97.82	-2.539	experimental value	84.11
ANT-10	0.62	low reliability	-1.04	moderate reliability	97.96	7	experimental value	96.94	-1.647	experimental value	-23.88
ANT-11	0.23	low reliability	-1.86	low reliability	99.69	7	experimental value	96.94	-1.476	experimental value	-83.65
ANT-12	1.81	low reliability	0.00	low reliability	77.61	41	low reliability	82.10	-1.507	low reliability	-71.00
ANT-13	1.59	moderate reliability	-0.69	moderate reliability	95.43	5	low reliability	97.82	-2.006	moderate reliability	45.80
ANT-14	2.51	good reliability	-0.21	moderate reliability	86.20	23	moderate reliability	89.96	-0.346	moderate reliability	-2377.42
ANT-15	2.60	low reliability	1.38	low reliability	-437.03	41	low reliability	82.10	-1.404	low reliability	-116.77
ANT-16	0.80	low reliability	-0.81	moderate reliability	96.53	2	experimental value	99.13	-1.663	experimental value	-19.40

ANT-17	0.52	moderate reliability	-1.50	low reliability	99.29	2	good reliability	99.13	-1.392	experimental value	-122.84
ANT-18	0.60	moderate reliability	-0.39	moderate reliability	90.88	5	moderate reliability	97.82	-2.394	moderate reliability	77.82
ANT-19	3.12	moderate reliability	0.33	good reliability	52.14	229	good reliability	0.00	-1.642	good reliability	-25.31
ANT-20	2.50	experimental value	0.41	experimental value	42.46	71	experimental value	69.00	-1.652	experimental value	-22.46
ANT-21	0.58	low reliability	-0.80	moderate reliability	96.45	34	low reliability	85.15	-1.828	low reliability	18.34
ANT-22	0.75	low reliability	-1.22	moderate reliability	98.65	13	low reliability	94.32	-0.788	low reliability	-795.36
BaAN	2.54	experimental value	0.02	experimental value	0.00	708	experimental value	0.00	-2.230	experimental value	0.00
BaAN-1	1.78	low reliability	-1.82	low reliability	98.55	8	low reliability	98.87	-3.151	low reliability	88.01
BaAN-2	1.82	low reliability	-1.36	low reliability	95.83	94	low reliability	86.72	-3.281	low reliability	91.11
BaAN-3	1.82	low reliability	-1.36	low reliability	95.83	94	low reliability	86.72	0.015	low reliability	-17479.24
BaAN-4	1.81	low reliability	-1.36	low reliability	95.83	94	low reliability	86.72	-1.996	low reliability	-71.40
BaAN-5	1.82	low reliability	-1.36	low reliability	95.83	94	low reliability	86.72	-1.857	low reliability	-136.05
BaAN-6	1.00	low reliability	-1.44	moderate reliability	96.53	34	low reliability	95.20	-1.098	low reliability	-1255.19
BaAN-7	1.04	low reliability	-1.44	moderate reliability	96.53	34	low reliability	95.20	-1.125	low reliability	-1173.50
BaAN-8	0.96	low reliability	-1.44	moderate reliability	96.53	34	low reliability	95.20	-3.179	low reliability	88.75
BaAN-9	1.04	low reliability	-1.44	moderate reliability	96.53	34	low reliability	95.20	0.164	low reliability	-24674.22
BaAN-10	0.75	low reliability	-3.00	low reliability	99.90	8	low reliability	98.87	-2.641	low reliability	61.18
BaAN-11	0.81	low reliability	-3.00	low reliability	99.90	8	low reliability	98.87	-2.502	low reliability	46.54
BaAN-12	1.69	experimental value	-0.84	experimental value	86.20	34	low reliability	95.20	-2.997	low reliability	82.90
BaAN-13	3.01	experimental value	0.70	experimental value	-378.63	229	experimental value	67.66	-1.992	experimental value	-72.98
BaAN-14	2.99	experimental value	0.65	experimental value	-326.58	229	experimental value	67.66	-1.678	experimental value	-256.45
BaAN-15	3.22	good reliability	1.17	moderate reliability	-1312.54	101	good reliability	85.73	-1.192	good reliability	-991.44
BaAN-16	0.96	good reliability	-0.44	moderate reliability	65.33	5	moderate reliability	99.29	-1.917	moderate reliability	-105.59
BaAN-17	1.02	low reliability	-0.68	moderate reliability	80.05	13	moderate reliability	98.16	-1.050	moderate reliability	-1413.56
BaAN-18	1.07	low reliability	-1.20	low reliability	93.97	5	low reliability	99.29	-1.308	low reliability	-735.60
BaAN-19	0.52	low reliability	-0.99	moderate reliability	90.23	13	low reliability	98.16	-0.636	low reliability	-3826.45

BaAN-20	0.85	moderate reliability	-1.33	moderate reliability	95.53	5	good reliability	99.29	-1.236	good reliability	-886.28
BaAN-21	0.59	low reliability	-1.64	moderate reliability	97.81	5	low reliability	99.29	-0.806	moderate reliability	-2554.61
BaAN-22	0.33	good reliability	-1.22	moderate reliability	94.25	5	good reliability	99.29	-2.539	experimental value	50.91
BaAN-23	0.53	good reliability	-1.61	moderate reliability	97.66	5	moderate reliability	99.29	-2.400	moderate reliability	32.39
BaAN-24	0.73	moderate reliability	-1.96	low reliability	98.95	23	experimental value	96.75	-2.460	experimental value	41.12
BaAN-25	0.28	low reliability	-1.27	moderate reliability	94.87	5	moderate reliability	99.29	-1.806	low reliability	-165.46
BaAN-26	2.30	moderate reliability	-0.98	low reliability	90.00	41	low reliability	94.21	-2.310	moderate reliability	16.82
BaAN-27	1.05	low reliability	-0.46	moderate reliability	66.89	34	low reliability	95.20	-1.819	low reliability	-157.63
BaAN-28	1.04	good reliability	-0.44	moderate reliability	65.33	5	moderate reliability	99.29	-2.555	moderate reliability	52.68
BaAN-29	0.44	moderate reliability	-0.06	moderate reliability	16.82	5	moderate reliability	99.29	-2.550	low reliability	52.14
BaAN-30	0.34	good reliability	-1.09	moderate reliability	92.24	7	experimental value	99.01	-1.731	experimental value	-215.50
BaP	2.69	experimental value	0.05	experimental value	0.00	708	experimental value	0.00	-2.373	experimental value	0.00
BaP-1	2.53	moderate reliability	-1.44	low reliability	96.76	41	low reliability	94.21	-1.829	moderate reliability	-249.95
BaP-2	1.87	low reliability	0.55	low reliability	-216.23	34	low reliability	95.20	-2.080	low reliability	-96.34
BaP-3	1.85	low reliability	1.00	low reliability	-791.25	34	low reliability	95.20	-1.219	low reliability	-1325.61
BaP-4	0.69	low reliability	-0.32	moderate reliability	57.34	34	low reliability	95.20	-0.318	low reliability	-11250.11
BaP-5	0.09	low reliability	-3.17	low reliability	99.94	16	low reliability	97.74	-1.765	low reliability	-305.51
BaP-6	1.44	moderate reliability	-0.58	moderate reliability	76.56	8	low reliability	98.87	0.346	low reliability	-52260.04
BaP-7	1.90	moderate reliability	-0.44	moderate reliability	67.64	26	good reliability	96.33	-1.174	low reliability	-1481.25
BaP-8	1.85	moderate reliability	-0.40	experimental value	64.52	23	experimental value	96.75	-1.811	experimental value	-264.75
BaP-9	1.52	low reliability	0.24	low reliability	-54.88	20	low reliability	97.18	-0.729	low reliability	-4305.55
BaP-10	1.94	good reliability	-0.45	moderate reliability	68.38	71	experimental value	89.97	-1.425	experimental value	-787.16
BaP-11	2.85	good reliability	-0.15	moderate reliability	36.90	48	good reliability	93.22	-0.731	good reliability	-4285.31
BaP-12	1.91	good reliability	-0.02	good reliability	14.89	71	experimental value	89.97	-1.379	experimental value	-886.28
BaP-13	2.26	good reliability	-0.21	good reliability	45.05	71	experimental value	89.97	-1.535	experimental value	-588.65
BaP-14	1.91	good reliability	-0.02	good reliability	14.89	71	experimental value	89.97	-1.379	experimental value	-886.28

BaP-15	3.24	good reliability	0.30	good reliability	-77.83	81	moderate reliability	88.56	-1.098	moderate reliability	-1783.65
BaP-16	0.40	low reliability	-2.42	low reliability	99.66	34	low reliability	95.20	-3.024	low reliability	77.66
BaP-17	1.44	low reliability	-1.32	moderate reliability	95.73	94	low reliability	86.72	-2.570	low reliability	36.47
BaP-18	1.47	low reliability	-1.70	low reliability	98.22	94	low reliability	86.72	-2.170	low reliability	-59.59
BaP-19	1.31	low reliability	-2.16	low reliability	99.38	8	moderate reliability	98.87	-2.678	moderate reliability	50.45
BaP-20	1.72	low reliability	-0.39	low reliability	63.69	41	low reliability	94.21	-0.028	low reliability	-22030.95
BaP-21	2.56	experimental value	0.32	experimental value	-86.21	708	experimental value	0.00	-2.234	experimental value	-37.72
BaP-22	1.84	low reliability	-1.68	low reliability	98.14	94	low reliability	86.72	-1.886	low reliability	-206.90
BaP-23	2.11	low reliability	-1.05	moderate reliability	92.06	23	low reliability	96.75	-0.627	low reliability	-5471.86
BaP-24	1.90	good reliability	-0.90	good reliability	88.78	41	low reliability	94.21	-2.280	moderate reliability	-23.88
BaP-25	2.17	moderate reliability	-1.44	low reliability	96.76	23	low reliability	96.75	-2.058	moderate reliability	-106.54
BaP-26	1.93	low reliability	-1.49	moderate reliability	97.12	41	low reliability	94.21	-2.327	low reliability	-11.17
BaP-27	1.86	low reliability	-0.04	moderate reliability	18.72	34	low reliability	95.20	-1.051	low reliability	-1998.94
BaP-28	1.92	low reliability	-1.82	low reliability	98.65	94	low reliability	86.72	-1.329	low reliability	-1006.62
BaP-29	2.53	moderate reliability	-1.44	low reliability	96.76	23	low reliability	96.75	-1.676	moderate reliability	-397.74
BaP-30	2.15	moderate reliability	-2.37	low reliability	99.62	23	low reliability	96.75	-1.692	moderate reliability	-379.73
BaP-31	1.92	low reliability	-1.82	low reliability	98.65	94	low reliability	86.72	-2.620	low reliability	43.38
BaP-32	1.87	low reliability	0.55	low reliability	-216.23	34	low reliability	95.20	-1.335	low reliability	-991.44
BaP-33	2.00	moderate reliability	-1.49	low reliability	97.12	67	moderate reliability	90.54	-1.332	moderate reliability	-999.01
BaP-34	3.01	experimental value	0.70	experimental value	-346.68	229	experimental value	67.66	-1.992	experimental value	-140.44
BaP-35	3.18	experimental value	0.63	experimental value	-280.19	229	good reliability	67.66	-1.909	good reliability	-191.07
BaP-36	2.91	good reliability	0.48	moderate reliability	-169.15	48	good reliability	93.22	-1.083	good reliability	-1849.84
BaP-37	1.43	moderate reliability	0.03	moderate reliability	4.50	41	moderate reliability	94.21	-1.668	moderate reliability	-406.99
BaP-38	2.71	good reliability	0.62	good reliability	-271.54	71	experimental value	89.97	-1.592	experimental value	-503.95
BaP-39	0.69	low reliability	-0.32	moderate reliability	57.34	34	low reliability	95.20	-0.318	low reliability	-11250.11
BaP-40	1.27	moderate reliability	-1.68	moderate reliability	98.14	23	experimental value	96.75	-2.014	experimental value	-128.56

BaP-41	0.25	low reliability	-0.98	moderate reliability	90.67	5	moderate reliability	99.29	-2.261	moderate reliability	-29.42
BaP-42	0.55	low reliability	0.28	low reliability	-69.82	7	low reliability	99.01	-1.602	low reliability	-490.20
BaP-43	0.18	low reliability	-1.12	moderate reliability	93.24	17	low reliability	97.60	-0.951	low reliability	-2542.41
BaP-44	1.10	low reliability	-0.04	moderate reliability	18.72	34	moderate reliability	95.20	-0.423	moderate reliability	-8812.51
BaP-45	0.21	moderate reliability	-3.46	low reliability	99.97	16	moderate reliability	97.74	-0.975	moderate reliability	-2400.35
BaP-46	1.44	low reliability	-0.51	moderate reliability	72.46	41	moderate reliability	94.21	-1.134	good reliability	-1633.80
BaP-47	1.56	moderate reliability	-0.50	moderate reliability	71.82	41	moderate reliability	94.21	-1.196	good reliability	-1403.14
BaP-48	1.07	moderate reliability	-0.31	moderate reliability	56.35	5	good reliability	99.29	-1.411	good reliability	-816.22
BaP-49	1.11	moderate reliability	-0.26	moderate reliability	51.02	5	good reliability	99.29	-1.386	good reliability	-870.51
BaP-50	1.04	low reliability	-1.29	moderate reliability	95.43	13	low reliability	98.16	-1.382	low reliability	-879.49
BaP-51	0.24	moderate reliability	-3.43	low reliability	99.97	7	low reliability	99.01	-1.980	low reliability	-147.17
BaP-52	0.73	moderate reliability	-1.96	low reliability	99.02	23	experimental value	96.75	-2.460	experimental value	18.15
BaP-53	0.23	low reliability	-1.88	low reliability	98.83	2	good reliability	99.72	-1.533	good reliability	-591.83
BaP-54	1.84	low reliability	-1.13	low reliability	93.39	41	low reliability	94.21	-0.802	low reliability	-3623.92
BaP-55	1.76	low reliability	-2.28	low reliability	99.53	8	low reliability	98.87	-1.052	low reliability	-1994.11
BaP-56	1.76	low reliability	-2.28	low reliability	99.53	8	low reliability	98.87	-1.052	low reliability	-1994.11
BaP-57	1.94	moderate reliability	-2.37	low reliability	99.62	8	low reliability	98.87	-1.967	moderate reliability	-154.68
BaP-58	1.91	low reliability	-2.28	low reliability	99.53	8	low reliability	98.87	-1.181	low reliability	-1455.97
BaP-59	2.08	moderate reliability	-2.37	low reliability	99.62	8	low reliability	98.87	-1.512	moderate reliability	-626.11
BaP-60	1.84	low reliability	-0.71	moderate reliability	82.62	23	low reliability	96.75	-3.392	low reliability	90.43
BaP-61	1.86	low reliability	-1.01	moderate reliability	91.29	23	moderate reliability	96.75	-3.406	low reliability	90.73
BaP-62	1.92	low reliability	-1.82	low reliability	98.65	94	low reliability	86.72	-2.620	low reliability	43.38
BaP-63	2.15	moderate reliability	-2.37	low reliability	99.62	8	low reliability	98.87	-2.426	moderate reliability	11.49
BaP-64	1.28	low reliability	-1.61	low reliability	97.81	94	low reliability	86.72	-2.519	low reliability	28.55
BaP-65	1.87	low reliability	-1.50	moderate reliability	97.18	34	moderate reliability	95.20	-2.154	moderate reliability	-65.58
BaP-66	2.09	low reliability	-1.14	moderate reliability	93.54	34	moderate reliability	95.20	-0.988	low reliability	-2326.61

BaP-67	1.92	low reliability	-1.82	low reliability	98.65	94	low reliability	86.72	-1.329	low reliability	-1006.62
BaP-68	2.15	moderate reliability	-2.37	low reliability	99.62	8	low reliability	98.87	-2.346	moderate reliability	-6.41
BaP-69	1.29	low reliability	-1.61	low reliability	97.81	94	low reliability	86.72	-2.698	low reliability	52.68
BaP-70	1.87	low reliability	-1.50	moderate reliability	97.18	34	moderate reliability	95.20	-2.333	moderate reliability	-9.65
BaP-71	2.16	moderate reliability	-1.18	moderate reliability	94.11	41	moderate reliability	94.21	-2.247	moderate reliability	-33.66
BaP-72	2.29	low reliability	-1.82	moderate reliability	98.65	41	low reliability	94.21	-1.645	low reliability	-434.56
BaP-73	1.91	low reliability	-2.28	low reliability	99.53	8	low reliability	98.87	-1.181	low reliability	-1455.97
BaP-74	1.92	low reliability	-1.82	low reliability	98.65	94	low reliability	86.72	-2.620	low reliability	43.38
BaP-75	1.92	low reliability	-1.82	low reliability	98.65	94	low reliability	86.72	-1.329	low reliability	-1006.62
BaP-76	1.75	low reliability	-2.28	low reliability	99.53	8	low reliability	98.87	-1.052	low reliability	-1994.11
BbF	2.98	good reliability	0.43	moderate reliability	0.00	708	good reliability	0.00	-2.453	good reliability	0.00
BbF-1	2.12	moderate reliability	-1.79	low reliability	99.40	8	low reliability	98.87	-2.140	moderate reliability	-105.59
BbF-2	2.17	moderate reliability	-1.79	low reliability	99.40	8	low reliability	98.87	-2.388	moderate reliability	-16.14
BbF-3	2.56	moderate reliability	-0.86	moderate reliability	94.87	41	low reliability	94.21	-2.074	moderate reliability	-139.33
BbF-4	2.56	moderate reliability	-0.86	moderate reliability	94.87	41	low reliability	94.21	-2.065	moderate reliability	-144.34
BbF-5	2.66	moderate reliability	-0.86	moderate reliability	94.87	23	low reliability	96.75	-2.212	moderate reliability	-74.18
BbF-6	2.56	moderate reliability	-0.86	moderate reliability	94.87	23	low reliability	96.75	-2.216	moderate reliability	-72.58
BbF-7	2.56	moderate reliability	-0.86	moderate reliability	94.87	23	low reliability	96.75	-1.940	moderate reliability	-225.84
BbF-8	2.63	moderate reliability	-0.86	moderate reliability	94.87	23	low reliability	96.75	-2.535	moderate reliability	17.21
BbF-9	2.29	moderate reliability	-0.86	moderate reliability	94.87	23	low reliability	96.75	-1.844	moderate reliability	-306.44
BbF-10	2.29	moderate reliability	-0.86	moderate reliability	94.87	41	low reliability	94.21	-2.073	moderate reliability	-139.88
BbF-11	2.56	low reliability	0.35	moderate reliability	16.82	22	low reliability	96.89	-0.101	low reliability	-22390.55
BbF-12	2.59	low reliability	0.37	moderate reliability	12.90	23	low reliability	96.75	-2.825	low reliability	57.54
BbF-13	1.81	moderate reliability	-2.55	low reliability	99.90	34	moderate reliability	95.20	-2.095	moderate reliability	-128.03
BbF-14	2.51	low reliability	0.47	low reliability	-9.65	94	low reliability	86.72	-0.073	low reliability	-23888.33
BbF-15	1.98	low reliability	0.52	moderate reliability	-23.03	8	low reliability	98.87	-0.267	low reliability	-15246.17

BbF-16	1.73	moderate reliability	0.52	moderate reliability	-23.03	94	moderate reliability	86.72	-0.381	moderate reliability	-11703.21
BbF-17	1.73	moderate reliability	0.52	moderate reliability	-23.03	94	moderate reliability	86.72	1.672	low reliability	-1333421.43
BghiP	2.79	good reliability	-0.53	moderate reliability	0.00	708	experimental value	0.00	-2.230	experimental value	0.00
BghiP-1	2.26	low reliability	0.71	low reliability	-1637.80	23	low reliability	96.75	-1.089	low reliability	-1283.57
BghiP-2	1.87	moderate reliability	-0.58	moderate reliability	10.87	5	low reliability	99.29	-0.901	low reliability	-2033.04
BghiP-3	1.80	moderate reliability	-0.48	moderate reliability	-12.20	34	low reliability	95.20	-1.666	moderate reliability	-266.44
BghiP-4	1.14	moderate reliability	1.55	low reliability	-11922.64	23	moderate reliability	96.75	-0.778	low reliability	-2731.39
BghiP-5	2.41	moderate reliability	-1.90	low reliability	95.73	23	low reliability	96.75	-2.128	moderate reliability	-26.47
BghiP-6	2.17	moderate reliability	-2.83	low reliability	99.50	5	low reliability	99.29	-2.144	moderate reliability	-21.90
BghiP-7	1.92	low reliability	-0.66	moderate reliability	25.87	23	low reliability	96.75	-1.229	low reliability	-902.31
BghiP-8	2.14	moderate reliability	-2.83	low reliability	99.50	5	low reliability	99.29	-2.084	moderate reliability	-39.96
BghiP-9	1.89	low reliability	-0.66	moderate reliability	25.87	23	low reliability	96.75	-2.321	low reliability	18.90
BghiP-10	2.33	low reliability	-1.59	low reliability	91.29	23	low reliability	96.75	-2.506	low reliability	47.03
BghiP-11	2.50	moderate reliability	-1.90	low reliability	95.73	23	low reliability	96.75	-2.068	moderate reliability	-45.21
BghiP-12	1.93	low reliability	-2.74	low reliability	99.38	8	low reliability	98.87	-2.946	low reliability	80.77
BghiP-13	1.87	low reliability	-2.14	low reliability	97.55	94	low reliability	86.72	-2.221	low reliability	-2.09
BghiP-14	1.92	low reliability	-2.85	low reliability	99.52	23	low reliability	96.75	-1.877	low reliability	-125.42
BghiP-15	1.33	low reliability	-3.35	low reliability	99.85	8	low reliability	98.87	-2.369	low reliability	27.39
BghiP-16	1.00	low reliability	-3.98	low reliability	99.96	8	low reliability	98.87	-2.495	low reliability	45.67
BghiP-17	0.94	low reliability	-3.26	low reliability	99.81	8	low reliability	98.87	-1.892	low reliability	-117.77
BkF	2.94	good reliability	-0.06	experimental value	0.00	708	experimental value	0.00	-2.230	experimental value	0.00
BkF-1	2.63	moderate reliability	-0.86	moderate reliability	84.15	23	low reliability	96.75	-2.535	moderate reliability	50.45
BkF-2	2.63	moderate reliability	-0.86	moderate reliability	84.15	23	low reliability	96.75	-2.302	moderate reliability	15.28
BkF-3	2.52	moderate reliability	-0.86	moderate reliability	84.15	23	low reliability	96.75	-2.287	moderate reliability	12.30
BkF-4	1.98	low reliability	-1.24	moderate reliability	93.39	94	low reliability	86.72	-2.934	low reliability	80.23
BkF-5	1.98	low reliability	-1.24	moderate reliability	93.39	94	low reliability	86.72	-2.934	low reliability	80.23

BkF-6	2.20	moderate reliability	-1.79	low reliability	98.14	8	low reliability	98.87	-2.681	moderate reliability	64.60
BkF-7	1.75	moderate reliability	-0.23	moderate reliability	32.39	23	low reliability	96.75	-2.855	low reliability	76.29
BkF-8	1.94	low reliability	-0.92	moderate reliability	86.20	34	moderate reliability	95.20	-2.036	moderate reliability	-56.31
BkF-9	1.75	moderate reliability	-1.98	low reliability	98.80	17	moderate reliability	97.60	-1.781	moderate reliability	-181.19
BkF-10	0.64	low reliability	-0.09	moderate reliability	6.67	34	low reliability	95.20	-2.127	low reliability	-26.77
BkF-11	0.61	low reliability	-0.49	moderate reliability	62.85	34	low reliability	95.20	-2.085	moderate reliability	-39.64
BkF-12	0.87	low reliability	-0.39	moderate reliability	53.23	34	low reliability	95.20	-2.132	moderate reliability	-25.31
BkF-13	0.88	moderate reliability	-0.57	moderate reliability	69.10	34	moderate reliability	95.20	-2.614	moderate reliability	58.70
BkF-14	1.16	moderate reliability	-0.27	moderate reliability	38.34	41	moderate reliability	94.21	-1.456	moderate reliability	-494.29
BkF-15	0.63	low reliability	-0.86	moderate reliability	84.15	7	low reliability	99.01	-1.467	low reliability	-479.43
BkF-16	0.57	low reliability	-1.32	moderate reliability	94.50	34	low reliability	95.20	-1.494	low reliability	-444.50
BkF-17	1.03	good reliability	-2.22	low reliability	99.31	41	moderate reliability	94.21	-1.649	moderate reliability	-281.07
BkF-18	0.78	good reliability	-0.44	moderate reliability	58.31	5	moderate reliability	99.29	-2.284	moderate reliability	11.69
CHR	2.24	experimental value	0.59	experimental value	0.00	708	experimental value	0.00	-2.230	experimental value	0.00
CHR-1	1.11	moderate reliability	-0.26	moderate reliability	85.87	5	good reliability	99.29	-1.386	good reliability	-598.23
CHR-2	1.07	moderate reliability	-0.31	moderate reliability	87.41	5	good reliability	99.29	-1.411	good reliability	-559.17
CHR-3	2.09	moderate reliability	-0.98	low reliability	97.31	41	low reliability	94.21	-2.405	moderate reliability	33.17
CHR-4	1.52	low reliability	-1.22	good reliability	98.45	34	low reliability	95.20	-2.558	low reliability	53.01
CHR-5	0.81	good reliability	-0.18	low reliability	83.02	5	good reliability	99.29	-1.882	moderate reliability	-122.84
CHR-6	0.34	good reliability	-1.09	moderate reliability	97.91	7	experimental value	99.01	-1.731	experimental value	-215.50
CHR-7	0.71	moderate reliability	-1.24	moderate reliability	98.52	7	good reliability	99.01	-1.554	good reliability	-374.24
CHR-8	0.44	low reliability	-0.88	moderate reliability	96.61	2	moderate reliability	99.72	-1.746	moderate reliability	-204.79
CHR-9	0.23	low reliability	-1.86	low reliability	99.65	7	experimental value	99.01	-1.476	experimental value	-467.54
CHR-10	2.28	low reliability	0.44	low reliability	29.21	41	low reliability	94.21	-0.635	low reliability	-3835.50
CHR-11	1.81	low reliability	-1.36	low reliability	98.88	34	low reliability	95.20	-1.766	low reliability	-191.07
DahA	2.80	experimental value	0.21	experimental value	0.00	708	good reliability	0.00	-2.230	experimental value	0.00

DahA-1	2.58	moderate reliability	-0.79	low reliability	90.00	23	low reliability	96.75	-1.604	moderate reliability	-322.67
DahA-2	3.23	low reliability	0.67	low reliability	-188.40	71	good reliability	89.97	-1.154	good reliability	-1091.24
DahA-3	2.63	moderate reliability	-0.98	low reliability	93.54	101	low reliability	85.73	-0.962	low reliability	-1753.53
DahA-4	1.95	moderate reliability	0.17	moderate reliability	8.80	67	low reliability	90.54	-0.718	low reliability	-3150.87
DahA-5	1.09	low reliability	-0.43	moderate reliability	77.09	34	low reliability	95.20	-1.622	low reliability	-305.51
DahA-6	0.49	low reliability	-0.46	moderate reliability	78.62	34	low reliability	95.20	-1.224	low reliability	-913.91
DahA-7	1.44	low reliability	-0.46	moderate reliability	78.62	5	low reliability	99.29	-1.604	low reliability	-322.67
FLR	2.78	experimental value	0.70	experimental value	0.00	229	experimental value	0.00	-1.740	experimental value	0.00
FLR-1	1.67	moderate reliability	-0.49	moderate reliability	93.54	41	low reliability	82.10	-1.727	moderate reliability	-3.04
FLR-2	1.42	moderate reliability	-0.49	moderate reliability	93.54	41	low reliability	82.10	-1.362	moderate reliability	-138.78
FLR-3	1.51	low reliability	-0.71	good reliability	96.11	5	low reliability	97.82	-1.977	low reliability	42.06
FLR-4	1.34	low reliability	-1.42	low reliability	99.24	8	low reliability	96.51	-2.183	low reliability	63.94
FLR-5	0.38	low reliability	-0.94	low reliability	97.71	34	low reliability	85.15	-0.662	low reliability	-1096.74
FLR-6	0.45	low reliability	-2.08	low reliability	99.83	41	low reliability	82.10	-1.231	moderate reliability	-222.85
FLR-7	0.37	low reliability	-2.08	low reliability	99.83	7	moderate reliability	96.94	-1.859	moderate reliability	23.97
FLR-8	0.82	low reliability	-1.71	moderate reliability	99.61	13	moderate reliability	94.32	-1.838	moderate reliability	20.20
FLR-9	0.73	low reliability	-2.22	low reliability	99.88	13	moderate reliability	94.32	-1.850	low reliability	22.38
FLR-10	1.46	moderate reliability	-0.10	moderate reliability	84.15	41	low reliability	82.10	-1.241	moderate reliability	-215.50
FLR-11	0.69	low reliability	-1.97	moderate reliability	99.79	8	low reliability	96.51	-1.289	low reliability	-182.49
FLR-12	1.11	low reliability	-1.04	moderate reliability	98.18	41	low reliability	82.10	-1.313	low reliability	-167.30
FLR-13	0.83	good reliability	-0.98	moderate reliability	97.91	8	moderate reliability	96.51	-1.721	moderate reliability	-4.47
FLR-14	1.11	low reliability	-1.89	moderate reliability	99.74	34	moderate reliability	85.15	-1.276	moderate reliability	-191.07
FLR-15	0.33	good reliability	-1.22	moderate reliability	98.80	5	good reliability	97.82	-2.539	experimental value	84.11
FLR-16	0.08	moderate reliability	-2.30	low reliability	99.90	7	good reliability	96.94	-1.764	moderate reliability	5.38
FLR-17	1.24	low reliability	-1.42	low reliability	99.24	8	low reliability	96.51	-1.448	low reliability	-95.88
FLR-18	0.43	low reliability	-0.81	moderate reliability	96.91	34	low reliability	85.15	-0.712	low reliability	-966.60

FLR-19	0.43	low reliability	-1.58	moderate reliability	99.48	13	low reliability	94.32	-1.151	moderate reliability	-288.15
FLR-20	0.38	low reliability	-1.47	moderate reliability	99.32	7	low reliability	96.94	-1.779	low reliability	8.59
FLR-21	0.82	low reliability	-1.34	moderate reliability	99.09	13	moderate reliability	94.32	-1.101	moderate reliability	-335.51
FLR-22	0.49	low reliability	-2.30	low reliability	99.90	5	low reliability	97.82	-1.016	moderate reliability	-429.66
FLR-23	0.79	low reliability	-1.60	moderate reliability	99.50	13	moderate reliability	94.32	-0.995	moderate reliability	-455.90
FLR-24	0.42	moderate reliability	-1.06	moderate reliability	98.26	5	good reliability	97.82	-1.722	good reliability	-4.23
FLR-25	0.34	good reliability	-1.09	moderate reliability	98.38	7	experimental value	96.94	-1.731	experimental value	-2.09
FLR-26	0.11	moderate reliability	-2.03	low reliability	99.81	5	good reliability	97.82	-1.735	moderate reliability	-1.16
FLR-27	0.23	low reliability	-1.86	low reliability	99.72	7	experimental value	96.94	-1.476	experimental value	-83.65
FLR-28	0.52	moderate reliability	-1.50	low reliability	99.37	2	good reliability	99.13	-1.392	experimental value	-122.84
FRT	2.71	experimental value	-0.02	experimental value	0.00	708	experimental value	0.00	-2.230	experimental value	0.00
FRT-1	2.04	good reliability	-1.05	moderate reliability	90.67	41	low reliability	94.21	-1.445	moderate reliability	-509.54
FRT-2	2.04	good reliability	-1.05	moderate reliability	90.67	41	low reliability	94.21	-1.774	moderate reliability	-185.76
FRT-3	2.04	good reliability	-1.05	moderate reliability	90.67	41	low reliability	94.21	-1.669	moderate reliability	-263.92
FRT-4	1.65	moderate reliability	-1.98	low reliability	98.90	8	low reliability	98.87	-1.935	moderate reliability	-97.24
FRT-5	1.41	low reliability	0.02	moderate reliability	-9.65	8	low reliability	98.87	-0.688	low reliability	-3383.37
FRT-6	0.59	low reliability	-1.80	low reliability	98.34	94	low reliability	86.72	-0.755	low reliability	-2885.38
FRT-7	0.61	low reliability	-1.80	low reliability	98.34	94	low reliability	86.72	-1.084	low reliability	-1299.59
FRT-8	0.49	low reliability	0.07	low reliability	-23.03	94	low reliability	86.72	0.258	low reliability	-30660.97
FRT-9	0.28	low reliability	-0.59	low reliability	73.08	94	low reliability	86.72	0.820	low reliability	-112101.85
FRT-10	0.22	low reliability	-1.31	low reliability	94.87	94	low reliability	86.72	-0.525	low reliability	-4969.91
FRT-11	-1.03	low reliability	-2.48	low reliability	99.65	94	low reliability	86.72	1.203	low reliability	-270919.16
FRT-12	1.09	moderate reliability	0.33	moderate reliability	-123.87	34	moderate reliability	95.20	-0.025	low reliability	-15932.45
FRT-13	1.75	moderate reliability	-1.98	low reliability	98.90	8	low reliability	98.87	-2.566	moderate reliability	53.87
FRT-14	2.16	moderate reliability	-0.41	moderate reliability	59.26	94	moderate reliability	86.72	-2.448	moderate reliability	39.47
FRT-15	0.68	low reliability	-0.04	low reliability	4.50	34	low reliability	95.20	-0.422	low reliability	-6326.88

FRT-16	0.64	low reliability	-1.28	moderate reliability	94.50	34	low reliability	95.20	-2.585	low reliability	55.84
FRT-17	0.72	low reliability	-1.56	moderate reliability	97.12	41	low reliability	94.21	-1.560	low reliability	-367.74
FRT-18	1.06	low reliability	-0.91	moderate reliability	87.12	41	moderate reliability	94.21	-1.615	moderate reliability	-312.10
FRT-19	0.80	moderate reliability	-2.27	low reliability	99.44	34	moderate reliability	95.20	-1.279	moderate reliability	-793.31
FRT-20	0.77	low reliability	-0.84	moderate reliability	84.86	5	low reliability	99.29	-1.818	low reliability	-158.23
FRT-21	1.33	low reliability	0.33	moderate reliability	-123.87	8	low reliability	98.87	-2.374	low reliability	28.22
FRT-22	1.57	moderate reliability	-1.98	low reliability	98.90	8	low reliability	98.87	-1.913	moderate reliability	-107.49
FRT-23	2.01	moderate reliability	-0.41	moderate reliability	59.26	94	moderate reliability	86.72	-1.591	moderate reliability	-335.51
FRT-24	0.73	low reliability	-0.02	low reliability	0.00	34	low reliability	95.20	-2.115	low reliability	-30.32
FRT-25	0.57	low reliability	-0.04	low reliability	4.50	34	low reliability	95.20	-0.013	low reliability	-16381.62
FRT-26	1.30	low reliability	-0.59	moderate reliability	73.08	34	low reliability	95.20	-1.971	low reliability	-81.55
FRT-27	1.34	low reliability	-1.19	good reliability	93.24	5	low reliability	99.29	-1.242	low reliability	-872.75
FRT-28	1.46	moderate reliability	-0.10	moderate reliability	16.82	41	low reliability	94.21	-1.241	moderate reliability	-874.99
FRT-29	1.51	low reliability	-0.71	good reliability	79.58	5	low reliability	99.29	-1.977	low reliability	-79.06
FRT-30	0.74	low reliability	-1.56	moderate reliability	97.12	8	low reliability	98.87	-2.331	moderate reliability	20.75
FRT-31	0.14	moderate reliability	-1.60	moderate reliability	97.37	5	moderate reliability	99.29	-3.473	low reliability	94.29
FRT-32	1.13	low reliability	0.28	moderate reliability	-99.53	8	moderate reliability	98.87	-1.061	moderate reliability	-1375.71
FRT-33	1.75	moderate reliability	-1.98	low reliability	98.90	17	moderate reliability	97.60	-1.781	moderate reliability	-181.19
FRT-34	2.10	moderate reliability	-0.41	moderate reliability	59.26	94	moderate reliability	86.72	-1.641	moderate reliability	-288.15
FRT-35	0.78	good reliability	-0.44	moderate reliability	61.98	5	moderate reliability	99.29	-2.284	moderate reliability	11.69
FRT-36	1.37	low reliability	0.31	moderate reliability	-113.80	34	low reliability	95.20	0.431	low reliability	-45714.19
FRT-37	1.65	moderate reliability	-1.98	low reliability	98.90	8	low reliability	98.87	-1.935	moderate reliability	-97.24
FRT-38	2.07	moderate reliability	-0.41	moderate reliability	59.26	94	moderate reliability	86.72	-1.613	moderate reliability	-314.00
FRT-39	1.46	low reliability	0.54	low reliability	-263.08	34	low reliability	95.20	-1.971	low reliability	-81.55
FRT-40	0.20	low reliability	-1.43	low reliability	96.11	8	low reliability	98.87	-0.455	low reliability	-5856.62
FRT-41	0.95	moderate reliability	-2.36	low reliability	99.54	8	moderate reliability	98.87	-2.276	moderate reliability	10.05

FRT-42	0.83	good reliability	-0.98	moderate reliability	89.04	8	moderate reliability	98.87	-1.721	moderate reliability	-222.85
FRT-43	1.11	low reliability	-1.89	moderate reliability	98.65	34	moderate reliability	95.20	-1.276	moderate reliability	-799.50
FRT-44	0.33	good reliability	-1.22	moderate reliability	93.69	5	good reliability	99.29	-2.539	experimental value	50.91
FRT-45	0.08	moderate reliability	-2.30	low reliability	99.48	7	good reliability	99.01	-1.764	moderate reliability	-192.42
FRT-46	3.01	experimental value	0.70	experimental value	-424.81	229	experimental value	67.66	-1.992	experimental value	-72.98
FRT-47	3.08	good reliability	0.49	moderate reliability	-223.59	708	good reliability	0.00	-1.130	moderate reliability	-1158.93
FRT-48	2.99	experimental value	0.65	experimental value	-367.74	229	experimental value	67.66	-1.678	experimental value	-256.45
FRT-49	1.91	moderate reliability	-0.56	moderate reliability	71.16	5	moderate reliability	99.29	-1.686	moderate reliability	-249.95
FRT-50	2.24	moderate reliability	0.23	moderate reliability	-77.83	41	good reliability	94.21	-1.779	moderate reliability	-182.49
FRT-51	1.81	low reliability	0.00	low reliability	-4.71	41	low reliability	94.21	-1.507	low reliability	-428.45
FRT-52	1.13	low reliability	-0.41	moderate reliability	59.26	41	low reliability	94.21	-1.667	low reliability	-265.59
FRT-53	0.33	good reliability	-1.22	moderate reliability	93.69	5	good reliability	99.29	-2.539	experimental value	50.91
FRT-54	1.11	good reliability	-0.05	moderate reliability	6.67	23	good reliability	96.75	-1.449	low reliability	-503.95
IcdP	2.74	good reliability	0.07	moderate reliability	0.00	708	good reliability	0.00	-2.536	good reliability	0.00
IcdP-1	2.42	moderate reliability	-1.32	moderate reliability	95.93	23	low reliability	96.75	-1.626	moderate reliability	-712.83
IcdP-2	2.42	moderate reliability	-1.32	moderate reliability	95.93	23	low reliability	96.75	-2.047	moderate reliability	-208.32
IcdP-3	2.54	low reliability	-1.32	moderate reliability	95.93	23	low reliability	96.75	-1.896	moderate reliability	-336.52
IcdP-4	2.54	low reliability	-1.32	moderate reliability	95.93	23	low reliability	96.75	-2.295	moderate reliability	-74.18
IcdP-5	1.98	low reliability	-2.16	low reliability	99.41	8	low reliability	98.87	-2.979	low reliability	63.94
IcdP-6	1.62	low reliability	-3.09	low reliability	99.93	8	low reliability	98.87	-1.703	low reliability	-580.77
IcdP-7	1.62	low reliability	-3.09	low reliability	99.93	8	low reliability	98.87	-1.615	low reliability	-733.68
IcdP-8	1.92	low reliability	-1.56	moderate reliability	97.66	94	low reliability	86.72	-2.268	low reliability	-85.35
IcdP-9	2.55	low reliability	-1.32	moderate reliability	95.93	23	low reliability	96.75	-2.225	moderate reliability	-104.64
IcdP-10	0.52	moderate reliability	-1.50	low reliability	97.31	2	good reliability	99.72	-1.392	experimental value	-1293.16
IcdP-11	2.39	experimental value	-0.37	experimental value	63.69	5	low reliability	99.29	-0.926	low reliability	-3973.80
IcdP-12	0.59	low reliability	-0.72	moderate reliability	83.78	34	low reliability	95.20	-0.140	low reliability	-24788.57

IcdP-13	3.23	low reliability	0.67	low reliability	-298.11	71	good reliability	89.97	-1.154	good reliability	-2309.91
IcdP-14	1.43	low reliability	-0.58	moderate reliability	77.61	5	low reliability	99.29	-0.901	low reliability	-4215.19
IcdP-15	1.87	low reliability	-1.94	low reliability	99.02	101	low reliability	85.73	-1.035	low reliability	-3069.57
IcdP-16	0.80	low reliability	-0.19	moderate reliability	45.05	34	moderate reliability	95.20	-0.623	moderate reliability	-8084.65
IcdP-17	1.90	moderate reliability	-0.56	moderate reliability	76.56	41	good reliability	94.21	-1.269	moderate reliability	-1749.27
IcdP-18	1.67	low reliability	-2.98	low reliability	99.91	217	low reliability	69.35	-2.337	low reliability	-58.12
IcdP-19	1.83	moderate reliability	-0.59	moderate reliability	78.12	94	low reliability	86.72	-0.596	low reliability	-8609.64
IcdP-20	2.17	moderate reliability	-2.25	low reliability	99.52	8	low reliability	98.87	-2.368	moderate reliability	-47.23
IcdP-21	1.72	low reliability	-1.36	moderate reliability	96.28	23	moderate reliability	96.75	-3.166	low reliability	76.56
IcdP-22	1.73	low reliability	-2.92	low reliability	99.90	8	low reliability	98.87	-1.649	low reliability	-670.90
IcdP-23	1.73	low reliability	-1.64	low reliability	98.05	8	low reliability	98.87	-1.848	low reliability	-387.53
IcdP-24	1.77	low reliability	-1.65	moderate reliability	98.09	23	moderate reliability	96.75	-2.947	low reliability	61.18
IcdP-25	1.98	low reliability	-2.16	low reliability	99.41	8	low reliability	98.87	-2.979	low reliability	63.94
IcdP-26	2.17	moderate reliability	-2.25	low reliability	99.52	8	low reliability	98.87	-1.894	moderate reliability	-338.53
IcdP-27	1.85	moderate reliability	-0.89	moderate reliability	89.04	23	moderate reliability	96.75	-3.137	low reliability	74.94
IcdP-28	1.95	low reliability	-1.24	moderate reliability	95.10	94	low reliability	86.72	-1.196	low reliability	-2087.76
IcdP-29	2.17	moderate reliability	-1.79	low reliability	98.62	8	low reliability	98.87	-2.089	moderate reliability	-179.90
IcdP-30	1.84	low reliability	0.65	low reliability	-280.19	94	low reliability	86.72	-0.275	low reliability	-18138.96
IcdP-31	2.50	experimental value	0.41	experimental value	-118.78	71	experimental value	89.97	-1.652	experimental value	-665.60
IcdP-32	1.94	good reliability	0.71	low reliability	-336.52	41	moderate reliability	94.21	-1.243	moderate reliability	-1863.36
IcdP-33	0.50	low reliability	-2.28	low reliability	99.55	23	low reliability	96.75	-1.693	low reliability	-596.63
IcdP-34	0.45	low reliability	-0.42	moderate reliability	67.64	34	moderate reliability	95.20	-1.782	moderate reliability	-467.54
IcdP-35	1.03	low reliability	-0.68	moderate reliability	82.22	5	low reliability	99.29	-1.423	low reliability	-1197.18
IcdP-36	2.21	low reliability	-1.32	moderate reliability	95.93	23	low reliability	96.75	-2.117	moderate reliability	-162.42
IcdP-37	2.55	low reliability	-1.32	moderate reliability	95.93	23	low reliability	96.75	-2.225	moderate reliability	-104.64
NAP	2.50	experimental value	0.41	experimental value	0.00	71	experimental value	0.00	-1.230	experimental value	0.00

NAP-1	0.81	low reliability	-0.07	low reliability	66.89	41	low reliability	42.25	-1.398	low reliability	32.08
NAP-2	0.47	low reliability	-1.22	moderate reliability	97.66	16	low reliability	77.46	-1.807	low reliability	73.51
NAP-3	0.71	moderate reliability	-1.24	moderate reliability	97.76	7	good reliability	90.14	-1.554	good reliability	52.58
NAP-4	1.11	moderate reliability	-0.26	moderate reliability	78.62	5	good reliability	92.96	-1.386	good reliability	30.18
NAP-5	0.37	low reliability	-0.61	moderate reliability	90.45	17	low reliability	76.06	-1.004	low reliability	-68.27
NAP-6	0.33	low reliability	-1.06	moderate reliability	96.61	34	low reliability	52.11	-1.904	low reliability	78.82
NAP-7	0.96	low reliability	-0.87	moderate reliability	94.75	23	low reliability	67.61	-2.234	low reliability	90.09
NAP-8	1.07	moderate reliability	-0.31	moderate reliability	80.95	5	good reliability	92.96	-1.411	good reliability	34.08
NAP-9	0.85	moderate reliability	-1.33	moderate reliability	98.18	5	good reliability	92.96	-1.236	good reliability	1.37
NAP-10	0.52	low reliability	-0.99	moderate reliability	96.02	13	low reliability	81.69	-0.636	low reliability	-292.64
NAP-11	0.69	moderate reliability	-1.43	low reliability	98.55	5	good reliability	92.96	-1.415	good reliability	34.69
NAP-12	0.79	moderate reliability	-1.42	low reliability	98.52	5	good reliability	92.96	-1.261	good reliability	6.89
NAP-13	0.50	low reliability	-1.34	low reliability	98.22	34	low reliability	52.11	-2.413	low reliability	93.44
NAP-14	-0.21	low reliability	-4.49	low reliability	100.00	26	low reliability	63.38	-3.185	low reliability	98.89
NAP-15	1.18	moderate reliability	0.21	low reliability	36.90	7	good reliability	90.14	-1.666	good reliability	63.36
NAP-16	-0.93	low reliability	-7.48	low reliability	100.00	8	low reliability	88.73	-4.616	low reliability	99.96
NAP-17	-0.91	low reliability	-7.90	low reliability	100.00	8	low reliability	88.73	-3.897	low reliability	99.78
NAP-18	-0.90	low reliability	-8.31	low reliability	100.00	8	low reliability	88.73	-4.077	low reliability	99.86
NAP-19	-0.92	low reliability	-6.90	low reliability	100.00	8	low reliability	88.73	-3.164	low reliability	98.84
NAP-20	-0.92	low reliability	-6.99	low reliability	100.00	8	low reliability	88.73	-3.272	low reliability	99.09
NAP-21	-0.92	low reliability	-7.20	low reliability	100.00	8	low reliability	88.73	-3.208	low reliability	98.95
NAP-22	-0.92	low reliability	-6.90	low reliability	100.00	8	low reliability	88.73	-2.538	low reliability	95.08
NAP-23	0.47	low reliability	-1.22	moderate reliability	97.66	16	low reliability	77.46	-1.807	low reliability	73.51
NAP-24	0.40	low reliability	-0.89	low reliability	94.99	34	low reliability	52.11	-2.065	low reliability	85.38
NAP-25	0.25	low reliability	-0.72	moderate reliability	92.59	34	low reliability	52.11	-1.444	low reliability	38.91
NAP-26	0.75	low reliability	-1.22	moderate reliability	97.66	13	low reliability	81.69	-0.788	low reliability	-176.69

NAP-27	0.20	low reliability	-0.86	moderate reliability	94.63	16	low reliability	77.46	-2.096	low reliability	86.39
NAP-28	0.44	low reliability	-0.88	moderate reliability	94.87	2	moderate reliability	97.18	-1.746	moderate reliability	69.52
NAP-29	0.34	good reliability	-1.09	moderate reliability	96.84	7	experimental value	90.14	-1.731	experimental value	68.45
NAP-30	0.11	moderate reliability	-2.03	low reliability	99.64	5	good reliability	92.96	-1.735	moderate reliability	68.74
NAP-31	0.23	low reliability	-1.86	low reliability	99.46	7	experimental value	90.14	-1.476	experimental value	43.25
NAP-32	0.59	low reliability	-1.64	moderate reliability	99.11	5	low reliability	92.96	-0.806	moderate reliability	-165.46
PHE	3.01	experimental value	0.70	experimental value	0.00	229	experimental value	0.00	-1.740	experimental value	0.00
PHE-1	1.90	moderate reliability	-0.56	moderate reliability	94.50	41	good reliability	82.10	-1.866	moderate reliability	25.18
PHE-2	1.65	moderate reliability	-0.56	moderate reliability	94.50	41	good reliability	82.10	-1.237	moderate reliability	-218.42
PHE-3	1.90	moderate reliability	-0.56	moderate reliability	94.50	41	good reliability	82.10	-1.269	moderate reliability	-195.80
PHE-4	1.65	moderate reliability	-0.56	moderate reliability	94.50	41	good reliability	82.10	-1.256	moderate reliability	-204.79
PHE-5	1.65	moderate reliability	-0.56	moderate reliability	94.50	41	good reliability	82.10	-1.187	moderate reliability	-257.27
PHE-6	1.01	low reliability	1.00	low reliability	-99.53	41	low reliability	82.10	-1.726	low reliability	-3.28
PHE-7	1.11	low reliability	-1.04	moderate reliability	98.18	41	low reliability	82.10	-1.313	low reliability	-167.30
PHE-8	1.46	moderate reliability	-0.10	moderate reliability	84.15	41	low reliability	82.10	-1.241	moderate reliability	-215.50
PHE-9	0.94	low reliability	-0.68	moderate reliability	95.83	34	low reliability	85.15	-1.598	low reliability	-38.68
PHE-10	1.56	moderate reliability	-1.48	low reliability	99.34	8	moderate reliability	96.51	-2.033	moderate reliability	49.07
PHE-11	1.56	moderate reliability	-1.48	low reliability	99.34	8	moderate reliability	96.51	-2.033	moderate reliability	49.07
PHE-12	0.73	low reliability	-0.44	moderate reliability	92.76	34	low reliability	85.15	-1.293	low reliability	-179.90
PHE-13	0.62	low reliability	-0.29	moderate reliability	89.77	34	low reliability	85.15	-1.757	low reliability	3.84
PHE-14	0.63	low reliability	-0.25	moderate reliability	88.78	7	moderate reliability	96.94	-1.407	moderate reliability	-115.28
PHE-15	0.74	good reliability	-0.18	low reliability	86.82	5	good reliability	97.82	-1.200	moderate reliability	-246.74
PHE-16	1.50	moderate reliability	-0.93	moderate reliability	97.66	41	low reliability	82.10	-0.550	low reliability	-1448.82
PHE-17	1.03	low reliability	-1.49	moderate reliability	99.35	8	low reliability	96.51	-2.312	low reliability	73.21
PHE-18	1.03	low reliability	-1.49	moderate reliability	99.35	8	low reliability	96.51	-2.312	low reliability	73.21
PHE-19	1.51	moderate reliability	-0.05	low reliability	82.22	20	moderate reliability	91.27	-2.161	moderate reliability	62.07

PHE-20	1.35	moderate reliability	-1.48	low reliability	99.34	8	moderate reliability	96.51	-1.471	moderate reliability	-85.78
PHE-21	1.35	moderate reliability	-1.48	low reliability	99.34	8	moderate reliability	96.51	-1.471	moderate reliability	-85.78
PHE-22	0.78	low reliability	-0.44	moderate reliability	92.76	34	low reliability	85.15	-1.391	low reliability	-123.36
PHE-23	0.66	low reliability	-0.29	moderate reliability	89.77	17	low reliability	92.58	-2.247	low reliability	68.88
PHE-24	0.70	low reliability	-0.25	moderate reliability	88.78	5	moderate reliability	97.82	-1.898	moderate reliability	30.50
PHE-25	0.81	good reliability	-0.18	low reliability	86.82	5	good reliability	97.82	-1.882	moderate reliability	27.89
PHE-26	1.56	low reliability	-0.93	moderate reliability	97.66	41	low reliability	82.10	-1.043	low reliability	-397.74
PHE-27	1.03	good reliability	0.51	low reliability	35.43	34	low reliability	85.15	-1.945	low reliability	37.63
PHE-28	0.97	good reliability	0.51	low reliability	35.43	20	low reliability	91.27	-2.119	low reliability	58.22
PHE-29	0.96	good reliability	-0.44	moderate reliability	92.76	5	moderate reliability	97.82	-1.917	moderate reliability	33.47
PHE-30	0.71	moderate reliability	-1.24	moderate reliability	98.85	7	good reliability	96.94	-1.554	good reliability	-53.46
PHE-31	0.20	low reliability	-0.86	moderate reliability	97.25	16	low reliability	93.01	-2.096	low reliability	55.94
PHE-32	0.44	low reliability	-0.88	moderate reliability	97.37	2	moderate reliability	99.13	-1.746	moderate reliability	1.37
PHE-33	0.34	good reliability	-1.09	moderate reliability	98.38	7	experimental value	96.94	-1.731	experimental value	-2.09
PHE-34	0.11	moderate reliability	-2.03	low reliability	99.81	5	good reliability	97.82	-1.735	moderate reliability	-1.16
PHE-35	0.75	low reliability	-1.22	moderate reliability	98.80	13	low reliability	94.32	-0.788	low reliability	-795.36
PHE-36	0.44	moderate reliability	-0.06	moderate reliability	82.62	5	moderate reliability	97.82	-2.550	low reliability	84.51
PHE-37	0.25	low reliability	-0.61	moderate reliability	95.10	5	moderate reliability	97.82	-2.555	moderate reliability	84.69
PHE-38	0.33	good reliability	-1.22	moderate reliability	98.80	5	good reliability	97.82	-2.539	experimental value	84.11
PHE-39	-0.01	moderate reliability	-2.87	low reliability	99.97	7	moderate reliability	96.94	-2.520	moderate reliability	83.40
PHE-40	0.08	moderate reliability	-2.30	low reliability	99.90	7	good reliability	96.94	-1.764	moderate reliability	5.38
PHE-41	0.09	low reliability	-0.84	low reliability	97.12	16	low reliability	93.01	-2.451	low reliability	80.55
PHE-42	1.35	moderate reliability	-1.48	low reliability	99.34	8	moderate reliability	96.51	-1.224	moderate reliability	-228.10
PHE-43	1.51	moderate reliability	-0.25	low reliability	88.78	20	moderate reliability	91.27	-1.187	moderate reliability	-257.27
PHE-44	1.53	low reliability	0.44	moderate reliability	45.05	20	moderate reliability	91.27	-1.657	moderate reliability	-21.06
PHE-45	1.03	low reliability	0.25	moderate reliability	64.52	34	low reliability	85.15	-1.961	low reliability	39.88

PHE-46	0.87	moderate reliability	-0.24	moderate reliability	88.52	34	low reliability	85.15	-2.543	low reliability	84.26
PHE-47	0.68	low reliability	-0.65	moderate reliability	95.53	8	moderate reliability	96.51	-2.228	moderate reliability	67.49
PHE-48	0.74	low reliability	-0.59	moderate reliability	94.87	5	low reliability	97.82	-1.774	low reliability	7.53
PHE-49	1.03	moderate reliability	0.00	moderate reliability	80.05	20	moderate reliability	91.27	-0.730	low reliability	-923.29
PHE-50	0.25	low reliability	-0.61	moderate reliability	95.10	5	moderate reliability	97.82	-2.555	moderate reliability	84.69
PHE-51	0.33	good reliability	-1.22	moderate reliability	98.80	5	good reliability	97.82	-2.539	experimental value	84.11
PHE-52	1.60	low reliability	-0.21	low reliability	87.70	41	low reliability	82.10	-2.603	low reliability	86.29
PHE-53	1.03	low reliability	-1.49	moderate reliability	99.35	8	low reliability	96.51	-2.312	low reliability	73.21
PHE-54	1.36	low reliability	0.25	low reliability	64.52	41	low reliability	82.10	-0.308	low reliability	-2603.96
PHE-55	1.01	low reliability	-0.93	moderate reliability	97.66	17	low reliability	92.58	-0.962	low reliability	-499.79
PHE-56	1.78	moderate reliability	-0.70	moderate reliability	96.02	41	good reliability	82.10	-1.333	moderate reliability	-155.27
PYR	2.56	experimental value	0.32	experimental value	0.00	708	experimental value	0.00	-2.230	experimental value	0.00
PYR-1	2.00	moderate reliability	-1.63	low reliability	98.88	41	moderate reliability	94.21	-1.758	moderate reliability	-196.48
PYR-2	1.70	moderate reliability	-2.56	low reliability	99.87	5	moderate reliability	99.29	-1.760	moderate reliability	-195.12
PYR-3	1.70	moderate reliability	-2.56	low reliability	99.87	5	moderate reliability	99.29	-1.760	moderate reliability	-195.12
PYR-4	1.65	moderate reliability	-0.56	moderate reliability	86.82	41	good reliability	94.21	-1.187	moderate reliability	-1004.08
PYR-5	1.70	low reliability	-0.11	moderate reliability	62.85	41	moderate reliability	94.21	-1.481	moderate reliability	-461.05
PYR-6	1.46	low reliability	-0.67	low reliability	89.77	41	low reliability	94.21	0.072	low reliability	-19944.72
PYR-7	1.25	low reliability	-2.47	low reliability	99.84	8	low reliability	98.87	-0.825	low reliability	-2440.97
PYR-8	1.34	low reliability	0.26	moderate reliability	12.90	34	low reliability	95.20	-1.145	low reliability	-1116.19
PYR-9	1.33	low reliability	0.26	moderate reliability	12.90	34	low reliability	95.20	-0.972	low reliability	-1711.34
PYR-10	1.70	moderate reliability	-2.56	low reliability	99.87	8	moderate reliability	98.87	-2.078	moderate reliability	-41.91
PYR-11	1.70	moderate reliability	-2.56	low reliability	99.87	8	moderate reliability	98.87	-2.078	moderate reliability	-41.91
PYR-12	0.66	low reliability	0.08	low reliability	42.46	34	low reliability	95.20	-2.326	low reliability	19.83
PYR-13	0.88	low reliability	-1.68	moderate reliability	99.00	34	low reliability	95.20	-2.329	low reliability	20.38
PYR-14	0.87	low reliability	-1.15	moderate reliability	96.61	41	low reliability	94.21	-1.367	low reliability	-629.46

PYR-15	2.12	moderate reliability	-0.99	low reliability	95.10	94	moderate reliability	86.72	-1.938	moderate reliability	-95.88
PYR-16	1.37	low reliability	-1.55	low reliability	98.65	20	low reliability	97.18	-1.584	low reliability	-342.59
PYR-17	1.25	low reliability	-2.47	low reliability	99.84	8	low reliability	98.87	-0.825	low reliability	-2440.97
PYR-18	1.53	moderate reliability	-2.56	low reliability	99.87	8	moderate reliability	98.87	-1.896	moderate reliability	-115.77
PYR-19	1.63	moderate reliability	-0.58	moderate reliability	87.41	34	moderate reliability	95.20	-2.415	moderate reliability	34.69
PYR-20	1.42	moderate reliability	-1.03	moderate reliability	95.53	5	moderate reliability	99.29	-1.622	moderate reliability	-305.51
PYR-21	0.49	low reliability	-2.90	low reliability	99.94	7	low reliability	99.01	-1.556	low reliability	-372.06
PYR-22	0.67	good reliability	-2.99	low reliability	99.95	7	low reliability	99.01	-1.584	moderate reliability	-342.59
PYR-23	0.87	moderate reliability	-2.19	low reliability	99.69	5	low reliability	99.29	-3.170	low reliability	88.52
PYR-24	0.73	good reliability	-1.93	low reliability	99.44	5	low reliability	99.29	-2.377	moderate reliability	28.71
PYR-25	0.33	good reliability	-1.22	moderate reliability	97.12	5	good reliability	99.29	-2.539	experimental value	50.91
PYR-26	0.59	low reliability	-0.83	moderate reliability	92.92	8	low reliability	98.87	-1.257	low reliability	-839.72
PYR-27	1.35	moderate reliability	-1.48	low reliability	98.42	8	moderate reliability	98.87	-1.471	moderate reliability	-474.12
PYR-28	1.91	moderate reliability	-0.63	good reliability	88.78	34	moderate reliability	95.20	-1.541	moderate reliability	-388.65
PYR-29	1.65	moderate reliability	-0.56	moderate reliability	86.82	41	good reliability	94.21	-1.187	moderate reliability	-1004.08
PYR-30	1.56	low reliability	-0.93	moderate reliability	94.38	41	low reliability	94.21	-1.043	low reliability	-1438.15
PYR-31	0.68	low reliability	0.35	low reliability	-7.15	34	low reliability	95.20	-0.064	low reliability	-14555.48
PYR-32	0.89	low reliability	-1.50	moderate reliability	98.49	34	low reliability	95.20	-1.980	low reliability	-77.83
PYR-33	1.08	low reliability	-1.87	low reliability	99.35	34	low reliability	95.20	-1.841	low reliability	-144.91
PYR-34	1.67	moderate reliability	-1.93	moderate reliability	99.44	41	moderate reliability	94.21	-1.571	moderate reliability	-356.04
PYR-35	0.66	low reliability	-0.29	moderate reliability	75.45	17	low reliability	97.60	-2.247	low reliability	3.84
PYR-36	1.02	low reliability	-0.05	moderate reliability	57.34	20	low reliability	97.18	-0.698	low reliability	-3304.08
PYR-37	1.51	low reliability	0.88	low reliability	-263.08	41	low reliability	94.21	-0.482	low reliability	-5497.58
PYR-38	0.40	low reliability	-0.89	low reliability	93.83	34	low reliability	95.20	-2.065	low reliability	-46.22
PYR-39	1.02	low reliability	-0.69	moderate reliability	90.23	5	low reliability	99.29	-1.049	low reliability	-1417.05
PYR-40	2.50	experimental value	0.41	experimental value	-23.03	71	experimental value	89.97	-1.652	experimental value	-278.44

Table S6 Risk assessment of biological metabolic and transformation pathways of PAHs

Parent PAH	Pathway	Neurotoxicity	Immunotoxicity	Developmental toxicity	Genotoxicity	Carcinogenicity	Endocrine disrupting effects	Human health risk factor A	Phytotoxicity	Environmental risk factor B	BCF	Half-life	Persistence (soil)	Air Half-Life	Environmental risk C	RA_i	Rank	Risk assessment for PAHs	Rank
**ACE**	Microbial (bacteria) degradation	8	2	7	18	5	1	6.83	26	26	0	2	0	14	4.00	36.83	1	38.67	9
	Photolysis	0	1	2	2	0	0	0.83	1	1	0	0	0	0	0.00	1.83	2		
**ACY**	Microbial (bacteria) degradation	0	4	1	8	0	0	2.17	11	11	0	0	0	3	0.75	13.92	1	18.17	15
	Photolysis	0	2	2	2	0	0	1.00	3	3	0	0	0	1	0.25	4.25	2		
**ANT**	Photocatalytic degradation	0	3	4	2	1	0	1.67	4	4	0	0	0	4	1.00	6.67	3	39.08	8
	Microbial (fungus) degradation	1	1	2	4	4	0	2.00	10	10	0	0	0	6	1.50	13.50	2		
	Microbial (bacteria) degradation	4	3	9	10	4	1	5.17	9	9	5	2	0	12	4.75	18.92	1		
	Human metabolism	0	0	22	22	5	0	8.17	0	0	0	0	0	12	3.00	11.17	1		
**BaAN**	Biological (chick embryos) metabolism	1	1	1	2	1	0	1.00	1	1	0	0	0	0	0.00	2.00	5	33.42	11
	Photolysis	1	1	1	2	1	0	1.00	1	1	0	0	0	0	0.00	2.00	5		
	Microbial (microalgae) degradation	0	0	1	4	2	1	1.33	0	0	5	6	0	5	4.00	5.33	4		
	Microbial (fungi) degradation	1	0	6	6	2	0	2.50	1	1	0	0	0	14	3.50	7.00	2		
	Photocatalytic degradation	0	1	4	4	2	2	2.17	2	2	1	0	0	6	1.75	5.92	3		
**BaP**	Human metabolism	0	1	2	2	1	1	1.17	0	0	1	0	0	2	0.75	1.92	8	140.92	1
	Biological (fish) metabolism	0	1	2	2	1	1	1.17	0	0	1	0	0	2	0.75	1.92	8		
	Photolysis	4	1	11	8	8	4	6.00	8	8	4	8	0	28	10.00	24.00	2		

	Photocatalytic degradation	5	4	20	16	8	4	9.50	9	9	3	2	0	13	4.50	23.00	3		
	Resistance of plants	1	2	8	8	5	5	4.83	0	0	3	0	0	10	3.25	8.08	5		
	Microbial (fungi) degradation	1	1	6	6	3	3	3.33	1	1	1	2	0	4	1.75	6.08	6		
	Anaerobic biodegradation	2	0	0	12	5	0	3.17	3	3	7	8	0	17	8.00	14.17	4		
	Microbial (bacteria) degradation	6	8	42	42	22	12	22.00	21	21	6	2	0	45	13.25	56.25	1		
	Microbial (microalgae) degradation	0	2	8	8	4	2	4.00	0	0	0	0	0	6	1.50	5.50	7		
	Biological (mouse) metabolism	6	2	14	14	5	7	8.00	2	2	7	0	0	13	5.00	15.00	1		
	Human metabolism	4	2	8	8	4	4	5.00	0	0	4	0	0	6	2.50	7.50	2	34.92	10
**BbF**	Microbial (bacteria) degradation	1	1	3	6	3	4	3.00	2	2	3	1	0	6	2.50	7.50	2		
	Microbial (microalgae) degradation	1	0	0	4	3	2	1.67	1	1	0	3	0	6	2.25	4.92	4		
**BghiP**	Microbial (fungi) degradation	0	0	4	4	1	1	1.67	1	1	1	5	0	8	3.50	6.17	2	33.17	12
	Biological (rat) metabolism	12	9	26	22	11	10	15.00	8	8	5	0	0	11	4.00	27.00	1		
**BkF**	Biological (rat) metabolism	1	1	6	6	1	3	3.00	0	0	3	0	0	0	0.75	3.75	2	22.67	13
	Human metabolism	1	1	4	4	1	2	2.17	0	0	2	0	0	0	0.50	2.67	3		
	Microbial (microalgae) degradation	0	0	2	2	1	1	1.00	0	0	0	0	0	0	0.00	1.00	4		
	Microbial (bacteria) degradation	6	2	13	21	6	3	8.50	3	3	1	0	0	14	3.75	15.25	1		
**CHR**	Biological (fish) metabolism	0	0	0	4	1	1	1.00	1	1	0	0	0	4	1.00	3.00	3	19.50	14
	Human metabolism	0	1	2	2	1	1	1.17	1	1	1	0	0	0	0.25	2.42	4		
	Microbial (bacteria) degradation	0	0	1	5	3	2	1.83	3	3	0	0	0	10	2.50	7.33	1		
	Microbial (fungi) degradation	0	0	2	5	3	2	2.00	2	2	1	0	0	10	2.75	6.75	2		
**DahA**	Biological (rat) metabolism	0	0	2	2	1	1	1.00	0	0	1	0	0	2	0.75	1.75	2	11.17	16

	Microbial (fungi) degradation	0	0	6	5	2	0	2.17	3	3	3	2	0	12	4.25	9.42	1		
FLR	Biological (bovine) metabolism	1	3	4	6	1	1	2.67	0	0	0	0	0	3	0.75	3.42	3	54.33	7
	Human metabolism	1	3	4	6	1	1	2.67	0	0	0	0	0	3	0.75	3.42	3		
	Biological (fish) metabolism	1	3	4	6	1	1	2.67	0	0	0	0	0	3	0.75	3.42	3		
	Microbial (bacteria) degradation	5	12	21	22	9	1	11.67	17	17	0	0	0	30	7.50	36.17	1		
	Anaerobic biodegradation	0	0	0	0	0	0	0.00	1	1	0	0	0	2	0.50	1.50	7		
	Microbial (fungi) degradation	0	2	0	4	0	0	1.00	1	1	0	0	0	2	0.50	2.50	6		
	Microbial (microalgae) degradation	0	3	2	6	1	1	2.17	1	1	0	0	0	3	0.75	3.92	2		
FRT	Biological (fish) metabolism	4	2	20	8	6	0	6.67	0	0	3	2	0	22	6.75	13.42	3	87.25	3
	Human metabolism	0	0	2	2	1	0	0.83	0	0	1	0	0	2	0.75	1.58	5		
	Microbial (bacteria) degradation	7	2	33	48	23	1	19.00	19	19	4	10	0	49	15.75	53.75	1		
	Microbial (fungi) degradation	2	0	7	6	6	2	3.83	4	4	6	9	0	16	7.75	15.58	2		
	Microbial (microalgae) degradation	0	0	4	4	2	0	1.67	0	0	1	0	0	4	1.25	2.92	4		
IcdP	Biological (rat) metabolism	0	3	6	6	3	3	3.50	0	0	3	0	0	6	2.25	5.75	5	58.58	6
	Biological (mouse) metabolism	0	6	14	14	7	7	8.00	0	0	3	0	0	12	3.75	11.75	2		
	Human metabolism	0	1	2	2	1	1	1.17	0	0	1	0	0	2	0.75	1.92	6		
	Microbial (fungi) degradation	1	3	2	4	4	2	2.67	1	1	3	2	0	18	5.75	9.42	3		
	Microbial (bacteria) degradation	0	8	26	22	10	7	12.17	0	0	4	6	0	25	8.75	20.92	1		
	Biological (materials) metabolism	0	5	10	10	5	5	5.83	0	0	4	0	0	8	3.00	8.83	4		
NAP	Human metabolism	14	17	4	16	3	4	9.67	16	16	0	0	0	4	1.00	26.67	1	85.33	4
	Biological (mouse) metabolism	5	7	6	2	1	0	3.50	4	4	0	0	0	0	0.00	7.50	5		

	Biological (fish) metabolism	3	3	0	4	1	1	2.00	3	3	0	0	0	0	0.00	5.00	6		
	Biological (bovine) metabolism	3	3	0	4	1	1	2.00	3	3	0	0	0	0	0.00	5.00	6		
	Anaerobic biodegradation	9	10	2	4	0	1	4.33	9	9	0	0	0	0	0.00	13.33	3		
	Microbial (bacteria) degradation	10	8	0	8	4	2	5.33	14	14	0	0	0	2	0.50	19.83	2		
	Microbial (microalgae) degradation	4	4	0	6	1	0	2.50	5	5	0	0	0	2	0.50	8.00	4		
	Biological (marine teleosts) metabolism	1	2	0	4	1	0	1.33	2	2	0	0	0	2	0.50	3.83	9		
	Biological (bovine) metabolism	2	4	0	10	4	1	3.50	3	3	0	0	0	8	2.00	8.50	7	128.25	2
	Human metabolism	1	3	0	8	3	1	2.67	2	2	0	0	0	6	1.50	6.17	8		
	Photolysis	4	8	1	6	2	0	3.50	5	5	0	2	0	6	2.00	10.50	5		
**PHE**	Microbial (bacteria) degradation	19	22	6	40	11	10	18.00	27	27	0	0	0	22	5.50	50.50	1		
	Coupled photocatalysis and biodegradation	5	6	3	5	4	0	3.83	5	5	0	2	0	6	2.00	10.83	4		
	Microbial (microalgae) degradation	8	8	13	7	5	3	7.33	9	9	0	0	0	5	1.25	17.58	2		
	Coupled plant and microbial catabolisms	6	5	0	7	2	1	3.50	7	7	0	0	0	4	1.00	11.50	3		
	Microbial (fungi) degradation	2	2	4	10	4	4	4.33	3	3	0	0	0	6	1.50	8.83	6		
	Biological (bovine) metabolism	1	0	2	2	1	0	1.00	1	1	1	0	0	2	0.75	2.75	6	79.33	5
**PYR**	Biological (fish) metabolism	1	0	2	2	1	0	1.00	1	1	1	0	0	2	0.75	2.75	6		
	Human metabolism	3	0	6	6	3	2	3.33	1	1	1	0	0	6	1.75	6.08	4		
	Resistance of plants	2	0	4	7	3	1	2.83	1	1	1	0	0	8	2.25	6.08	4		

Microbial (fungi) degradation	3	1	9	6	4	0	3.83	2	2	1	0	0	10	2.75	8.58	3
Microbial (bacteria) degradation	11	3	28	33	13	4	15.33	11	11	1	1	0	38	10.00	36.33	1
Photocatalytic degradation	3	1	5	14	5	2	5.00	4	4	2	3	0	15	5.00	14.00	2
Microbial (microalgae) degradation	1	0	2	2	1	0	1.00	1	1	1	0	0	2	0.75	2.75	6

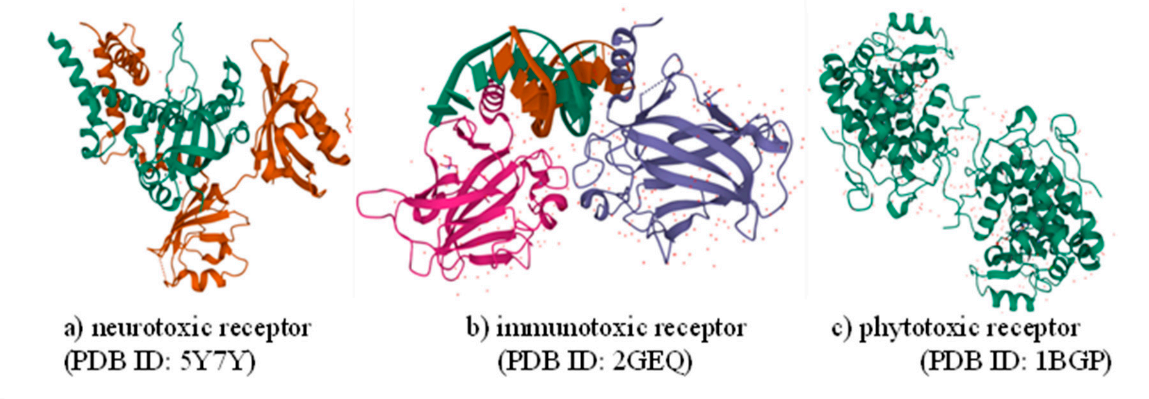

Figure S1 Three-dimensional structure of PAHs' neurotoxic, immunotoxic and phytotoxic receptor

Reference

1. MAllick, S. Biodegradation of acenaphthene by Sphingobacterium sp. strain RTSB involving trans-3-carboxy-2-hydroxybenzylidenepyruvic acid as a metabolite. *Chemosphere* **2019**, *219*, 748–755, doi:10.1016/j.chemosphere.2018.12.046.
2. Marquès, M.; Mari, M.; Audí-Miró, C.; Sierra, J.; Soler, A.; Nadal, M.; Domingo, J.L. Climate change impact on the PAH photodegradation in soils: Characterization and metabolites identification. *Environ. Int.* **2016**, *89–90*, 155–165, doi:10.1016/j.envint.2016.01.019.
3. Poonthrigpun, S.; Pattaragulwanit, K.; Paengthai, S.; Kriangkripipat, T.; Juntongjin, K.; Thaniyavarn, S.; Petsom, A.; Pinphanichakarn, P. Novel intermediates of acenaphthylene degradation by Rhizobium sp. strain CU-A1: Evidence for naphthalene-1,8-dicarboxylic acid metabolism. *Appl. Environ. Microbiol.* **2006**, *72*, 6034–6039, doi:10.1128/AEM.00897-06.
4. Kou, J.; Li, Z.; Yuan, Y.; Zhang, H.; Wang, Y.; Zou, Z. Visible-light-induced photocatalytic oxidation of polycyclic aromatic hydrocarbons over tantalum oxynitride photocatalysts. *Environ. Sci. Technol.* **2009**, *43*, 2919–2924, doi:10.1021/es802940a.
5. Da Silva, M.; Esposito, E.; Moody, J.D.; Canhos, V.P.; Cerniglia, C.E. Metabolism of aromatic hydrocarbons by the filamentous fungus Cyclothyrium sp. *Chemosphere* **2004**, *57*, 943–952, doi:10.1016/j.chemosphere.2004.07.051.
6. Hadibarata, T.; Khudhair, A.B.; Salim, M.R. Breakdown products in the metabolic pathway of anthracene degradation by a ligninolytic fungus Polyporus sp. S133. *Water. Air. Soil Pollut.* **2012**, *223*, 2201–2208, doi:10.1007/s11270-011-1016-1.
7. Tarafdar, A.; Sinha, A.; Masto, R.E. Biodegradation of anthracene by a newly isolated bacterial strain, Bacillus thuringiensis AT.ISM.1, isolated from a fly ash deposition site. *Lett. Appl. Microbiol.* **2017**, *65*, 327–334, doi:10.1111/lam.12785.
8. Sahali, Y.; Kidd, L.C.R.; Skipper, P.L.; Tannenbaum, S.R. Metabolism of benz[a]anthracene by human liver microsomes. *Cancer Lett.* **1994**, *83*, 299–303, doi:10.1016/0304-3835(94)90333-6.
9. Westman, O.; Larsson, M.; Venizelos, N.; Hollert, H.; EngwAll, M. An oxygenated metabolite of benzo[a]pyrene increases hepatic β-oxidation of fatty acids in chick embryos. *Environ. Sci. Pollut. Res.* **2014**, *21*, 6243–6251, doi:10.1007/s11356-013-2471-6.
10. Zhao, S.; Jia, H.; Nulaji, G.; Gao, H.; Wang, F.; Wang, C. Photolysis of polycyclic aromatic hydrocarbons (PAHs) on Fe^{3+}-montmorillonite surface under visible light: Degradation kinetics, mechanism, and toxicity assessments. *Chemosphere* **2017**, *184*, 1346–1354, doi:10.1016/j.chemosphere.2017.06.106.
11. Luo, J.; Deng, J.; Cui, L.; Chang, P.; Dai, X.; Yang, C.; Li, N.; Ren, Z.; Zhang, X. The potential assessment of green alga Chlamydomonas reinhardtii CC-503 in the biodegradation of benz(a)anthracene and the related mechanism analysis. *Chemosphere*

2020, *249*, 126097, doi:10.1016/j.chemosphere.2020.126097.

12. Cajthaml, T.; Erbanová, P.; Šašek, V.; Moeder, M. Breakdown products on metabolic pathway of degradation of benz[a]anthracene by a ligninolytic fungus. *Chemosphere* **2006**, *64*, 560–564, doi:10.1016/j.chemosphere.2005.11.034.
13. Rachna; Rani, M.; Shanker, U. Sunlight mediated improved Photocatalytic degradation of carcinogenic benz[a]anthracene and benzo[a]pyrene by zinc oxide encapsulated hexacyanoferrate nanocomposite. *J. Photochem. Photobiol. A Chem.* **2019**, *381*, 111861, doi:10.1016/j.jphotochem.2019.111861.
14. Tombolini, F.; Pigini, D.; Tranfo, G.; Paci, E.; Carosi, I.; Marini, F.; Bauleo, L.; Ancona, C.; Forastiere, F. Levels of urinary metabolites of four PAHs and cotinine determined in 1016 volunteers living in Central Italy. *Environ. Sci. Pollut. Res.* **2018**, *25*, 28772–28779, doi:10.1007/s11356-018-1650-x.
15. Yang, L.; Zhou, Y.; Sun, H.; Lai, H.; Liu, C.; Yan, K.; Yuan, J.; Wu, T.; Chen, W.; Zhang, X. Dose-response relationship between polycyclic aromatic hydrocarbon metabolites and risk of diabetes in the general Chinese population. *Environ. Pollut.* **2014**, *195*, 24–30, doi:10.1016/j.envpol.2014.08.012.
16. Lourenço, D.; Silva, L.J.G.; Lino, C.M.; Morais, S.; Pena, A. SPE-LC-FD determination of polycyclic aromatic hydrocarbon monohydroxy derivatives in cephalopods. *J. Agric. Food Chem.* **2014**, *62*, 2685–2691, doi:10.1021/jf4056852.
17. Wang, X.H.; Hong, H.S.; Mu, J.L.; Lin, J.Q.; Wang, S.H. Polycyclic aromatic hydrocarbon (PAH) metabolites in marine fishes as a specific biomarker to indicate PAH pollution in the marine coastal environment. *J. Environ. Sci. Heal. - Part A Toxic/Hazardous Subst. Environ. Eng.* **2008**, *43*, 219–226, doi:10.1080/10934520701792662.
18. Gupta, H.; Gupta, B. Photocatalytic degradation of polycyclic aromatic hydrocarbon benzo[a]pyrene by iron oxides and identification of degradation products. *Chemosphere* **2015**, *138*, 924–931, doi:10.1016/j.chemosphere.2014.12.028.
19. Ill, P. Research Articles (Series): Impact Studies on PAHs. **2004**, *1*, 22–32.
20. Shu-yan, Z.; Pei-jun, L.I.; Xiao-cai, Y.U.; Kun, S.H.I.; Hui, Z.; Jing, C. Degradation of metabolites of benzo[a]pyrene by coupling Penicillium chrysogenum with KMn04. *J. Environ. Sci.* **2007**, *19*, 238–243.
21. Qin, W.; Fan, F.; Zhu, Y.; Huang, X.; Ding, A.; Liu, X.; Dou, J. Anaerobic biodegradation of benzo(a)pyrene by a novel Cellulosimicrobium cellulans CWS2 isolated from polycyclic aromatic hydrocarbon-contaminated soil. *Brazilian J. Microbiol.* **2018**, *49*, 258–268, doi:10.1016/j.bjm.2017.04.014.
22. Chen, S.; Yin, H.; Ye, J.; Peng, H.; Zhang, N.; He, B. Effect of copper(II) on biodegradation of benzo[a]pyrene by Stenotrophomonas maltophilia. *Chemosphere* **2013**, *90*, 1811–1820, doi:10.1016/j.chemosphere.2012.09.009.
23. Seo, J.S.; Keum, Y.S.; Li, Q.X. *Bacterial degradation of aromatic compounds*; 2009; Vol.

6; ISBN 1808956354.

24. Ghosal, D.; Ghosh, S.; Dutta, T.K.; Ahn, Y. Current State of Knowledge in Microbial Degradation of Polycyclic Aromatic Hydrocarbons (PAHs): A Review. *Front. Microbiol.* **2016**, *0*, 1369, doi:10.3389/FMICB.2016.01369.

25. Geddie, J.E.; Amin, S.; Huie, K.; Hecht, S.S. Formation and tumorigenicity of benzo[b]fluoranthene metabolites in mouse epidermis. *Carcinogenesis* **1987**, *8*, 1579–1584, doi:10.1093/carcin/8.11.1579.

26. Palazzi, P.; Mezzache, S.; Bourokba, N.; Hardy, E.M.; Schritz, A.; Bastien, P.; Emond, C.; Li, J.; Soeur, J.; Appenzeller, B.M.R. Exposure to polycyclic aromatic hydrocarbons in women living in the Chinese cities of BaoDing and Dalian revealed by hair analysis. *Environ. Int.* **2018**, *121*, 1341–1354, doi:10.1016/j.envint.2018.10.056.

27. Story, S.P.; Kline, E.L.; Hughes, T.A.; Riley, M.B.; Hayasaka, S.S. Degradation of aromatic hydrocarbons by Sphingomonas paucimobilis strain EPA505. *Arch. Environ. Contam. Toxicol.* **2004**, *47*, 168–176, doi:10.1007/s00244-004-3069-2.

28. Hernández Blanco, F.J.; García de Llasera, M.P. Monitoring dihydrodiol polyaromatic hydrocarbon metabolites produced by the freshwater microalgae Selenastrum capricornutum. *Chemosphere* **2016**, *158*, 80–90, doi:10.1016/j.chemosphere.2016.05.065.

29. Mandal, S. K. and Das, N. Biodegradation of perylene and benzo [ ] perylene (5-6 rings) using yeast ghi consortium : Kinetic study, enzyme analysis and degradation pathway. **2016**, *39*, 5–15.

30. Platt, K.L.; Grupe, S. Microsomal biotransformation of benzo[ghi]perylene, a mutagenic polycyclic aromatic hydrocarbon without a "classic" bay region. *Chem. Res. Toxicol.* **2005**, *18*, 700–710, doi:10.1021/tx049698a.

31. Grova, N.; Faÿs, F.; Hardy, E.M.; Appenzeller, B.M.R. New insights into urine-based assessment of polycyclic aromatic hydrocarbon-exposure from a rat model: Identification of relevant metabolites and influence of elimination kinetics. *Environ. Pollut.* **2017**, *228*, 484–495, doi:10.1016/j.envpol.2017.03.060.

32. Maeda, A.H.; Nishi, S.; Hatada, Y.; Ozeki, Y.; Kanaly, R.A. Biotransformation of the high-molecular weight polycyclic aromatic hydrocarbon (PAH) benzo[k]fluoranthene by Sphingobium sp. strain KK22 and identification of new products of non-alternant PAH biodegradation by liquid chromatography electrospray ionizatio. *Microb. Biotechnol.* **2014**, *7*, 114–129, doi:10.1111/1751-7915.12102.

33. Pampanin, D.M.; Le Goff, J.; Skogland, K.; Marcucci, C.R.; Øysæd, K.B.; Lorentzen, M.; Jørgensen, K.B.; Sydnes, M.O. Biological effects of polycyclic aromatic hydrocarbons (PAH) and their first metabolic products in in vivo exposed Atlantic cod (Gadus morhua). *J. Toxicol. Environ. Heal. - Part A Curr. Issues* **2016**, *79*, 633–646, doi:10.1080/15287394.2016.1171993.

34. Al Farraj, D.A.; Hadibarata, T.; Yuniarto, A.; Syafiuddin, A.; Surtikanti, H.K.; Elshikh, M.S.;

Al Khulaifi, M.M.; Al-Kufaidy, R. Characterization of pyrene and chrysene degradation by halophilic Hortaea sp. B15. *Bioprocess Biosyst. Eng.* **2019**, *0*, 0, doi:10.1007/s00449-019-02096-8.

35. Nayak, A.S.; Sanjeev Kumar, S.; Santosh Kumar, M.; Anjaneya, O.; Karegoudar, T.B. A catabolic pathway for the degradation of chrysene by Pseudoxanthomonas sp. PNK-04. *FEMS Microbiol. Lett.* **2011**, *320*, 128–134, doi:10.1111/j.1574-6968.2011.02301.x.
36. Hidayat, A.; Tachibana, S.; Itoh, K. Determination of chrysene degradation under saline conditions by Fusarium sp. F092, a fungus screened from nature. *Fungal Biol.* **2012**, *116*, 706–714, doi:10.1016/j.funbio.2012.04.004.
37. GuntupAlli, S.; Thunuguntla, V.B.S.C.; Chalasani, L.M.; Rao, C. V.; Bondili, J.S. Degradation and Metabolite Profiling of Benz (a) Anthracene, Dibenz (a, h) Anthracene and Indeno [1, 2, 3-cd] Pyrene by Aspergillus terricola. *Polycycl. Aromat. Compd.* **2019**, *39*, 84–92, doi:10.1080/10406638.2016.1262878.
38. Zhu, H.; Wang, L.; Liu, C.; Stryker, Z.; Loganathan, B.G.; Kannan, K. Phthalate Metabolites, Hydroxy-Polycyclic Aromatic Hydrocarbons, and Bisphenol Analogues in Bovine Urine Collected from China, India, and the United States. *Environ. Sci. Technol.* **2019**, doi:10.1021/acs.est.9b04178.
39. Johnson-Restrepo, B.; Olivero-Verbel, J.; Lu, S.; Guette-Fernández, J.; Baldiris-Avila, R.; O'Byrne-Hoyos, I.; Aldous, K.M.; Addink, R.; Kannan, K. Polycyclic aromatic hydrocarbons and their hydroxylated metabolites in fish bile and sediments from coastal waters of Colombia. *Environ. Pollut.* **2008**, *151*, 452–459, doi:10.1016/j.envpol.2007.04.011.
40. Tsai, J.C.; Kumar, M.; Lin, J.G. Anaerobic biotransformation of fluorene and phenanthrene by sulfate-reducing bacteria and identification of biotransformation pathway. *J. Hazard. Mater.* **2009**, *164*, 847–855, doi:10.1016/j.jhazmat.2008.08.101.
41. Garon, D.; Krivobok, S.; Seigle-Murandi, F. Fungal degradation of fluorene. *Chemosphere* **2000**, *40*, 91–97, doi:10.1016/S0045-6535(99)00250-7.
42. Ke, L.; Luo, L.; Wang, P.; Luan, T.; Tam, N.F.Y. Effects of metals on biosorption and biodegradation of mixed polycyclic aromatic hydrocarbons by a freshwater green alga Selenastrum capricornutum. *Bioresour. Technol.* **2010**, *101*, 6950–6961, doi:10.1016/j.biortech.2010.04.011.
43. Hillenweck, A.; Canlet, C.; Mauffret, A.; Debrauwer, L.; Claireaux, G.; Cravedi, J.P. Characterization of biliary metabolites of fluoranthene in the common sole (Solea Solea). *Environ. Toxicol. Chem.* **2008**, *27*, 2575–2581, doi:10.1897/08-180.1.
44. Lee, S.E.; Seo, J.S.; Keum, Y.S.; Lee, K.J.; Li, Q.X. Fluoranthene metabolism and associated proteins in Mycobacterium sp. JS14. *Proteomics* **2007**, *7*, 2059–2069, doi:10.1002/pmic.200600489.
45. Garcia, A.C.F.S.; Birolli, W.G.; Araújo, B.R.; Marques, C.G.; Barbosa-Junior, A.M.; Diniz,

L.E.C.; Porto, A.L.M.; Romão, L.P.C. Biodegradation of the polycyclic aromatic hydrocarbon fluoranthene by fungi strains from a brazilian tropical peat. *J. Braz. Chem. Soc.* **2021**, *32*, 1874–1883, doi:10.21577/0103-5053.20210078.

46. Luan, T.; Fang, S.; Zhong, Y.; Lin, L.; Chan, S.M.N.; Lan, C.; Tam, N.F.Y. Determination of hydroxy metabolites of polycyclic aromatic hydrocarbons by fully automated solid-phase microextraction derivatization and gas chromatography-mass spectrometry. *J. Chromatogr. A* **2007**, *1173*, 37–43, doi:10.1016/j.chroma.2007.10.038.
47. Rice, J.E.; Hosted, T.J.; Defloria, M.C.; Lavoie, E.J.; Fischer, D.L.; Wiley, J.C. Tumor-initiating activity of major in vivo metabolites of indeno[1, 2, 3-CD]pyrene on mouse skin. *Carcinogenesis* **1986**, *7*, 1761–1764, doi:10.1093/carcin/7.10.1761.
48. Miao, L.L.; Qu, J.; Liu, Z.P. Hydroxylation at Multiple Positions Initiated the Biodegradation of Indeno[1,2,3-cd]Pyrene in Rhodococcus aetherivorans IcdP1. *Front. Microbiol.* **2020**, *11*, 1–13, doi:10.3389/fmicb.2020.568381.
49. Jacob, J.; Grimmer, G. Capillary gas chromatographical analysis and mass spectrometric identification of polycyclic aromatic hydrocarbon metabolites from biological materials. *Rev. Anal. Chem.* **1987**, *9*, 49–89, doi:10.1515/REVAC.1987.9.1.49.
50. Nilsson, R.; Antić, R.; Berni, A.; DAllner, G.; Dettbarn, G.; Gromadzinska, J.; Joksić, G.; Lundin, C.; Palitti, F.; Prochazka, G.; et al. Exposure to polycyclic aromatic hydrocarbons in women from Poland, Serbia and Italy-relation between PAH metabolite excretion, DNA damage, diet and genotype (the EU DIEPHY project). *Biomarkers* **2013**, *18*, 165–173, doi:10.3109/1354750X.2012.762807.
51. Tang, C.; Tan, J.; Fan, R.; Zhao, B.; Tang, C.; Ou, W.; Jin, J.; Peng, X. Quasi-targeted analysis of hydroxylation-related metabolites of polycyclic aromatic hydrocarbons in human urine by liquid chromatography–mass spectrometry. *J. Chromatogr. A* **2016**, *1461*, 59–69, doi:10.1016/j.chroma.2016.07.051.
52. Cho, T.M.; Rose, R.L.; Hodgson, E. In vitro metabolism of naphthalene by human liver microsomal cytochrome P450 enzymes. *Drug Metab. Dispos.* **2006**, *34*, 176–183, doi:10.1124/dmd.105.005785.
53. Ayala, D.C.; Morin, D.; Buckpitt, A.R. Simultaneous quantification of multiple urinary naphthalene metabolites by liquid chromatography tandem mass spectrometry. *PLoS One* **2015**, *10*, 1–16, doi:10.1371/journal.pone.0121937.
54. Kleemann, R.; Meckenstock, R.U. Anaerobic naphthalene degradation by Gram-positive, iron-reducing bacteria. *FEMS Microbiol. Ecol.* **2011**, *78*, 488–496, doi:10.1111/j.1574-6941.2011.01193.x.
55. Weyrauch, P.; Heker, I.; Zaytsev, A. V.; von Hagen, C.A.; Arnold, M.E.; Golding, B.T.; Meckenstock, R.U. The 5,6,7,8-Tetrahydro-2-naphthoyl-coenzyme a reductase reaction in the anaerobic degradation of naphthalene and identification of downstream metabolites. *Appl. Environ. Microbiol.* **2020**, *86*, doi:10.1128/AEM.00996-20.

56. Cerniglia, C.E.; Van Baalen, C.; Gibson, D.T. Metabolism of naphthalene by the cyanobacterium Oscillatoria sp., strain JCM. *J. Gen. Microbiol.* **1980**, *116*, 485–494, doi:10.1099/00221287-116-2-485.
57. Pulster, E.L.; Main, K.; Wetzel, D.; Murawski, S. Species-specific metabolism of naphthalene and phenanthrene in 3 species of marine teleosts exposed to Deepwater Horizon crude oil. *Environ. Toxicol. Chem.* **2017**, *36*, 3168–3176, doi:10.1002/etc.3898.
58. Cai, H.; Sun, L.; Wang, Y.; Song, T.; Bao, M.; Yang, X. Unprecedented efficient degradation of phenanthrene in water by intimately coupling novel ternary composite Mn3O4/MnO2-Ag3PO4 and functional bacteria under visible light irradiation. *Chem. Eng. J.* **2019**, *369*, 1078–1092, doi:10.1016/j.cej.2019.03.143.
59. Safonova, E.; Kvitko, K.; Kuschk, P.; Möder, M.; Reisser, W. Biodegradation of phenanthrene by the green alga Scenedesmus obliquus ES-55. *Eng. Life Sci.* **2005**, *5*, 234–239, doi:10.1002/elsc.200520077.
60. Muratova, A.; Dubrovskaya, E.; Golubev, S.; Grinev, V.; Chernyshova, M.; Turkovskaya, O. The coupling of the plant and microbial catabolisms of phenanthrene in the rhizosphere of Medicago sativa. *J. Plant Physiol.* **2015**, *188*, 1–8, doi:10.1016/j.jplph.2015.07.014.
61. Ruddock, P.J.; Bird, D.J.; McEvoy, J.; Peters, L.D. Bile metabolites of polycyclic aromatic hydrocarbons (PAHs) in European eels Anguilla anguilla from United Kingdom estuaries. *Sci. Total Environ.* **2003**, *301*, 105–117, doi:10.1016/S0048-9697(02)00292-9.
62. Gupta, B.; Gupta, H. Iron Oxide Mediated Degradation of Mutagen Pyrene and Determination of Degradation Products. *Int. J. Environ. Sci. Dev.* **2015**, *6*, 908–912, doi:10.7763/ijesd.2015.v6.720.
